# Treatment Rates for Mental Disorders Among Children and Adolescents: A Systematic Review and Meta-Analysis

**DOI:** 10.1001/jamanetworkopen.2023.38174

**Published:** 2023-10-18

**Authors:** Sifan Wang, Qiongxian Li, Jin Lu, Hailiang Ran, Yusan Che, Die Fang, Xuemeng Liang, Hao Sun, Lin Chen, Junwei Peng, Yuanyu Shi, Yuanyuan Xiao

**Affiliations:** 1 NHC Key Laboratory of Drug Addiction Medicine, Division of Epidemiology and Health Statistics, School of Public Health, Kunming Medical University, Kunming, Yunnan, China; 2 Psychiatry Department, The First Affiliated Hospital, Kunming Medical University, Kunming, Yunnan, China; 3 Mental Health Institute of Yunnan, The First Affiliated Hospital, Kunming Medical University, Kunming, Yunnan, China; 4 Yunnan Clinical Research Center for Mental Health, Kunming, Yunnan, China; 5 Key Library in Public Health and Disease Prevention and Control, Yunnan Provincial Department of Education, Kunming, Yunnan, China

## Abstract

**Importance:**

Mental disorders among children and adolescents are global health concerns. Published studies have provided discordant results regarding treatment rates for mental disorders among youths.

**Objective:**

To estimate combined treatment rates for several common psychiatric disorders among children and adolescents.

**Data Sources:**

PubMed, Web of Science, PsycINFO, Scopus, and Embase were searched from database inception until September 23, 2022, and supplemented with hand-searching of reference lists.

**Study Selection:**

Included studies were those that used validated methods to report treatment rates for any mental disorder, depressive disorders, anxiety disorders, attention-deficit/hyperactivity disorder (ADHD), and behavior disorders among children and adolescents.

**Data Extraction and Synthesis:**

This study followed the Preferred Reporting Items for Systematic Reviews and Meta-Analyses (PRISMA) reporting guideline. Two reviewers independently assessed study eligibility, extracted data, and scored quality. Studies with a Joanna Briggs Institute score of 5 or more were included in the meta-analysis. Treatment rates were pooled using random-effects models. Subgroup analyses were performed to investigate the association with treatment rates of factors, such as year of data collection, World Health Organization region, age, income level, timeframe of diagnosis, informant source, service type, sample origin, and internalizing or externalizing disorder.

**Main Outcomes and Measures:**

Treatment rates for mental disorders among children and adolescents were the main outcomes, measured as percentage estimates.

**Results:**

Forty studies were included in the analysis, comprising 310 584 children and adolescents, with boys accounting for 39% of participants (sex was not reported in 10 studies). The pooled treatment rate was 38% (95% CI, 30%-45%) for any mental disorder, 36% (95% CI, 29%-43%) for depressive disorders, 31% (95% CI, 21%-42%) for anxiety disorders, 58% (95% CI, 42%-73%) for ADHD, and 49% (95% CI, 35%-64%) for behavior disorders. Age, income level, and region were significantly associated with the combined treatment rates of mental disorders in children and adolescents. The treatment rate for depressive disorders was higher among adolescents than children (36% [95% CI, 25%-46%] vs 11% [95% CI, 0%-25%]), whereas the treatment rate for anxiety disorders was higher among children than adolescents (64% [95% CI, 52%-75%] vs 20% [95% CI, 9%-30%]). The treatment rate for any mental disorder in lower-middle income countries was 6% (95% CI, 2%-14%), in upper-middle income countries was 24% (95% CI, 2%-47%), and in high-income countries was 43% (95% CI, 35%-52%). For depressive disorders, treatment rates were higher in the Americas (40% [95% CI, 30%-51%]) than in Europe (28% [95% CI, 13%-43%]) and the Western Pacific region (6% [95% CI, 1%-16%]).

**Conclusions and Relevance:**

This study suggests that, in general, the treatment rates for mental disorders among children and adolescents were low, especially for depression and anxiety. Targeted intervention policies and effective measures should be designed and implemented to improve treatment rates of psychiatric disorders among youths.

## Key Points

**Question:**

What are the treatment rates for mental disorders among children and adolescents?

**Findings:**

In this meta-analysis of 40 studies including 310 584 children and adolescents, the combined treatment rate was 38% (95% CI, 30%-45%) for any mental disorder, 36% (95% CI, 29%-43%) for depressive disorders, 31% (95% CI, 21%-42%) for anxiety disorders, 58% (95% CI, 42%-73%) for attention-deficit/hyperactivity disorder, and 49% for behavior disorders (95% CI, 35%-64%). Age, income level, and region were significantly associated with treatment rates for mental disorders among youths.

**Meaning:**

This study suggests that the treatment rates of mental disorders among children and adolescents were generally low, especially for depression and anxiety; targeted interventions are needed to improve this situation.

## Teaser

This meta-analysis of 40 studies estimates combined treatment rates for several common psychiatric disorders among children and adolescents.

## Introduction

Over the past several decades, the prevalence of mental disorders among children and adolescents has been increasing substantially, which has caused a heavy burden on the public.^1^ Anxiety, depression, attention-deficit/hyperactivity disorder (ADHD), and behavior disorders, such as oppositional-defiant disorder and conduct disorders, are the most common mental disorders among children and adolescents: a meta-analysis showed that the worldwide pooled prevalence reached 7% for anxiety disorders, 3% for depressive disorders, 3% for ADHD, and 6% for behavior disorders.^2^ The prevalence of mental disorders varies considerably in different regions: 20% in North America, 12% in Europe and Asia, and 8% in Africa. Sex and age differences are also apparent: for instance, boys are more likely than girls to receive a diagnosis of ADHD and adolescents are more susceptible than children to affective disorders and behavior disorders.^3^

Among children and adolescents, mental disorders are associated with a variety of negative outcomes, such as absenteeism, substance abuse, and suicidal behaviors.^4,5,6^ Moreover, the detrimental effect of early initiated mental disorders can extend into adulthood: longitudinal studies found that approximately 75% of psychiatric diseases diagnosed among adults had roots in childhood or adolescence.^7^ Timely and effective treatment of mental disorders significantly reduces the risk of subsequent negative outcomes and saves related health costs.^8^ Currently, evidence-based pharmacologic and nonpharmacologic treatments have been used in managing mental disorders among children and adolescents. However, owing to concerns regarding safety and adverse effects, the use of medication among children and adolescents is still controversial.^9^ Nonpharmacologic methods have been more widely used in this age group. For example, abundant studies have proven that family-centered psychotherapies that require the involvement of parents are critical in the therapeutic success of mental disorders among children and adolescents.^10,11,12^

However, among children and adolescents with mental disorders, only a small fraction have received treatment.^13^ In the US, approximately half of all children with mental health problems have received mental health services^14^; in the Netherlands, the treatment rate was 21%.^15^ Data on treatment rates in low- and middle-income countries (LMICs) are still limited, but available evidence suggests an overall treatment rate of less than 20% in upper-middle income countries (UMICs).^16,17^ However, published studies have reported highly variant treatment rates for mental disorders among children and adolescents, which causes confusion in understanding the true severity of the problem. For instance, the treatment rate for depressive disorders was reported as less than 10% in a study from China,^18^ while the rate was reported as 64% in a US study.^19^ In this meta-analysis, we aim to synthesize currently available evidence of high quality to estimate treatment rates for several commonly seen mental disorders among children and adolescents.

## Methods

This study was registered in PROSPERO (CRD42023398984) and performed according to the Preferred Reporting Items for Systematic Reviews and Meta-Analyses (PRISMA) reporting guideline.

### Retrieval Strategy and Eligibility Criteria

A systematic review of the literature was conducted using PubMed, Web of Science, PsycINFO, Scopus, and Embase from database inception to September 23, 2022, and the reference search was conducted for the included literature simultaneously. The search process and terms used are listed in eMethods 1 and the eAppendix in Supplement 1. Study inclusion criteria were (1) the upper limit of the age range was 18 years (to avoid missing pertinent studies, articles that did not specify the age range but instead reported a mean age of <18 years were also included); (2) treatment rates for mental disorders were reported; (3) diagnosis of mental disorder was based on well-accepted diagnostic methods; and (4) the article was published in English. There were no restrictions on study design.

### Data Extraction

A standard information extraction table was designed. Two of us (S.W. and Q.L.) independently extracted relevant information from all included studies, and the results were compared carefully; if any inconsistencies in extracted data were found, the 2 authors checked the full text of the relevant studies together, to see whether consensus could be reached after discussion. If they failed to reach a consensus, a senior researcher (Y.X.) performed the data extraction again.

### Exposure and Outcome

Exposure included the following commonly seen mental disorders among children and adolescents: any mental disorder, depressive disorders, anxiety disorders, ADHD, and behavior disorders. We included only studies that conducted diagnostic interviews based on the third, fourth, and fifth editions of the *Diagnostic and Statistical Manual of Mental Disorders* or the *International Classification of Diseases, Ninth Revision* or the *International Statistical Classification of Diseases and Related Health Problems, Tenth Revision*. Depressive disorders included major depressive disorder, destructive mood disorder, and dysthymia. Anxiety disorders included the full spectrum of diagnoses, such as generalized anxiety disorder, panic disorder, and posttraumatic stress disorder. Behavior disorders included oppositional defiant disorder and conduct disorder. Details of definitions and diagnostic tools for included mental disorders are in eMethods 2 in Supplement 1.

The major outcome was the proportion of diagnosed patients who received treatment or medical services. *Treatment seeking* was defined as use of treatment services for a mental health problem. Services included general medical services delivered by health care clinicians or health professionals, specialist mental health services delivered by mental health professionals, and school-based psychological counseling or treatment services. Seeking help from informal channels was excluded.

### Study Quality Assessment

We used the 9-item Joanna Briggs Institute (JBI) Checklist for Prevalence Studies to evaluate the quality of the included studies.^20^ Each item in the JBI Checklist was assigned 1 point for meeting the criterion, with a maximum score of 9 points. An overall score of 5 or more indicates satisfactory quality.^21^

### Statistical Analysis

Analyses were conducted using the “meta” and “forestplot” packages in R, version 4.2.2 (R Group for Statistical Computing). Forest plots were used to display the results graphically. A random-effects meta-analysis model was used to combine treatment rates. Heterogeneity was assessed using the Cochran *Q* test and the *I^2^* index.^22^ The risk of publication bias was evaluated by visual inspection of a funnel plot, and further checked by the Egger test and a nonparametric trim-and-fill method.^23^ A series of stratified analyses were done to explore the origins of heterogeneity, in which the following factors were considered: year of data collection, region, age, income level, timeframe of diagnosis, informant sources, health service type, sample source, and internalizing or externalizing disorders. Statistical significance was set as *P* < .05, 2-tailed. The *P* value for all secondary subgroup analyses was adjusted using the Bonferroni correction. Sensitivity analyses were further performed to check the robustness of combined estimations.

## Results

### Study Selection

**Figure 1.**
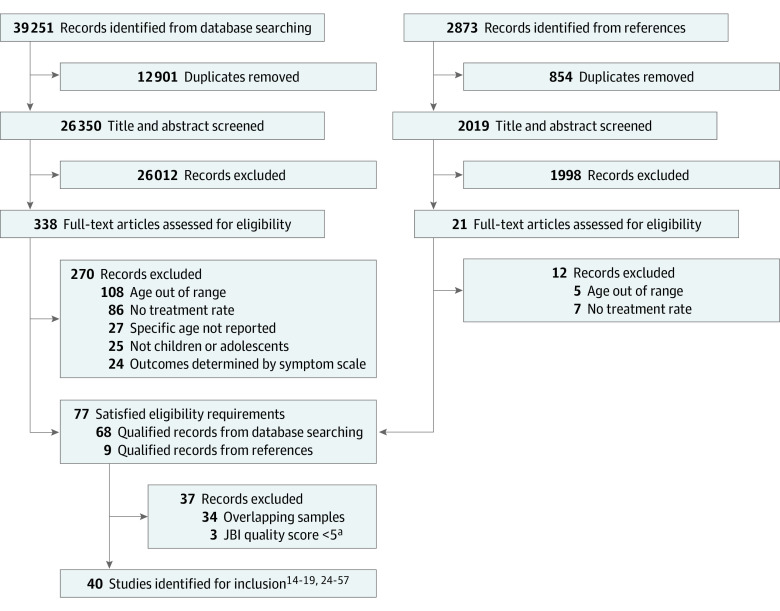
PRISMA Flow Diagram ^a^The Joanna Briggs Institute (JBI) quality score was evaluated by the JBI checklist for prevalence studies.

The procedure of literature screening is shown in a PRISMA flowchart (Figure 1).^14,15,16,17,18,19,24,25,26,27,28,29,30,31,32,33,34,35,36,37,38,39,40,41,42,43,44,45,46,47,48,49,50,51,52,53,54,55,56,57^ In total, 39 251 records were found through literature searching, and 40 studies^14,15,16,17,18,19,24,25,26,27,28,29,30,31,32,33,34,35,36,37,38,39,40,41,42,43,44,45,46,47,48,49,50,51,52,53,54,55,56,57^ were included after being screened by the researchers. Refer to the eResults in Supplement 1 for the detailed retrieval steps.

### Study Characteristics

Table 1 shows the characteristics of all studies.^14,15,16,17,18,19,24,25,26,27,28,29,30,31,32,33,34,35,36,37,38,39,40,41,42,43,44,45,46,47,48,49,50,51,52,53,54,55,56,57^ The 40 included articles were published between 1988 and 2021, and the data for the included studies were collected between 1984 and 2017 (5 studies^19,24,25,30,38^ did not specify when the data were collected). A total of 310 584 participants were included, with boys accounting for 39% (sex was not reported in 10 studies^16,17,24,25,26,32,37,40,44,51^). Seventeen studies reported treatment rates for any mental disorder,^14,15,16,17,24,26,29,32,34,37,38,41,44,47,51,54,55^ 20 for depressive disorders,^14,17,18,19,26,30,31,33,35,36,39,42,45,46,47,52,53,55,56,57^ 9 for anxiety disorders,^14,17,26,30,35,47,48,49,55^ 10 for ADHD,^14,25,27,28,30,31,40,43,47,50^ and 7 for behavior disorders.^26,31,37,40,47,55,56^

**Table 1.** Study Characteristics

Source and country	WHO region	Income	Year of data collection	Sample origin	Sample size, No.	Age, mean (SD) or range, y, or grade	Male, %	Timeframe	Informant source	Outcomes	Tools
Angold et al,^24^ 2002; US	AMR	HIC	NR	School	920	9-17	NR	Last 3 mo	Parent and child	AMD	CAPA
Bird et al,^25^ 2008; US	AMR	HIC	NR	Community	2491	5-13	NR	Last year	Child	ADHD	DISC-IV
Borges et al,^17^ 2008; Mexico	AMR	UMIC	2005	Community	3005	12-17	NR	Last year	Child	AMD, DD, and AD	CIDI
Buckner and Bassuk,^26^ 1997; US	AMR	HIC	1992-1995	Health care institution	94	9-17	NR	Last 6 mo or lifetime	Parent	DD, AD, AMD, and BD	DISC 2.3
Bussing et al,^27^ 2003; US	AMR	HIC	1998-2000	School	1615	7.8 (1.8)	52	Current	Parent	ADHD	DISC-IV
Bussing et al,^28^ 2011; US	AMR	HIC	2004-2005	School	168	6.8 (0.5)	47	Last year or lifetime	Parent	ADHD	DISC-IV
Canino et al,^29^ 2004; US	AMR	HIC	1999-2000	Community	1879	4-17	53	Last year	Parent and child	AMD	DISC-IV
Chavira et al,^30^ 2004; US	AMR	HIC	NR	Health care institution	190	8-17	50	Lifetime	Parent	AD, DD, and ADHD	ADIS-C/P
Coker et al,^31^ 2009; US	AMR	HIC	2004-2006	School	5121	Fifth grade	51	Lifetime	Parent	ADHD, BD, and DD	DISC
Cuffe et al,^32^ 2001; US	AMR	HIC	1987-1989	School	579	12.83	NR	Lifetime	Parent and child	AMD	K-SADS
Cummings and Druss,^33^ 2011; US	AMR	HIC	2004-2008	Community	90 855	15.1 (0.02); 12-17	28	Last year	Child	DD	CIDI-SF
Daeem et al,^34^ 2019; Israel	EMR	HIC	2012-2013	School	1639	Ninth grade	48	Lifetime	Parent	AMD	DAWBA
Essau et al,^35^ 2005; Germany	EMR	HIC	1996-1997	School	1035	12-17	41	Lifetime	Child	AD and DD	CIDI
Fatori et al,^16^ 2019; Brazil	AMR	UMIC	2010-2011	Community	2511	6-12	NR	Lifetime	Parent	AMD	DAWBA
Flament et al,^36^ 2001; France	EUR	HIC	1988-1990	School	3287	17.6 (1.6)	47	Lifetime	Child	DD	K-SADS
Georgiades et al,^37^ 2019; Canada	AMR	HIC	2014-2015	Community	6537	4-17	NR	Last 6 mo	Parent and child	AMD and BD	MINI-KID
Gómez-Beneyto et al,^38^ 1994; Spain	EUR	HIC	NR	Community	1127	8-15	50	Lifetime	Child	AMD	K-SADS
Gould et al,^39^ 2009; US	AMR	HIC	2002-2006	School	2342	15 (1.0); 13-19	42	Last year	Child	DD	DISC-IV
Heiervang et al,^40^ 2007; Norway	EUR	HIC	2002-2003	School	6297	7-9	NR	Lifetime	Parent	ADHD and BD	DAWBA
Leaf et al,^41^ 1996; US	AMR	HIC	1991-1992	NR	1285	12.9 (2.6); 9-17	53	Last year or lifetime	Parent and child	AMD	DISC 2.3
Lewinsohn et al,^42^ 1998; US	AMR	HIC	1987-1989	School	1507	13.4	52	Lifetime	Child	DD	K-SADS
Locke et al,^43^ 2017; US	AMR	HIC	2008-2009	Health care institutions	23 601	5-17	63	Lifetime	Child	ADHD	*ICD-9*
Maalouf et al,^44^ 2016; Lebanon	EMR	LMIC	2012	Community	510	11-17	NR	Lifetime	Parent	AMD	DAWBA
Magklara et al,^45^ 2015; Greece	EUR	HIC	2007-2008	School	2427	16-18	41	Last year	Child	DD	*ICD-10*
Meredith et al,^46^ 2009; US	AMR	HIC	2005-2006	Health care institution	368	15.2 (1.4); 13-17	22	Last 6 mo	Child	DD	DISC
Merikangas et al,^14^ 2010; US	AMR	HIC	2001-2004	Community	3042	8-15	49	Last year	Child	AMD, ADHD, AD, and DD	DISC-IV
Merikangas et al,^47^ 2011; US	AMR	HIC	2002-2004	Community	6483	13-18	51	Lifetime	Parent and child	AMD, DD, AD, ADHD, and BD	CIDI
Niermann et al,^48^ 2021; Germany	EUR	HIC	2015	Community	635	14-17	42	Lifetime	Child	AD	CIDI
Reardon et al,^49^ 2020; Great Britain	EUR	HIC	2016-2017	School	222	9.6 (1.2); 7-11	52	Lifetime	parent	AD	ADIS-P
Sawyer et al,^50^ 2004; Australia	AMR	HIC	1998	Community	398	6-17	69	Last 6 mo	Parent	ADHD	DISC-IV
Sawyer et al,^51^ 2018; Australia	AMR	HIC	2013-2014	Community	6310	4-17	NR	Last year	Parent	AMD	DISC-IV
Stiffman et al,^52^ 1988; US	AMR	HIC	1984-1985	Health care institution	2787	13-18	33	Last year	Child	DD	DICA
Tanielian et al,^53^ 2009; US	AMR	HIC	2005-2006	Health care institution	4713	15.2 (1.3); 13-17	22	Last 6 mo	Parent and child	DD	DISC
Vicente et al,^54^ 2012; Chile	AMR	HIC	2007-2009	Community	1558	4-18	51	Last year	Parent and child	AMD	DISC-IV
Wagner et al,^55^ 2017; Austria	EUR	HIC	2013-2015	School	3477	10-18	45	Lifetime	Child	DD, AD, BD, and AMD	CDI-MD
Wu et al,^56^ 1999; US	AMR	HIC	1991-1992	Community	1285	12.9 (2.6); 9-17	53	Last year or lifetime	Parent and child	DD and BD	DISC 2.3
Wu et al,^19^ 2001; US	AMR	HIC	NR	Community	206	9-17	44	Last year	Parent and child	DD	DISC 2.3
Zhang et al,^57^ 2021; US	AMR	HIC	2010-2016	Community	11 4250	12-17	51	Last year	Child	DD	CIDI-SF
Zhong et al,^18^ 2013; China	WPR	UMIC	2009-2010	Community	3582	6-14	52	Lifetime	Parent and child	DD	MINI-KID
Zwaanswijk et al,^15^ 2005; Netherlands	EUR	HIC	2000-2002	Health care institution	246	4-11	56	Last year	Parent	AMD	DISC-IV

Abbreviations: AD, anxiety disorders; ADHD, attention-deficit/hyperactivity disorder; ADIS-C/P, Anxiety Disorders Interview, Children-Parent Version; ADIS-P, Anxiety Disorder Interview Schedule, Parent Version; AMD, any mental disorder; AMR, region of the Americas; BD, behavior disorders; CAPA, Child and Adolescent Psychiatric Assessment; CDI-MD, Children’s Diagnostic Interview for Mental Disorders; CIDI, World Mental Health Composite International Diagnostic Interview; CIDI-SF, World Composite International Diagnostic Interview–Short Form; DAWBA, Development and Well-Being Assessment; DICA, Diagnostic Interview for Children and Adolescents; DISC, Diagnostic Interview for Children; DISC-IV, Diagnostic Interview for Children, Version IV; DISC 2.3, NIMH Diagnostic Interview Schedule for Children, Version 2.3; DD, depressive disorders; EMR, Eastern Mediterranean region; EUR, European region; HIC, high-income country; *ICD-9*, *International Classification of Diseases, Ninth Revision*; *ICD-10*, *International Statistical Classification of Diseases and Related Health Problems, Tenth Revision*; K-SADS, Present Episode Version of the Schedule for Affective Disorders and Schizophrenia for School-Aged Children; LMIC, lower-middle income country; MINI-KID, Mini International Neuropsychiatric Interview for Children and Adolescents; NR, information not reported in the article; UMIC, upper-middle income country; WHO, World Health Organization; WPR, Western Pacific region.

### Quality Assessment

For the risk of bias assessment, 34 (85%) included articles were of medium quality (JBI score 5-7) and 6 (15%) articles were of high quality (JBI score ≥8). The details of the quality assessment are shown in the eTable in Supplement 1.

### Treatment Rates for Mental Disorders

**Figure 2.**
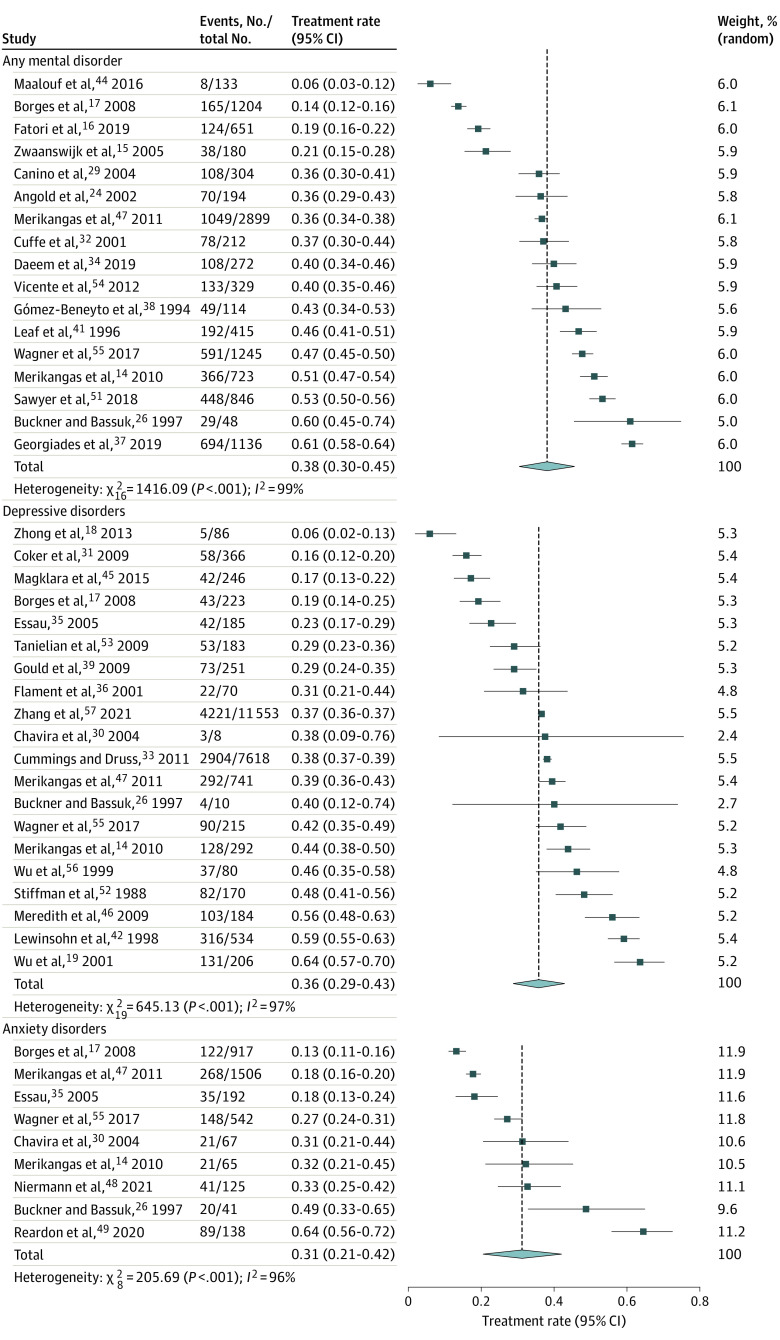
Forest Plots for Treatment Rates of Mental Disorders in Children and Adolescents

The pooled treatment rate of any mental disorder from the random-effects meta-analysis was 38% (95% CI, 30%-45%) (Figure 2), which was highly heterogeneous (*I*^2^ = 99%; *P* < .001). The combined treatment rate was 36% (95% CI, 29%-43%; *I^2^* = 97%; *P* < .001) (Figure 2) for depressive disorders, 31% (95% CI, 21%-42%; *I^2^* = 96%; *P* < .001) (Figure 2) for anxiety disorders, 58% (95% CI, 42%-73%; *I^2^* = 100%; *P* < .001) (eFigure 52 in Supplement 1) for ADHD, and 49% (95% CI, 35%-64%; *I^2^* = 99%; *P* < .001) (eFigure 53 in Supplement 1) for behavior disorders, all of which were highly heterogeneous.

### Subgroup Analysis

#### Treatment Rates by Year of Data Collection

**Figure 3.**
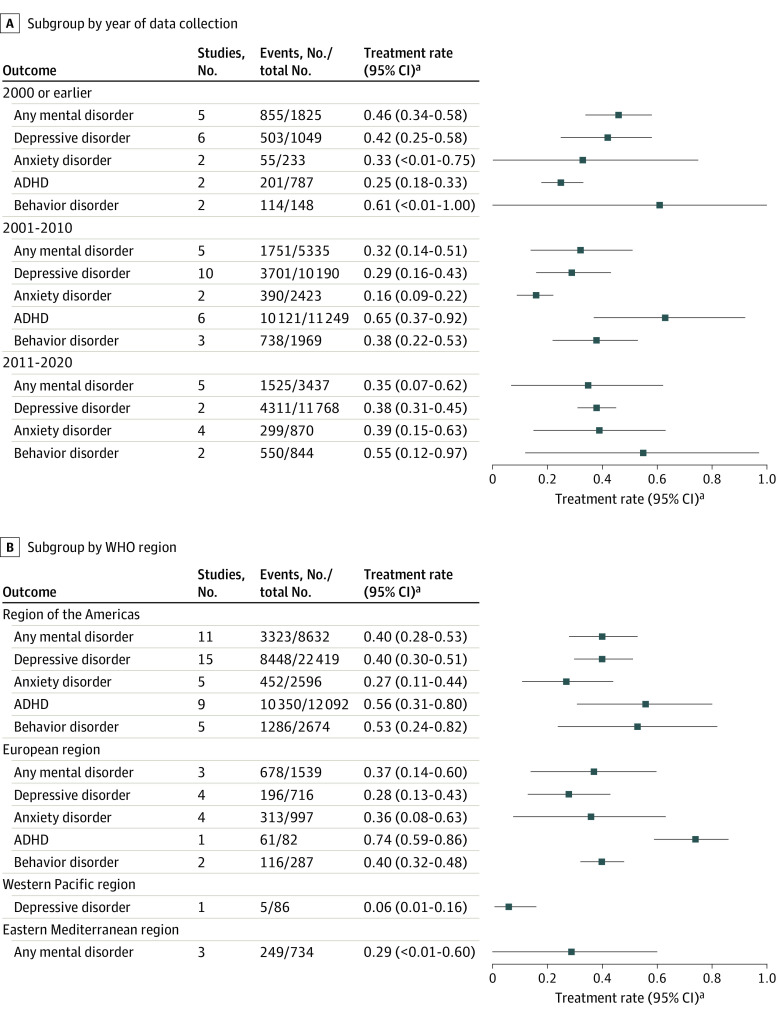
Forest Plots for Subgroup Analyses by Different Factors A, Subgroup by year of data collection. B, Subgroup by World Health Organization (WHO) region. ADHD indicates attention-deficit/hyperactivity disorder. ^a^95% CIs adjusted by Bonferroni correction.

Figure 3A shows treatment rates by year of data collection. There were no significant differences in treatment rates for any mental disorder, depressive disorders, anxiety disorders, or behavior disorders (eFigures 1-3, and 5 in Supplement 1). Subgroup differences were statistically significant only for ADHD (eFigure 4 in Supplement 1).

#### Treatment Rates by Region

Figure 3B shows treatment rates by region. For any mental disorder, anxiety disorders, ADHD, and behavior disorders, treatment rates were not statistically different among all regions (eFigures 6 and 8-10 in Supplement 1). For depressive disorders, treatment rates were higher in the Americas (40%; 95% CI, 30%-51%) than in Europe (28%; 95% CI, 13%-43%) and the Western Pacific region (6%; 95% CI, 1%-16%) (*P* < .001; eFigure 7 in Supplement 1).

#### Treatment Rates by Age

eFigure 54 in Supplement 1 shows treatment rates by age. There were no significant differences between children (≤12 years of age) and adolescents (13-17 years of age) in treatment rates of any mental disorder, ADHD, and behavior disorders (eFigures 11, 14, and 15 in Supplement 1). The treatment rate for depressive disorders was higher among adolescents (36%; 95% CI, 25%-46%) than children (11%; 95% CI, 0%-25%) (*P* < .001; eFigure 12 in Supplement 1), whereas the treatment rate for anxiety disorders was higher among children (64%; 95% CI, 52%-75%) than adolescents (20%; 95% CI, 9%-30%) (*P* < .001; eFigure 13 in Supplement 1).

#### Treatment Rates by Income Level

eFigure 55 in Supplement 1 shows treatment rates by income level. The treatment rate for any mental disorder in lower-middle income countries was 6% (95% CI, 2%-14%), in upper-middle income countries was 24% (95% CI, 2%-47%), and in high-income countries (HICs) was 43% (95% CI, 35%-52%). The treatment rate for depressive disorders was 13% (95% CI, 0%-31%) in UMICs and 39% (95% CI, 29%-48%) in HICs (eFigure 17 in Supplement 1); the treatment rate for anxiety disorders was 31% (95% CI, 16%-46%) in UMICs and 34% (95% CI, 18%-49%) in HICs (eFigure 18 in Supplement 1). There were significant differences among income groups in the treatment rates of any mental disorder, depressive disorders, and anxiety disorders (eFigures 16-18 in Supplement 1). Studies reporting ADHD and behavior disorders were all from HICs.

#### Treatment Rates by Other Important Characteristics

Subgroup analyses were also performed using other important characteristics, such as timeframe of diagnosis, informant source, health service type, sample source population, and internalizing or externalizing disorder (eFigures 19-43 in Supplement 1). Incongruity was observed in treatment rates of anxiety reported by different informant sources: parents reported higher treatment rates (48% [95% CI, 21%-76%]) than children themselves (24% [95% CI, 13%-35%]) (eFigure 28 in Supplement 1). The combined treatment rate for internalizing disorders was 34% (95% CI, 26%-43%), significantly lower than the rate for externalizing disorders (54%; 95% CI, 39%-70%) (eFigure 43 in Supplement 1).

### Sensitivity Analysis and Publication Bias

Leave-one-out sensitivity analysis of any mental disorder, depressive disorders, anxiety disorders, ADHD, and behavior disorders showed acceptable stability of the pooled results (Table 2; eFigures 44-48 in Supplement 1). Outlier analysis was performed by excluding studies in which the 95% CIs were outside the aggregated 95% CI of all studies, and the combined results showed insignificant change (eFigure 49 in Supplement 1). Another round of sensitivity analysis, including only studies (n = 32) with the upper limit of participant age range of 18 years, showed that the combined estimates were comparable to those based on all 40 included studies (eFigure 50 in Supplement 1). Funnel plots, together with the Egger test and the trim-and-fill method suggested significant publication bias for anxiety disorders and ADHD (eFigure 51 in Supplement 1).

**Table 2.** Summary of Leave-One-Out Sensitivity Analysis Results

Outcome	Combined treatment rate (95% CI)		
	Minimum	Maximum	Overall
Any mental disorder	0.36 (0.29-0.44)	0.40 (0.33-0.46)	0.38 (0.30-0.45)
Depressive disorders	0.34 (0.28-0.41)	0.37 (0.31-0.44)	0.36 (0.29-0.43)
Anxiety disorders	0.26 (0.19-0.33)	0.34 (0.23-0.45)	0.31 (0.21-0.42)
ADHD	0.52 (0.38-0.65)	0.60 (0.46-0.75)	0.58 (0.42-0.73)
Behavior disorders	0.44 (0.32-0.56)	0.53 (0.38-0.69)	0.49 (0.35-0.64)

Abbreviation: ADHD, attention-deficit/hyperactivity disorder.

## Discussion

This meta-analysis included 40 studies, comprising 310 584 children and adolescents, that reported treatment rates for mental disorders among children and adolescents. After synthesis, the combined treatment rate was 38% for any mental disorder, 36% for depressive disorders, 31% for anxiety disorders, 58% for ADHD, and 49% for behavior disorders. Moreover, treatment rates for mental disorders among children and adolescents varied significantly across different regions and income levels. Other characteristics, such as age, also showed a nonnegligible association with treatment rates. Our findings provide important evidence for constructing evidence-based, targeted intervention policies and measures that aim to improve treatment rates for mental disorders among children and adolescents.

The generally low treatment rates for children and adolescents with mental disorders could be associated with multiple determinants. Despite the consensus on the more hazardous effect of mental disorders on youths, compared with adults, the coverage of mental health services is inadequate.^54,58^ Fewer approved drug therapies for treating mental disorders among children and adolescents limit possible treatment options.^59^ Moreover, lack of knowledge about mental health and available help, perceived social stigma and embarrassment, therapeutic relationships with professionals, financial costs associated with mental health services, and logistical concerns were also frequently cited barriers.^60^ Compared with ADHD and behavior disorders, even lower treatment rates were found for depressive disorders and anxiety disorders, probably because children and adolescents are reluctant to disclose their emotional difficulties to other people for support, owing to fears of negative social consequences.^61^ Low public acceptance of the use of antidepressants and cognitive behavioral therapy for young people may also hinder their self-rescue behaviors.^62^ In addition, parents may perceive anxiety or depression in children and adolescents as typical for their age group, and think that it can be improved without professional help.^49^

Income levels are closely associated with medical investment, which will undoubtedly influence treatment rates of mental disorders to a certain extent, as demonstrated by the pooled estimates of our study. For example, the treatment rate for any mental disorder was 24% in UMICs and 43% in HICs, the treatment rate for depressive disorders was 13% in UMICs and 39% in HICs, and the treatment rate for anxiety disorders was 31% in UMICs and 34% in HICs. Compared with HICs, mental health services are severely underfunded in LMICs: according to the World Health Organization 2020 Mental Health Atlas, government annual mental health expenditure per capita was $52.73 in HICs, compared with $3.29 in UMICs and $0.08 in low-income countries.^63^ Moreover, for LMICs, there is an overreliance on out-of-pocket payments to support mental health, which further obstructs development of health care systems.^64^ In addition, the mental health workforce is very underrepresented.^63^ Therefore, at the governmental level, the utmost priority for LMICs is to increase allocation of mental health expenditures.^65^ Public education to increase knowledge of mental disorders is also vital to foster mental health service–seeking behaviors.^66^

This study also found that, even for regions with the same income level, significant differences in treatment rates were observed. A previous study showed marked disparities in the use of child mental health services across developed countries.^67^ The studies from Europe and the Americas that were included in this meta-analysis were largely from HICs, but we found that the treatment rate for depressive disorders in the Americas was 40%, significantly higher than the 28% in Europe. This discrepancy may be caused by social norms and the stigma associated with psychological disorders, for negative biases against people with mental illnesses have been found to be common in Western European countries^68^; compared with US adults (23%), a much higher proportion of European adults (41%-45%) reported unwillingness to see a professional when experiencing emotional distress.^69^ These findings accentuate the necessity of incorporating cultural factors when devising domestic intervention policies for improving treatment rates of mental disorders among children and adolescents in different countries.

Although treatment rates for any mental disorder, ADHD, and behavior disorders were comparable between children and adolescents in this study, age was associated with treatment rates of depressive and anxiety disorders. Specifically, compared with children, adolescents were more than 3 times as likely to be treated for diagnosed depressive disorders (36% vs 11%). The onset of childhood depression can be subtle: children may express irritability and frustration through tantrums and behavioral problems rather than verbally expressing their feelings.^70^ Meanwhile, compared with adolescents, children with a diagnosis of depressive disorder are less prone to exhibit suicidal behavior,^70^ which may account for their lower treatment rates. Only 2 original studies regarding childhood depressive disorders satisfied the inclusion criteria of the current meta-analysis, which raises concerns about the representativeness of treatment rates for this age group. The treatment rate of anxiety disorders among adolescents was lower than among children, probably because, compared with adolescents, children with anxiety disorders are more prone to experiencing physical symptoms such as shortness of breath, headaches, stomach pain, and heart palpitations,^71^ which may prompt parents to seek assistance.

### Limitations

This meta-analysis provides a comprehensive and systematic summary of treatment rates for mental disorders in children and adolescents. Nevertheless, some limitations should be noted. First, owing to the limited number of published studies, we were able to combine treatment rates only for several of the most prevalent mental disorders among children and adolescents; other less commonly seen but equally important psychiatric problems in this age group, such as bipolar disorder, borderline personality disorder, and autism spectrum disorder, could not be investigated. Second, as a high level of heterogeneity has been discerned among the included studies, although we performed elaborate subgroup analyses to investigate potential sources of heterogeneity, because we were restricted by the design of the original studies, other meaningful sources could not be analyzed. Third, a small number of the included studies in some subgroups may compromise the representativeness and statistical power of the pooled estimates; therefore, the generalization of the study results should be pursued with caution. Future studies with large and representative samples, focusing on less commonly seen mental disorders, and providing more exhaustive analytical results, are needed, especially from less-developed countries or regions, to better understand the treatment rates of psychiatric problems among young people.

## Conclusions

In this systematic review and meta-analysis of high-quality evidence, we found that, in general, treatment rates for mental disorders among children and adolescents are unsatisfactorily low, especially for depression and anxiety. Individual characteristics such as age and environmental factors such as region and income level were associated with treatment rates of mental disorders among this age group. Targeted intervention policies and measures should be designed and implemented to improve treatment rates of psychiatric disorders among youths. Some promising methods have already been highlighted in newly published evidence. For instance, telemedicine was found to be effective in reducing treatment barriers for psychiatric patients, and skills training in mental health assessment and treatment for primary care professionals could also be considered.^72^

## Notes

Supplement 1. **eTable.** JBI Quality Scores of Included Studies (N=40) **eFigure 1.** Treatment Rates for Any Mental Disorder by Year of Data Collection **eFigure 2.** Treatment Rates for Depressive Disorders by Year of Data Collection **eFigure 3.** Treatment Rates for Anxiety Disorders by Year of Data Collection **eFigure 4.** Treatment Rates for ADHD by Year of Data Collection **eFigure 5.** Treatment Rates for Behavior Disorders by Year of Data Collection **eFigure 6.** Treatment Rates for Any Mental Disorder by Region **eFigure 7.** Treatment Rates for Depressive Disorders by Regions **eFigure 8.** Treatment Rates for Anxiety Disorders by Regions **eFigure 9.** Treatment Rates for ADHD by Regions **eFigure 10.** Treatment Rates for Behavior Disorders by Regions **eFigure 11.** Treatment Rates for Any Mental Disorder by Age Groups **eFigure 12.** Treatment Rates for Depressive Disorders by Age Groups **eFigure 13.** Treatment Rates for Anxiety Disorders by Age Groups **eFigure 14.** Treatment Rates for ADHD by Age Groups **eFigure 15.** Treatment Rates for Behavior Disorders by Age Groups **eFigure 16.** Treatment Rates for Any Mental Disorder by Income Groups **eFigure 17.** Treatment Rates for Depressive Disorders by Income Groups **eFigure 18.** Treatment Rates for Anxiety Disorders by Income Groups **eFigure 19.** Subgroup by Timeframe **eFigure 20.** Treatment Rates for Any Mental Disorder by Timeframes **eFigure 21.** Treatment Rates for Depressive Disorders by Timeframes **eFigure 22.** Treatment Rates for Anxiety Disorders by Timeframes **eFigure 23.** Treatment Rates for ADHD by Timeframes **eFigure 24.** Treatment Rates for Behavior Disorders by Timeframes **eFigure 25.** Subgroup by Informant Source **eFigure 26.** Treatment Rates for Any Mental Disorder by Informant Sources **eFigure 27.** Treatment Rates for Depressive Disorders by Informant Sources **eFigure 28.** Treatment Rates for Anxiety Disorders by Informant Sources **eFigure 29.** Treatment Rates for ADHD by Informant Sources **eFigure 30.** Treatment Rates for Behavior Disorders by Informant Sources **eFigure 31.** Subgroup by Health Service Type **eFigure 32.** Treatment Rates for Any Mental Disorder by Health Service Types **eFigure 33.** Treatment Rates for Depressive Disorders by Health Service Types **eFigure 34.** Treatment Rates for Anxiety Disorders by Health Service Types **eFigure 35.** Treatment Rates for ADHD by Health Service Types **eFigure 36.** Treatment Rates for Behavior Disorders by Health Service Types **eFigure 37.** Subgroup by Sample Origin Population **eFigure 38.** Treatment Rates for Any Mental Disorder by the Sample Origin Population **eFigure 39.** Treatment Rates for Depressive Disorders by the Sample Origin Population **eFigure 40.** Treatment Rates for Anxiety Disorders by the Sample Origin Population **eFigure 41.** Treatment Rates for ADHD by the Sample Origin Population **eFigure 42.** Treatment Rates for Behavior Disorders by the Sample Origin Population **eFigure 43.** Treatment Rates for Internalizing and Externalizing Disorders **eFigure 44.** Sensitivity Analysis Forest Plots for Any Mental Disorder **eFigure 45.** Sensitivity Analysis Forest Plots for Depressive Disorders **eFigure 46.** Sensitivity Analysis Forest Plots for Anxiety Disorders **eFigure 47.** Sensitivity Analysis Forest Plots for ADHD **eFigure 48.** Sensitivity Analysis Forest Plots for Behavior Disorders **eFigure 49.** Sensitivity Analysis by Excluding Studies in Which the 95% CI Was Outside the Aggregated 95% CI of All Studies **eFigure 50.** Sensitivity Analysis by Excluding Studies Not Reported Age Ranges **eFigure 51.** Funnel Plot (Left) and Trim-and-Fill Funnel Plot (Right) **eFigure 52.** Treatment Rates for ADHD **eFigure 53.** Treatment Rates for Behavior Disorders **eFigure 54.** Subgroup Analysis by Age Group **eFigure 55.** Subgroup Analysis by Income Level **eMethods 1.** Retrieval Strategy **eMethods 2.** Exposure **eResults.** Study Selection **eAppendix.** Explicit Search Entry
eReferences.

Supplement 2.
Data Sharing Statement
