# Supplementary material for: Treatment Rates for Mental Disorders Among Children and Adolescents: A Systematic Review and Meta-Analysis
Source: JAMA Netw Open. 2023 Oct 18;6(10):e2338174. doi: 10.1001/jamanetworkopen.2023.38174 (PMC10585417; doi:10.1001/jamanetworkopen.2023.38174)
Supplement: Supplement 1. — eTable. JBI Quality Scores of Included Studies (N=40) eFigure 1. Treatment Rates for Any Mental Disorder by Year of Data Collection eFigure 2. Treatment Rates for Depressive Disorders by Year of Data Collection eFigure 3. Treatment Rates for Anxiety Disorders by Year of Data Collection eFigure 4. Treatment Rates for ADHD by Year of Data Collection eFigure 5. Treatment Rates for Behavior Disorders by Year of Data Collection eFigure 6. Treatment Rates for Any Mental Disorder by Region eFigure 7. Treatment Rates for Depressive Disorders by Regions eFigure 8. Treatment Rates for Anxiety Disorders by Regions eFigure 9. Treatment Rates for ADHD by Regions eFigure 10. Treatment Rates for Behavior Disorders by Regions eFigure 11. Treatment Rates for Any Mental Disorder by Age Groups eFigure 12. Treatment Rates for Depressive Disorders by Age Groups eFigure 13. Treatment Rates for Anxiety Disorders by Age Groups eFigure 14. Treatment Rates for ADHD by Age Groups eFigure 15. Treatment Rates for Behavior Disorders by Age Groups eFigure 16. Treatment Rates for Any Mental Disorder by Income Groups eFigure 17. Treatment Rates for Depressive Disorders by Income Groups eFigure 18. Treatment Rates for Anxiety Disorders by Income Groups eFigure 19. Subgroup by Timeframe eFigure 20. Treatment Rates for Any Mental Disorder by Timeframes eFigure 21. Treatment Rates for Depressive Disorders by Timeframes eFigure 22. Treatment Rates for Anxiety Disorders by Timeframes eFigure 23. Treatment Rates for ADHD by Timeframes eFigure 24. Treatment Rates for Behavior Disorders by Timeframes eFigure 25. Subgroup by Informant Source eFigure 26. Treatment Rates for Any Mental Disorder by Informant Sources eFigure 27. Treatment Rates for Depressive Disorders by Informant Sources eFigure 28. Treatment Rates for Anxiety Disorders by Informant Sources eFigure 29. Treatment Rates for ADHD by Informant Sources eFigure 30. Treatment Rates for Behavior Disorders by Informant Sources eFigure 31. Subgroup by H [file jamanetwopen-e2338174-s001.pdf]

Supplementary Online Content

Wang S, Li Q, Lu J, et al. Treatment rates for mental disorders among children and adolescents: a systematic review and meta-analysis. *JAMA Netw Open.* 2023;6(10):e2338174. doi:10.1001/jamanetworkopen.2023.38174

eTable. JBI Quality Scores of Included Studies (N=40)
eFigure 1. Treatment Rates for Any Mental Disorder by Year of Data Collection
eFigure 2. Treatment Rates for Depressive Disorders by Year of Data Collection
eFigure 3. Treatment Rates for Anxiety Disorders by Year of Data Collection
eFigure 4. Treatment Rates for ADHD by Year of Data Collection
eFigure 5. Treatment Rates for Behavior Disorders by Year of Data Collection
eFigure 6. Treatment Rates for Any Mental Disorder by Regions
eFigure 7. Treatment Rates for Depressive Disorders by Regions
eFigure 8. Treatment Rates for Anxiety Disorders by Regions
eFigure 9. Treatment Rates for ADHD by Regions
eFigure 10. Treatment Rates for Behavior Disorders by Regions
eFigure 11. Treatment Rates for Any Mental Disorder by Age Groups
eFigure 12. Treatment Rates for Depressive Disorders by Age Groups
eFigure 13. Treatment Rates for Anxiety Disorders by Age Groups
eFigure 14. Treatment Rates for ADHD by Age Groups
eFigure 15. Treatment Rates for Behavior Disorders by Age Groups
eFigure 16. Treatment Rates for Any Mental Disorder by Income Groups
eFigure 17. Treatment Rates for Depressive Disorders by Income Groups
eFigure 18. Treatment Rates for Anxiety Disorders by Income Groups
eFigure 19. Subgroup by Timeframe
eFigure 20. Treatment Rates for Any Mental Disorder by Timeframes
eFigure 21. Treatment Rates for Depressive Disorders by Timeframes
eFigure 22. Treatment Rates for Anxiety Disorders by Timeframes
eFigure 23. Treatment Rates for ADHD by Timeframes
eFigure 24. Treatment Rates for Behavior Disorders by Timeframes
eFigure 25. Subgroup by Informant Source
eFigure 26. Treatment Rates for Any Mental Disorder by Informant Sources
eFigure 27. Treatment Rates for Depressive Disorders by Informant Sources
eFigure 28. Treatment Rates for Anxiety Disorders by Informant Sources
eFigure 29. Treatment Rates for ADHD by Informant Sources
eFigure 30. Treatment Rates for Behavior Disorders by Informant Sources
eFigure 31. Subgroup by Health Service Type
eFigure 32. Treatment Rates for Any Mental Disorder by Health Service Types
eFigure 33. Treatment Rates for Depressive Disorders by Health Service Types
eFigure 34. Treatment Rates for Anxiety Disorders by Health Service Types
eFigure 35. Treatment Rates for ADHD by Health Service Types
eFigure 36. Treatment Rates for Behavior Disorders by Health Service Types
eFigure 37. Subgroup by Sample Origin Population

© 2023 Wang S et al. *JAMA Network Open.*

eFigure 38. Treatment Rates for Any Mental Disorder by the Sample Origin Population
eFigure 39. Treatment Rates for Depressive Disorders by the Sample Origin Population
eFigure 40. Treatment Rates for Anxiety Disorders by the Sample Origin Population
eFigure 41. Treatment Rates for ADHD by the Sample Origin Population
eFigure 42. Treatment Rates for Behavior Disorders by the Sample Origin Population
eFigure 43. Treatment Rates for Internalizing and Externalizing Disorders
eFigure 44. Sensitivity Analysis Forest Plots for Any Mental Disorder
eFigure 45. Sensitivity Analysis Forest Plots for Depressive Disorders
eFigure 46. Sensitivity Analysis Forest Plots for Anxiety Disorders
eFigure 47. Sensitivity Analysis Forest Plots for ADHD
eFigure 48. Sensitivity Analysis Forest Plots for Behavior Disorders
eFigure 49. Sensitivity Analysis by Excluding Studies in Which the 95% CI Was Outside the Aggregated 95% CI of All Studies
eFigure 50. Sensitivity Analysis by Excluding Studies Not Reported Age Ranges
eFigure 51. Funnel Plot (Left) and Trim-and-Fill Funnel Plot (Right)
eFigure 52. Treatment Rates for ADHD
eFigure 53. Treatment Rates for Behavior Disorders
eFigure 54. Subgroup Analysis by Age Group
eFigure 55. Subgroup Analysis by Income Level
eMethods 1. Retrieval Strategy
eMethods 2. Exposure
eResults. Study Selection
eAppendix. Explicit Search Entry
eReferences.

This supplementary material has been provided by the authors to give readers additional information about their work.

eTable. JBI Quality Scores of Included Studies (N=40)

Study	Item 1	Item 2	Item 3	Item 4	Item 5	Item 6	Item 7	Item 8	Item 9	Total
Angold et al, 2002	Yes	Yes	Yes	Yes	Yes	Yes	No	Yes	No	7
Bird et al, 2008	Yes	Yes	Yes	No	Unclear	Yes	No	Yes	Unclear	5
Borges et al, 2008	Yes	Yes	Yes	No	No	Yes	Unclear	Yes	No	5
Buckner et al, 1997	No	Unclear	No	Yes	Yes	Yes	Yes	Yes	Yes	6
Bussing et al, 2003	Yes	Yes	Yes	Yes	Yes	Yes	No	Yes	No	7
Bussing et al, 2011	Yes	Yes	Yes	Yes	Yes	Yes	No	Yes	No	7
Canino et al, 2004	Yes	Yes	Yes	No	Yes	Yes	No	Yes	Yes	7
Chavira et al, 2004	No	Yes	No	Yes	Yes	Yes	Yes	Yes	No	6
Coker et al, 2009	Yes	Yes	Yes	Yes	Yes	Yes	No	Yes	No	7
Cuffe et al, 2001	Yes	Unclear	Yes	Yes	Yes	Yes	Yes	Yes	Yes	8
Cummings et al, 2011	Yes	Unclear	Yes	No	Yes	Yes	No	Yes	Unclear	5
Daeem et al, 2019	No	No	Yes	Yes	Yes	Yes	Yes	Yes	Yes	7
Essau et al, 2005	No	Yes	Yes	Yes	Unclear	Yes	No	Yes	No	5
Fatori et al, 2019	Yes	Unclear	Yes	Yes	No	Yes	Yes	Yes	Unclear	6
Flament et al, 2001	Yes	Yes	No	No	Yes	Yes	Yes	Yes	Yes	7
Georgiades et al, 2019	Yes	Yes	Yes	Yes	No	Yes	Yes	Yes	No	7
Gómez-Beneyto et al, 1994	Yes	Yes	Yes	No	Yes	Yes	Yes	Yes	Yes	8
Gould et al, 2009	Yes	Unclear	Yes	Yes	Yes	Yes	Yes	Yes	No	7
Heiervang et al, 2007	Yes	Unclear	Yes	No	No	Yes	Yes	Yes	No	5
Leaf et al, 1996	Yes	No	Yes	Yes	Unclear	Yes	Yes	Yes	Unclear	6
Lewinsohn et al, 1998	Yes	Yes	Yes	No	Yes	Yes	No	Yes	No	6
Locke et al, 2017	No	No	Yes	Yes	Yes	Yes	No	Yes	No	5
Maalouf et al, 2016	Yes	Yes	Yes	Yes	No	Yes	Yes	Yes	No	7
Magklara et al, 2015	Yes	Yes	Yes	Yes	Yes	Yes	Yes	Yes	Yes	9
Meredith et al, 2009	No	No	Yes	Yes	Yes	Yes	No	Yes	Yes	6
Merikangas et al, 2010	Yes	Yes	Yes	No	No	Yes	No	Yes	No	5
Merikangas et al, 2011	Yes	No	Yes	Yes	Yes	Yes	Yes	Yes	Yes	8
Niermann et al, 2021	Yes	Yes	Yes	Yes	No	Yes	Yes	Yes	No	7
Reardon et al, 2020	Yes	Yes	Yes	Yes	No	Yes	No	Yes	Unclear	6
Sawyer et al, 2004	Yes	Yes	Yes	Yes	No	Yes	No	Yes	No	6
Sawyer et al, 2018	Yes	Unclear	Yes	Yes	No	Yes	No	Yes	No	5
Stiffman et al, 1988	Yes	Unclear	Yes	Yes	Yes	Yes	No	Yes	Yes	7
Tanielian et al, 2009	No	No	Yes	Yes	Yes	Yes	No	Yes	Unclear	5
Vicente et al, 2012	Yes	Yes	Yes	No	Yes	Yes	Yes	Yes	Yes	8
Wagner et al, 2017	Yes	No	Yes	Yes	Yes	Yes	No	Yes	No	6
Wu et al, 1999	Yes	Yes	Yes	Yes	Unclear	Yes	No	Yes	Unclear	6
Wu et al, 2001	Yes	Unclear	Yes	Yes	Unclear	Yes	Yes	Yes	Unclear	6
Zhang et al, 2021	Yes	Yes	Yes	Yes	Yes	Yes	No	Yes	Unclear	7
Zhong et al, 2013	Yes	Yes	Yes	Yes	Yes	Yes	Yes	Yes	No	8
Zwaanswijk et al, 2005	No	Yes	Yes	Yes	Yes	Yes	No	Yes	No	6

© 2023 Wang S et al. *JAMA Network Open.*

Study	Events, No.	Total, No.	Treatment rate (95% CI)*	Weight, % (Random)
Year: ~2000				
Canino et al, 2004	108	304	0.36 (0.28−0.44)	6.7
Cuffe et al, 2001	78	212	0.37 (0.28−0.46)	6.6
Leaf et al, 1996	192	415	0.46 (0.39−0.53)	6.7
Sawyer et al, 2018	448	846	0.53 (0.48−0.58)	6.8
Buckner et al, 1997	29	48	0.60 (0.40−0.79)	5.8
Total			0.46 (0.34−0.58)	32.5
Heterogeneity: χ^2_4 = 42.61 (*P* < .001), I^2 = 91%				
Year: 2001~2010				
Borges et al, 2008	165	1204	0.14 (0.11−0.17)	6.8
Zwaanswijk et al, 2005	38	180	0.21 (0.13−0.31)	6.6
Merikangas et al, 2011	1049	2899	0.36 (0.34−0.39)	6.8
Vicente et al, 2012	133	329	0.40 (0.33−0.48)	6.7
Merikangas et al, 2010	366	723	0.51 (0.45−0.56)	6.8
Total			0.32 (0. 14−0.51)	33.7
Heterogeneity: χ^2_4 = 462.73 (*P* < .001), I^2 = 99%				
Year: 2011~2020				
Maalouf et al, 2016	8	133	0.06 (0.02−0.14)	6.7
Fatoriet al, 2019	124	651	0.19 (0.15−0.24)	6.8
Daeem et al, 2019	108	272	0.40 (0.32−0.48)	6.6
Wagner et al, 2017	591	1245	0.47 (0.44−0.51)	6.8
Georgiades et al, 2019	694	1136	0.61 (0.57−0.65)	6.8
Total			0.35 (0.07−0.62)	33.8
Heterogeneity: χ^2_4 = 692.17 (*P* < .001), I^2 = 99%				
Total			0.38 (0.26−0.49)	100.0

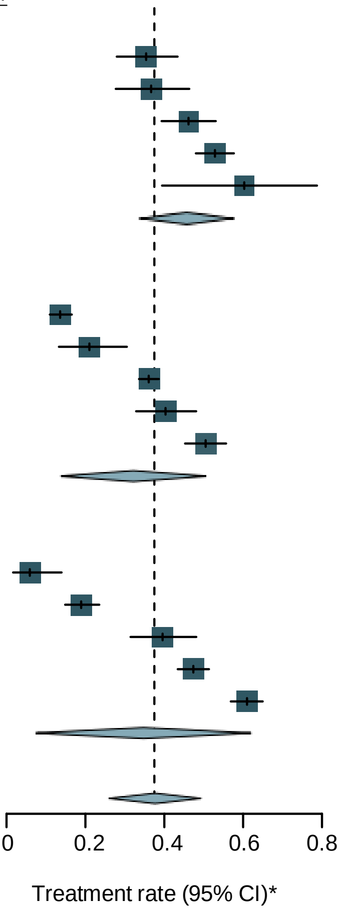

Heterogeneity: χ^2_{14} = 1412.70 (*P* < .001), I^2 = 99%
Test for subgroup differences: χ^2_2 = 3.17 (*P* = .21)

*95% CI adjusted by Bonferroni correction

eFigure 1. Treatment rates for any mental disorder by year of data collection

© 2023 Wang S et al. *JAMA Network Open.*

Study	Events, No.	Total, No.	Treatment rate (95% CI)*	Weight, % (Random)
Year: ~2000				
Essau et al, 2005	42	185	0.23 (0.15−0.32)	5.7
Flament et al, 2001	22	70	0.31 (0.17−0.49)	5.2
Buckner et al, 1997	4	10	0.40 (0.07−0.83)	2.8
Wu et al, 1999	37	80	0.46 (0.31−0.62)	5.2
Stiffman et al, 1988	82	170	0.48 (0.38−0.59)	5.6
Lewinsohn et al, 1998	316	534	0.59 (0.53−0.65)	5.9
Total			0.42 (0.25−0.58)	30.3
Heterogeneity: $\chi^2_5 = 102.95$ ($P < .001$), $I^2 = 95\%$				
Year: 2001~2010				
Zhong et al, 2013	5	86	0.06 (0.01−0.16)	5.8
Coker et al, 2009	58	366	0.16 (0.11−0.22)	5.9
Magklara et al, 2015	42	246	0.17 (0.11−0.25)	5.8
Borges et al, 2008	43	223	0.19 (0.13−0.28)	5.8
Tanielian et al, 2009	53	183	0.29 (0.20−0.39)	5.7
Gould et al, 2009	73	251	0.29 (0.21−0.38)	5.8
Cummings et al, 2011	2904	7618	0.38 (0.37−0.40)	6.0
Merikangas et al, 2011	292	741	0.39 (0.34−0.45)	5.9
Merikangas et al, 2010	128	292	0.44 (0.36−0.52)	5.8
Meredith et al, 2009	103	184	0.56 (0.46−0.66)	5.6
Total			0.29 (0.16−0.43)	58.0
Heterogeneity: $\chi^2_9 = 411.55$ ($P < .001$), $I^2 = 98\%$				
Year: 2011~2020				
Zhang et al, 2021	4221	11553	0.37 (0.35−0.38)	6.0
Wagner et al, 2017	90	215	0.42 (0.33−0.52)	5.7
Total			0.38 (0.31−0.45)	11.7
Heterogeneity: $\chi^2_1 = 2.46$ ($P = .12$), $I^2 = 59\%$				
Total			0.34 (0.24−0.44)	100.0

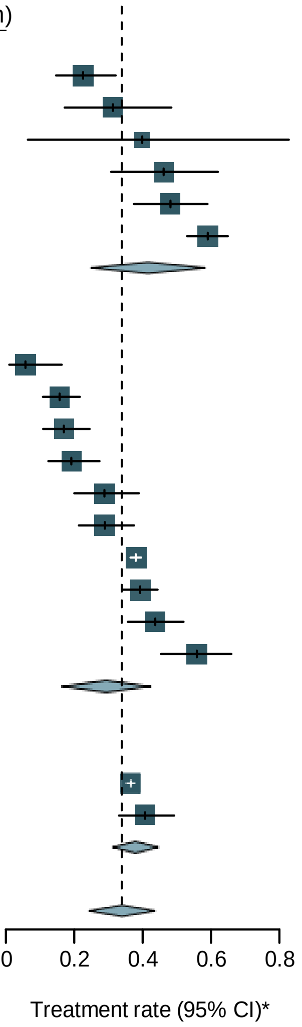

Heterogeneity: $\chi^2_{17} = 578.37$ ($P < .001$), $I^2 = 97\%$
Test for subgroup differences: $\chi^2_2 = 3.41$ ($P = .18$)

*95% CI adjusted by Bonferroni correction

eFigure 2. Treatment rates for depressive disorders by year of data collection

© 2023 Wang S et al. *JAMA Network Open.*

Study	Events, No.	Total, No.	Treatment rate (95% CI)*	Weight, % (Random)
Year: ~2000				
Essau et al, 2005	35	192	0.18 (0.11−0.27)	12.9
Buckner et al, 1997	20	41	0.49 (0.27−0.71)	10.9
Total			0.33 (0.00−0.75)	23.8
Heterogeneity: χ^2_1 = 13.59 (P < .001), I^2 = 93%				
Year: 2001~2010				
Borges et al, 2008	122	917	0.13 (0.10−0.17)	13.2
Merikangas et al, 2011	268	1506	0.18 (0.15−0.21)	13.2
Total			0.16 (0.09−0.22)	26.4
Heterogeneity: χ^2_1 = 9.05 (P = .003), I^2 = 89%				
Year: 2011~2020				
Wagner et al, 2017	148	542	0.27 (0.22−0.33)	13.1
Merikangas et al, 2010	21	65	0.32 (0.18−0.50)	11.8
Niermann et al, 2021	41	125	0.33 (0.22−0.45)	12.5
Reardon et al, 2020	89	138	0.64 (0.52−0.75)	12.5
Total			0.39 (0.15−0.63)	49.9
Heterogeneity: χ^2_3 = 68.41 (P < .001), I^2 = 96%				
Total			0.31 (0.14−0.48)	100.0

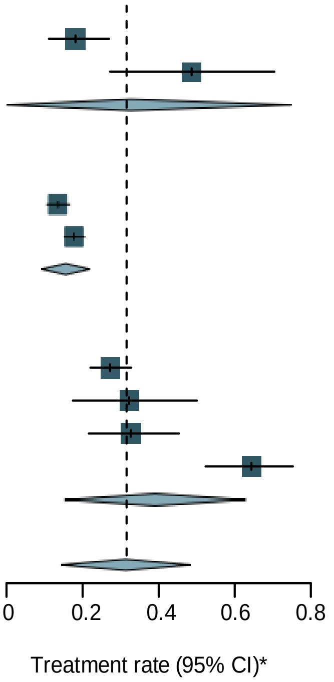

Heterogeneity: χ^2_7 = 201.26 (P < .001), I^2 = 97%
Test for subgroup differences: χ^2_2 = 8.10 (P = .02)

*95% CI adjusted by Bonferroni correction

eFigure 3. Treatment rates for anxiety disorders by year of data collection

Study	Events, No.	Total, No.	Treatment rate (95% CI)*	Weight, % (Random)
Year: ~2000				
Bussing et al, 2003	89	389	0.23 (0.17−0.29)	12.5
Sawyer et al, 2004	112	398	0.28 (0.22−0.35)	12.5
Total			0.25 (0.18−0.33)	25.1
Heterogeneity: χ^2_1 = 2.88 (P = .09), I^2 = 65%				
Year: 2001~2010				
Coker et al, 2009	149	513	0.29 (0.24−0.35)	12.5
Merikangas et al, 2010	218	457	0.48 (0.41−0.54)	12.5
Merikangas et al, 2011	369	617	0.60 (0.54−0.65)	12.6
Heiervang et al, 2007	61	82	0.74 (0.59−0.86)	12.2
Bussing et al, 2011	133	168	0.79 (0.69−0.87)	12.5
Locke et al, 2017	9192	9412	0.98 (0.97−0.98)	12.6
Total			0.65 (0.37−0.92)	74.9
Heterogeneity: χ^2_5 = 2019.42 (P < .001), I^2 = 100%				
Total			0.55 (0.28−0.82)	100.0

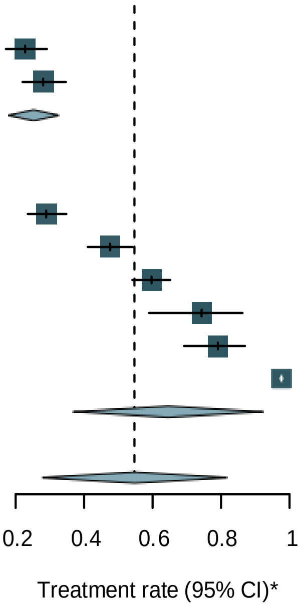

Heterogeneity: χ^2_7 = 4127.42 (P < .001), I^2 = 100%
Test for subgroup differences: χ^2_1 = 14.27 (P < .001)

*95% CI adjusted by Bonferroni correction

eFigure 4. Treatment rates for ADHD by year of data collection

© 2023 Wang S et al. *JAMA Network Open.*

Study	Events, No.	Total, No.	Treatment rate (95% CI)*	Weight, % (Random)
Year: ~2000				
Buckner et al,1997	6	16	0.38 (0.10−0.73)	10.9
Wu et al, 1999	108	132	0.82 (0.71−0.90)	14.7
Total			0.61 (0.00−1.00)	25.7
Heterogeneity: χ^2_1 = 12.45 (P < .001), I^2 = 92%				
Year: 2001~2010				
Coker et al, 2009	231	838	0.28 (0.23−0.32)	15.1
Heievang et al, 2007	65	157	0.41 (0.31−0.53)	14.6
Merikangas et al, 2011	442	974	0.45 (0.41−0.50)	15.1
Total			0.38 (0.22−0.53)	44.8
Heterogeneity: χ^2_2 = 66.04 (P < .001), I^2 = 97%				
Year: 2011~2020				
Wagner et al, 2017	51	130	0.39 (0.28−0.52)	14.5
Georgiades et al, 2019	499	714	0.70 (0.65−0.75)	15.1
Total			0.55 (0.12−0.97)	29.6
Heterogeneity: χ^2_1 = 44.15 (P < .001), I^2 = 98%				
Total			0.49 (0.28−0.70)	100.0

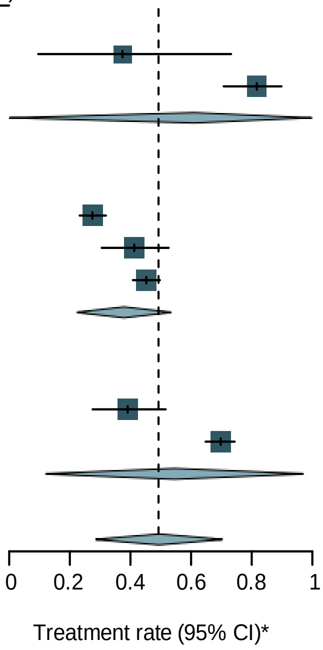

Heterogeneity: χ^2_6 = 449.72 (P < .001), I^2 = 99%
Test for subgroup differences: χ^2_2 = 1.94 (P = .38)

*95% CI adjusted by Bonferroni correction

eFigure 5. Treatment rates for behavior disorders by year of data collection

© 2023 Wang S et al. *JAMA Network Open.*

Study	Events, No.	Total, No.	Treatment rate (95% CI)*	Weight, % (Random)
Region: Americas				
Borges et al, 200	165	1204	0.14 (0.11−0.17)	6.1
Fatoriet al, 2019	124	651	0.19 (0.15−0.24)	6.0
Canino et al, 2004	108	304	0.36 (0.28−0.44)	5.9
Angoldet al, 2002	70	194	0.36 (0.27−0.46)	5.8
Merikangas et al, 2011	1049	2899	0.36 (0.34−0.39)	6.1
Cuffeet al, 2001	78	212	0.37 (0.28−0.46)	5.8
Leaf et al, 1996	192	415	0.46 (0.39−0.53)	5.9
Merikangas et al, 2010	366	723	0.51 (0.45−0.56)	6.0
Sawyer et al, 2018	448	846	0.53 (0.48−0.58)	6.0
Buckner et al, 1997	29	48	0.60 (0.40−0.79)	5.0
Georgiades et al, 2019	694	1136	0.61 (0.57−0.65)	6.0
Total			0.40 (0.28−0.53)	64.7
Heterogeneity: χ^2_{10} = 1110.61 (P < .001), I^2 = 99%				
Region: Europe				
Zwaanswijk et al, 2005	38	180	0.21 (0.13−0.31)	5.9
Gómez−Beneyto et al, 1994	49	114	0.43 (0.30−0.56)	5.6
Wagner et al, 2017	591	1245	0.47 (0.44−0.51)	6.0
Total			0.37 (0.14−0.60)	17.5
Heterogeneity: χ^2_{2} = 61.73 (P < .001), I^2 = 97%				
Region: Eastern Mediterranean				
Maalouf et al, 2016	8	133	0.06 (0.02−0.14)	6.0
Daeem et al, 2019	108	272	0.40 (0.32−0.48)	5.9
Vicente et al, 2012	133	329	0.40 (0.33−0.48)	5.9
Total			0.29 (0.00−0.60)	17.8
Heterogeneity: χ^2_{2} = 140.9 (P < .001), I^2 = 99%				
Total			0.38 (0.27−0.48)	100.0

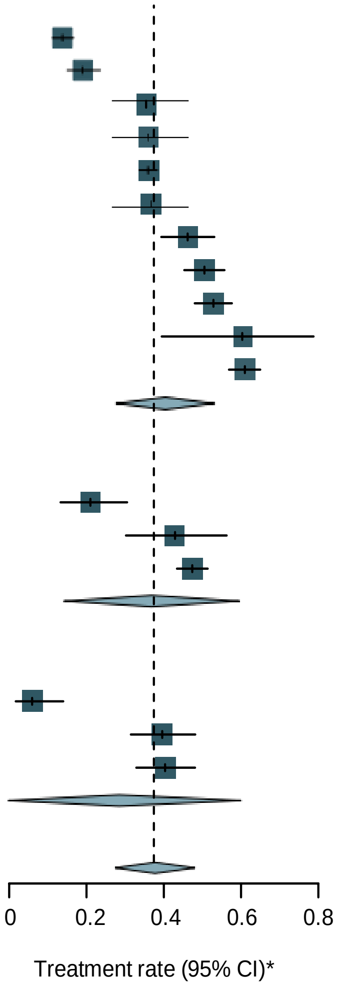

Heterogeneity: χ^2_{16} = 1416.09 (P < .001), I^2 = 99%
Test for subgroup differences: χ^2_{2} = 0.96 (P = .62)

*95% CI adjusted by Bonferroni correction

eFigure 6. Treatment rates for any mental disorder by regions

© 2023 Wang S et al. *JAMA Network Open.*

Study	Events, No.	Total, No.	Treatment rate (95% CI)*	Weight, % (Random)
Region: Americas				
Coker et al, 2009	58	366	0.16 (0.11−0.22)	5.4
Borges et al, 2008	43	223	0.19 (0.13−0.28)	5.3
Tanielian et al, 2009	53	183	0.29 (0.20−0.39)	5.2
Gould et al, 2009	73	251	0.29 (0.21−0.38)	5.3
Zhang et al, 2021	4221	11553	0.37 (0.35−0.38)	5.5
Chavira et al, 2004	3	8	0.38 (0.04−0.85)	2.4
Cummings et al, 2011	2904	7618	0.38 (0.37−0.40)	5.5
Merikangas et al, 2011	292	741	0.39 (0.34−0.45)	5.4
Buckner et al, 1997	4	10	0.40 (0.07−0.83)	2.7
Merikangas et al, 2010	128	292	0.44 (0.36−0.52)	5.3
Wu et al, 1999	37	80	0.46 (0.31−0.62)	4.8
Stiffman et al, 1988	82	170	0.48 (0.38−0.59)	5.2
Meredith et al, 2009	103	184	0.56 (0.46−0.66)	5.2
Lewinsohn et al, 1998	316	534	0.59 (0.53−0.65)	5.4
Wu et al, 2001	131	206	0.64 (0.54−0.73)	5.2
Total			0.40 (0.30−0.51)	73.9
Heterogeneity: χ^2_{14} = 402.83 (P < .001), I^2 = 97%				
Region: Europe				
Magklara et al, 2015	42	246	0.17 (0.11−0.25)	5.4
Essau et al, 2005	42	185	0.23 (0.15−0.32)	5.3
Flament et al, 2001	22	70	0.31 (0.17−0.49)	4.8
Wagner et al, 2017	90	215	0.42 (0.33−0.52)	5.2
Total			0.28 (0.13−0.43)	20.7
Heterogeneity: χ^2_{3} = 37.91 (P < .001), I^2 = 92%				
Region: Western Pacific				
Zhong et al, 2013	5	86	0.06 (0.01−0.16)	5.3
Total			0.36 (0.26−0.46)	100.0

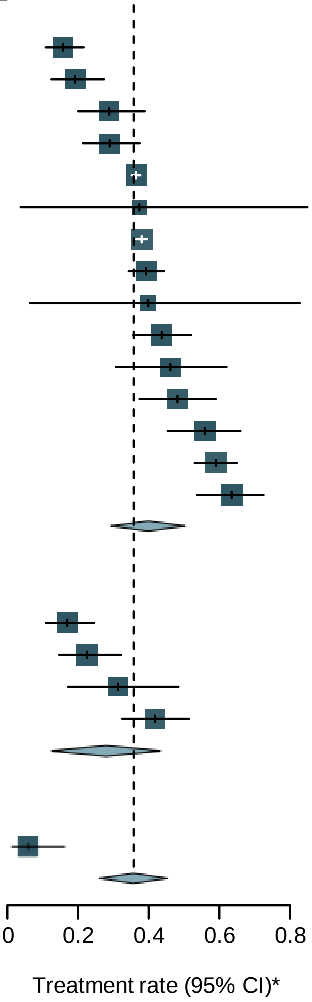

Heterogeneity: χ^2_{19} = 645.13 (P < .001), I^2 = 97%
Test for subgroup differences: χ^2_{2} = 60.27 (P < .001)

*95% CI adjusted by Bonferroni correction

eFigure 7. Treatment rates for depressive disorders by regions

© 2023 Wang S et al. *JAMA Network Open.*

Study	Events, No.	Total, No.	Treatment rate (95% CI)*	Weight, % (Random)
Region: Americas				
Borges et al, 2008	122	917	0.13 (0.10−0.17)	11.9
Merikangas et al, 2011	268	1506	0.18 (0.15−0.21)	11.9
Chavira et al, 2004	21	67	0.31 (0.17−0.49)	10.6
Merikangas et al, 2010	21	65	0.32 (0.18−0.50)	10.5
Buckner et al, 1997	20	41	0.49 (0.27−0.71)	9.6
Total			0.27 (0.11−0.44)	54.4
Heterogeneity: χ^2_4 = 41.2 (P < .001), I^2 = 90%				
Region: Europe				
Essau et al, 2005	35	192	0.18 (0.11−0.27)	11.6
Wagner et al, 2017	148	542	0.27 (0.22−0.33)	11.8
Niermann et al, 2021	41	125	0.33 (0.22−0.45)	11.1
Reardon et al, 2020	89	138	0.64 (0.52−0.75)	11.2
Total			0.36 (0.08−0.63)	45.6
Heterogeneity: χ^2_3 = 91.97 (P < .001), I^2 = 97%				
Total			0.31 (0.16−0.46)	100.0

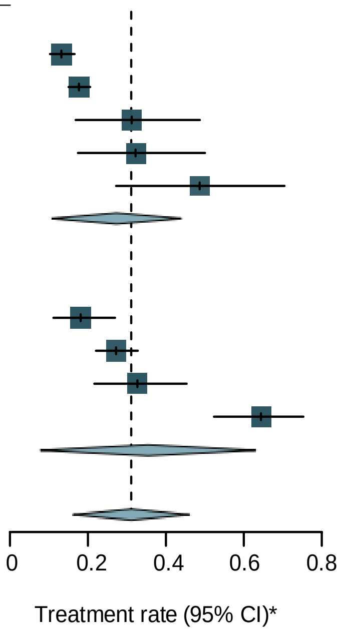

Heterogeneity: χ^2_8 = 205.69 (P < .001), I^2 = 96%
Test for subgroup differences: χ^2_1 = 0.51 (P = .48)

*95% CI adjusted by Bonferroni correction

eFigure 8. Treatment rates for anxiety disorders by regions

Study	Events, No.	Total, No.	Treatment rate (95% CI)*	Weight, % (Random)
Region: Americas				
Bussing et al, 2003	89	389	0.23 (0.17−0.29)	10.1
Sawyer et al, 2004	112	398	0.28 (0.22−0.35)	10.1
Coker et al, 2009	149	513	0.29 (0.24−0.35)	10.1
Merikangas et al, 2010	218	457	0.48 (0.41−0.54)	10.1
Bird et al, 2008	65	109	0.60 (0.46−0.72)	9.9
Merikangas et al, 2011	369	617	0.60 (0.54−0.65)	10.1
Bussing et al, 2011	133	168	0.79 (0.69−0.87)	10.1
Chavira et al, 2004	23	29	0.79 (0.53−0.95)	9.4
Locke et al, 2017	9192	9412	0.98 (0.97−0.98)	10.2
Total			0.56 (0.31−0.80)	90.2
Heterogeneity: χ^2_8 = 4172.18 (P < .001), I^2 = 100%				
Region: Europe				
Heiervang et al, 2007	61	82	0.74 (0.59−0.86)	9.8
Total			0.58 (0.35−0.80)	100.0

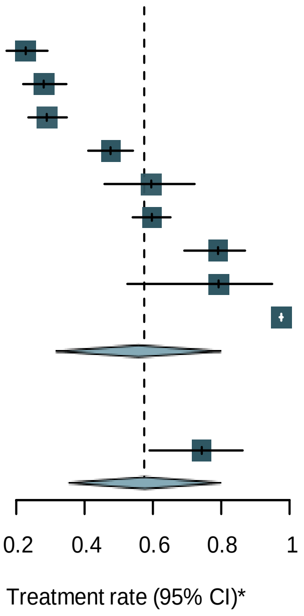

Heterogeneity: χ^2_9 = 4192.28 (P < .001), I^2 = 100%
Test for subgroup differences: χ^2_1 = 3.46 (P = .06)

*95% CI adjusted by Bonferroni correction

eFigure 9. Treatment rates for ADHD by regions

© 2023 Wang S et al. *JAMA Network Open.*

Study	Events, No.	Total, No.	Treatment rate (95% CI)*	Weight, % (Random)
Region: Americas				
Coker et al, 2009	231	838	0.28 (0.23−0.32)	15.1
Buckner et al,1997	6	16	0.38 (0.10−0.73)	10.9
Merikangas et al, 2011	442	974	0.45 (0.41−0.50)	15.1
Georgiades et al, 2019	499	714	0.70 (0.65−0.75)	15.1
Wu et al, 1999	108	132	0.82 (0.71−0.90)	14.7
Total			0.53 (0.24−0.82)	70.9
Heterogeneity: χ^2_4 = 442.27 (P < .001), I^2 = 99%				
Region: Europe				
Wagner et al, 2017	51	130	0.39 (0.28−0.52)	14.5
Heievang et al, 2007	65	157	0.41 (0.31−0.53)	14.6
Total			0.40 (0.32−0.48)	29.1
Heterogeneity: χ^2_1 = 0.14 (P = .71), I^2 = 0%				
Total			0.49 (0.28−0.70)	100.0

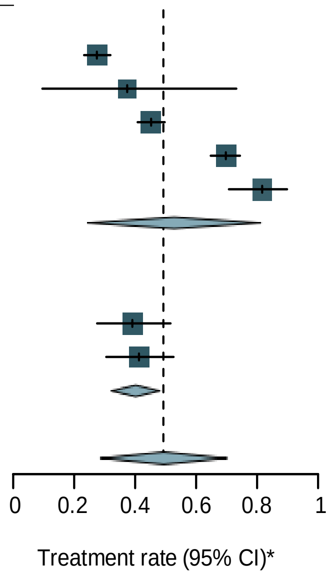

Heterogeneity: χ^2_6 = 449.72 (P < .001), I^2 = 99%
Test for subgroup differences: χ^2_1 = 1.38 (P = .24)

*95% CI adjusted by Bonferroni correction

eFigure 10. Treatment rates for behavior disorders by regions

Study	Events, No.	Total, No.	Treatment rate (95% CI)*	Weight, % (Random)
Age: Children (≤12 years)				
Fatori et al, 2019	124	651	0.19 (0.15−0.24)	10.1
Zwaanswijk et al, 2005	38	180	0.21 (0.13−0.31)	9.9
Cuffe et al, 2001	78	212	0.37 (0.28−0.46)	9.9
Sawyer et al, 2018	200	437	0.46 (0.39−0.53)	10.0
Georgiades et al, 2019	408	664	0.61 (0.56−0.67)	10.0
Total			0.37 (0.15−0.59)	49.8
Heterogeneity: χ^2_4 = 343.6 (P < .001), I^2 = 99%				
Age: Adolescents (13-18 years)				
Maalouf et al, 2016	8	133	0.06 (0.02−0.14)	10.0
Borges et al, 2008	165	1204	0.14 (0.11−0.17)	10.1
Merikangas et al, 2011	1049	2899	0.36 (0.34−0.39)	10.1
Georgiades et al, 2019	286	472	0.61 (0.54−0.67)	10.0
Sawyer et al, 2018	248	409	0.61 (0.54−0.67)	10.0
Total			0.35 (0.04−0.67)	50.2
Heterogeneity: χ^2_4 = 796.05 (P < .001), I^2= 99%				
Total			0.36 (0.18−0.54)	100.0

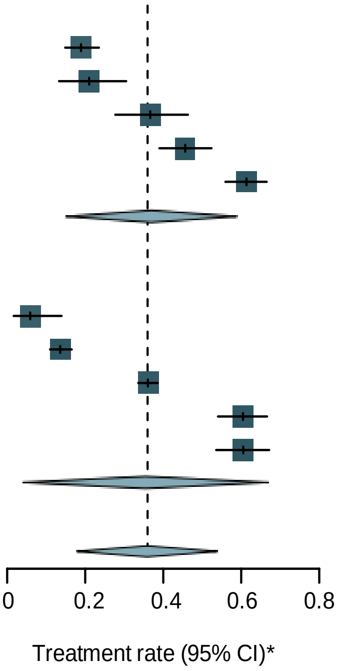

Heterogeneity: χ^2_9 = 1180.85 (P < .001), I^2 = 99%
Test for subgroup differences: χ^2_1 = 0.01 (P = .92)

*95% CI adjusted by Bonferroni correction

eFigure 11. Treatment rates for any mental disorder by age groups

© 2023 Wang S et al. *JAMA Network Open.*

Study	Events, No.	Total, No.	Treatment rate (95% CI)*	Weight, % (Random)
Age: Children (≤12 years)				
Zhong et al, 2013	5	86	0.06 (0.01−0.16)	7.2
Coker et al, 2009	58	366	0.16 (0.11−0.22)	7.3
Total			0.11 (0.00−0.25)	14.5
Heterogeneity: $\chi^2_1 = 10.06$ (P = .002), I^2 = 90%				
Age: Adolescents (13-18 years)				
Magklara et al, 2015	42	246	0.17 (0.11−0.25)	7.2
Borges et al, 2008	43	223	0.19 (0.13−0.28)	7.2
Essau et al, 2005	42	185	0.23 (0.15−0.32)	7.1
Tanielian et al, 2009	53	183	0.29 (0.20−0.39)	7.1
Gould et al, 2009	73	251	0.29 (0.21−0.38)	7.1
Flament et al, 2001	22	70	0.31 (0.17−0.49)	6.5
Zhang et al, 2021	4221	11553	0.37 (0.35−0.38)	7.4
Cummings et al, 2011	2904	7618	0.38 (0.37−0.40)	7.4
Merikangas et al, 2011	292	741	0.39 (0.34−0.45)	7.3
Stiffman et al,1988	82	170	0.48 (0.38−0.59)	7.0
Meredith et al, 2009	103	184	0.56 (0.46−0.66)	7.0
Lewinsohn et al, 1998	316	534	0.59 (0.53−0.65)	7.3
Total			0.36 (0.25−0.46)	85.5
Heterogeneity: $\chi^2_{11} = 301.01$ (P < .001), I^2 = 96%				
Total			0.32 (0.20−0.43)	100.0

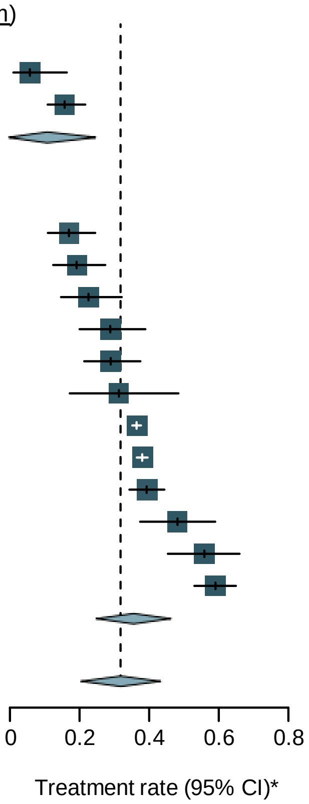

Heterogeneity: $\chi^2_{13} = 564.58$ (P < .001), I^2 = 98%
Test for subgroup differences: $\chi^2_1 = 14.82$ (P < .001)

*95% CI adjusted by Bonferroni correction

eFigure 12. Treatment rates for depressive disorders by age groups

Study	Events, No.	Total, No.	Treatment rate (95% CI)*	Weight, % (Random)
Age: Children (≤12 years)				
Reardon et al, 2020	89	138	0.64 (0.52−0.75)	19.6
Age: Adolescents (13-18 years)				
Borges et al, 2008	122	917	0.13 (0.10−0.17)	20.4
Merikangas et al, 2011	268	1506	0.18 (0.15−0.21)	20.4
Essau et al, 2005	35	192	0.18 (0.11−0.27)	20.0
Niermann et al, 2021	41	125	0.33 (0.22−0.45)	19.6
Total			0.20 (0.09−0.30)	80.4
Heterogeneity: $\chi^2_3 = 25.3$ (P < .001), I^2 = 88%				
Total			0.29 (0.03−0.55)	100.0

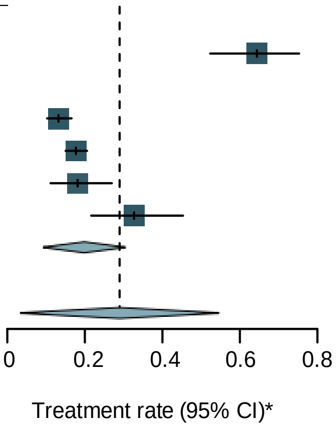

Heterogeneity: $\chi^2_4 = 160.23$ (P < .001), I^2 = 98%
Test for subgroup differences: $\chi^2_1 = 63.73$ (P < .001)

*95% CI adjusted by Bonferroni correction

eFigure 13. Treatment rates for anxiety disorders by age groups

© 2023 Wang S et al. *JAMA Network Open.*

Study	Events, No.	Total, No.	Treatment rate (95% CI)*	Weight, % (Random)
Age: Children (≤12 years)				
Bussing et al, 2003	89	389	0.23 (0.17−0.29)	16.9
Coker et al, 2009	149	513	0.29 (0.24−0.35)	16.9
Bird et al, 2008	65	109	0.60 (0.46−0.72)	16.3
Heiervang et al, 2007	61	82	0.74 (0.59−0.86)	16.3
Bussing et al, 2011	133	168	0.79 (0.69−0.87)	16.7
Total			0.53 (0.21−0.85)	83.1
Heterogeneity: $\chi^2_4 = 319.08$ (P < .001), I^2 = 99%				
Age: Adolescents (13-18 years)				
Merikangas et al, 2011	369	617	0.60 (0.54−0.65)	16.9
Total			0.54 (0.28−0.80)	100.0

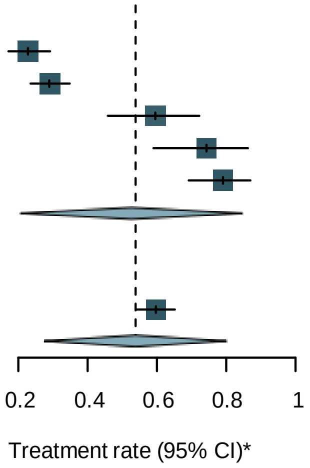

Heterogeneity: $\chi^2_5 = 393.15$ (P < .001), I^2 = 99%
Test for subgroup differences: $\chi^2_1 = 0.35$ (P = .55)

*95% CI adjusted by Bonferroni correction

eFigure 14. Treatment rates for ADHD by age groups

Study	Events, No.	Total, No.	Treatment rate (95% CI)*	Weight, % (Random)
Age: Children (≤12 years)				
Coker et al, 2009	231	838	0.28 (0.23−0.32)	20.2
Heievang et al, 2007	65	157	0.41 (0.31−0.53)	19.5
Georgiades et al, 2019	316	448	0.71 (0.64−0.76)	20.1
Total			0.47 (0.11−0.82)	59.9
Heterogeneity: $\chi^2_2 = 262.99$ (P < .001), I^2 = 99%				
Age: Adolescents (13-18 years)				
Merikangas et al, 2011	442	974	0.45 (0.41−0.50)	20.2
Georgiades et al, 2019	183	266	0.69 (0.60−0.76)	19.9
Total			0.57 (0.25−0.89)	40.1
Heterogeneity: $\chi^2_1 = 51.66$ (P < .001), I^2 = 98%				
Total			0.51 (0.28−0.74)	100.0

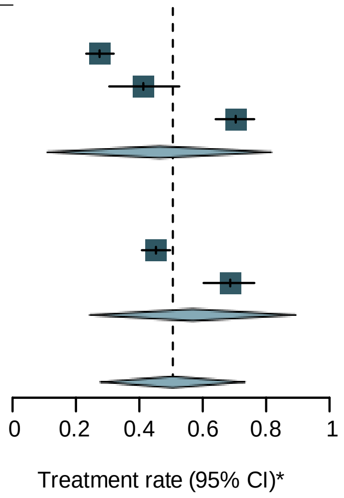

Heterogeneity: $\chi^2_4 = 338.29$ (P < .001), I^2 = 99%
Test for subgroup differences: $\chi^2_1 = 0.37$ (P = .55)

*95% CI adjusted by Bonferroni correction

eFigure 15. Treatment rates for behavior disorders by age groups

© 2023 Wang S et al. *JAMA Network Open.*

Study	Events, No.	Total, No.	Treatment rate (95% CI)*	Weight, % (Random)
Income level: High				
Zwaanswijk et al, 2005	38	180	0.21 (0.13−0.31)	5.9
Canino et al, 2004	108	304	0.36 (0.28−0.44)	5.9
Angold et al, 2002	70	194	0.36 (0.27−0.46)	5.8
Merikangas et al, 2011	1049	2899	0.36 (0.34−0.39)	6.1
Cuffe et al, 2001	78	212	0.37 (0.28−0.46)	5.8
Daeem et al, 2019	108	272	0.40 (0.32−0.48)	5.9
Gómez−Beneyto et al, 1994	49	114	0.43 (0.30−0.56)	5.6
Leaf et al, 1996	192	415	0.46 (0.39−0.53)	5.9
Wagner et al, 2017	591	1245	0.47 (0.44−0.51)	6.0
Merikangas et al, 2010	366	723	0.51 (0.45−0.56)	6.0
Sawyer et al, 2018	448	846	0.53 (0.48−0.58)	6.0
Buckner et al, 1997	29	48	0.60 (0.40−0.79)	5.0
Georgiades et al, 2019	694	1136	0.61 (0.57−0.65)	6.0
Total			0.43 (0.35−0.52)	76.0
Heterogeneity: $\chi^2_{12} = 346.76$ ($P < .001$), $I^2 = 97\%$				
Income level: Upper middle				
Borges et al, 2008	165	1204	0.14 (0.11−0.17)	6.1
Fatori et al, 2019	124	651	0.19 (0.15−0.24)	6.0
Vicente et al, 2012	133	329	0.40 (0.33−0.48)	5.9
Total			0.24 (0.02−0.47)	18.0
Heterogeneity: $\chi^2_{2} = 87.47$ ($P < .001$), $I^2 = 98\%$				
Income level: Lower middle				
Maalouf et al, 2016	8	133	0.06 (0.02−0.14)	6.0
Total			0.38 (0.27−0.48)	100.0

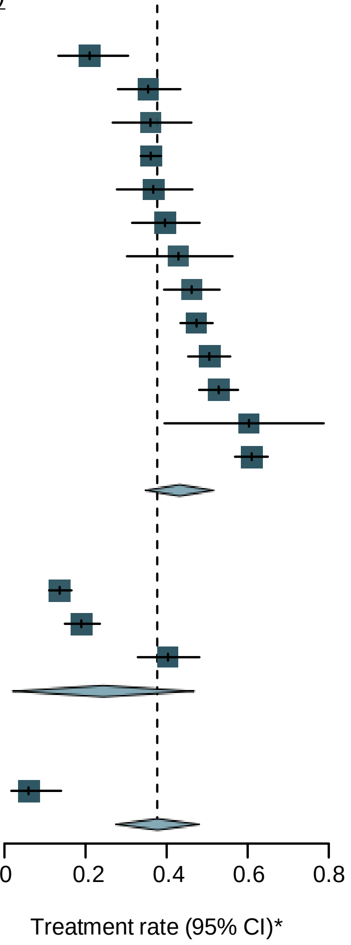

Heterogeneity: $\chi^2_{16} = 1416.09$ ($P < .001$), $I^2 = 99\%$

Test for subgroup differences: $\chi^2_{2} = 104.70$ ($P < .001$)

*95% CI adjusted by Bonferroni correction

eFigure 16. Treatment rates for any mental disorder by income groups

© 2023 Wang S et al. *JAMA Network Open.*

Study	Events, No.	Total, No.	Treatment rate (95% CI)*	Weight, % (Random)
Income level: High				
Coker et al, 2009	58	366	0.16 (0.11−0.22)	5.4
Magklara et al, 2015	42	246	0.17 (0.11−0.25)	5.4
Essau et al, 2005	42	185	0.23 (0.15−0.32)	5.3
Tanielian et al, 2009	53	183	0.29 (0.20−0.39)	5.2
Gould et al, 2009	73	251	0.29 (0.21−0.38)	5.3
Flament et al, 2001	22	70	0.31 (0.17−0.49)	4.8
Zhang et al, 2021	4221	11553	0.37 (0.35−0.38)	5.5
Chavira et al, 2004	3	8	0.38 (0.04−0.85)	2.4
Cummings et al, 2011	2904	7618	0.38 (0.37−0.40)	5.5
Merikangas et al, 2011	292	741	0.39 (0.34−0.45)	5.4
Buckner et al, 1997	4	10	0.40 (0.07−0.83)	2.7
Wagner et al, 2017	90	215	0.42 (0.33−0.52)	5.2
Merikangas et al, 2010	128	292	0.44 (0.36−0.52)	5.3
Wu et al, 1999	37	80	0.46 (0.31−0.62)	4.8
Stiffman et al, 1988	82	170	0.48 (0.38−0.59)	5.2
Meredith et al, 2009	103	184	0.56 (0.46−0.66)	5.2
Lewinsohn et al, 1998	316	534	0.59 (0.53−0.65)	5.4
Wu et al, 2001	131	206	0.64 (0.54−0.73)	5.2
Total			0.39 (0.29−0.48)	89.3
Heterogeneity: χ^2_{17} = 452.16 (P < .001), I^2 = 96%				
Income level: Upper middle				
Zhong et al, 2013	5	86	0.06 (0.01−0.16)	5.3
Borges et al, 2008	43	223	0.19 (0.13−0.28)	5.3
Total			0.13 (0.00−0.31)	10.7
Heterogeneity: χ^2_{1} = 13.59 (P < .001), I^2 = 93%				
Total			0.36 (0.26−0.46)	100.0

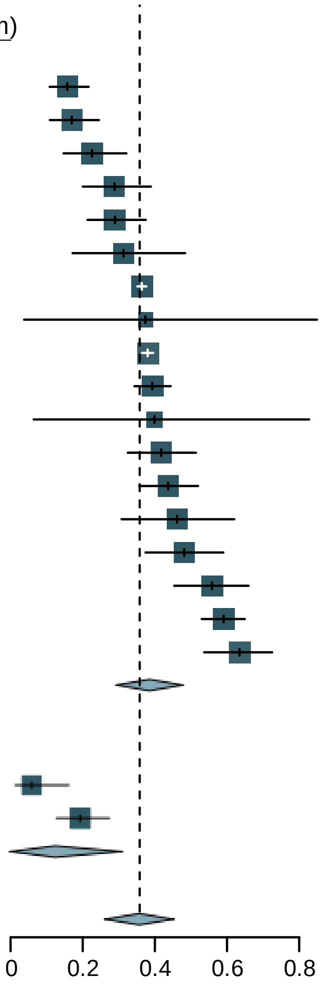

Heterogeneity: χ^2_{19} = 645.13 (P < .001), I^2 = 97%
Test for subgroup differences: χ^2_{1} = 11.93 (P < .001)

*95% CI adjusted by Bonferroni correction

eFigure 17. Treatment rates for depressive disorders by income groups

© 2023 Wang S et al. *JAMA Network Open.*

Study	Events, No.	Total, No.	Treatment rate (95% CI)*	Weight, % (Random)
Income level: High				
Merikangas et al, 2011	268	1506	0.18 (0.15−0.21)	11.9
Essau et al, 2005	35	192	0.18 (0.11−0.27)	11.6
Wagner et al, 2017	148	542	0.27 (0.22−0.33)	11.8
Chavira et al, 2004	21	67	0.31 (0.17−0.49)	10.6
Merikangas et al, 2010	21	65	0.32 (0.18−0.50)	10.5
Niermann et al, 2021	41	125	0.33 (0.22−0.45)	11.1
Buckner et al, 1997	20	41	0.49 (0.27−0.71)	9.6
Reardon et al, 2020	89	138	0.64 (0.52−0.75)	11.2
Total			0.34 (0.18−0.49)	88.1
Heterogeneity: χ^2_7 = 160.35 (P < .001), I^2 = 96%				
Income level: Upper middle				
Borges et al, 2008	122	917	0.13 (0.10−0.17)	11.9
Total			0.31 (0.16−0.46)	100.0

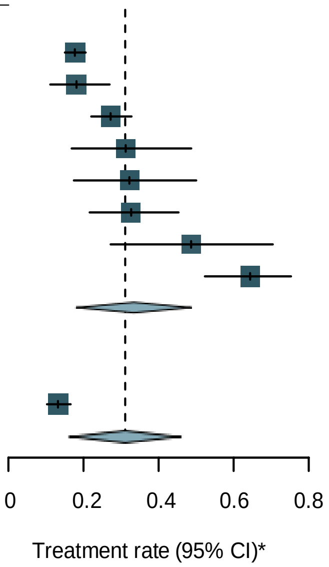

Heterogeneity: χ^2_8 = 205.69 (P < .001), I^2 = 96%

Test for subgroup differences: χ^2_1 = 12.61 (P < .001)

*95% CI adjusted by Bonferroni correction

eFigure 18. Treatment rates for anxiety disorders by income groups

Outcomes	Study, No.	Events, No.	Total, No.	Treatment rate (95% CI)*
Current				
ADHD	1	89	389	0.23 (0.17−0.29)
Last 3 months				
Any mental disorder	1	70	194	0.36 (0.27−0.46)
Last 6 months				
Any mental disorder	2	711	1184	0.49 (0.14−0.85)
Depressive disorders	3	158	377	0.37 (0.07−0.67)
Anxiety disorders	1	11	41	0.27 (0.11−0.49)
ADHD	1	112	398	0.35 (0.15−0.54)
Behavior disorders	2	504	730	0.52 (0.00−1.00)
Last year				
Any mental disorder	7	1451	4001	0.37 (0.22−0.53)
Depressive disorders	10	7655	20647	0.37 (0.24−0.49)
Anxiety disorders	2	143	982	0.22 (0.00−0.48)
ADHD	3	354	734	0.53 (0.37−0.69)
Behavior disorders	1	80	132	0.61 (0.48−0.72)
Lifetime				
Any mental disorder	8	2036	5574	0.36 (0.19−0.52)
Depressive disorders	9	866	2287	0.33 (0.17−0.49)
Anxiety disorders	7	622	2611	0.34 (0.16−0.52)
ADHD	6	9927	10821	0.70 (0.43−0.97)
Behavior disorders	6	903	2247	0.46 (0.24−0.68)

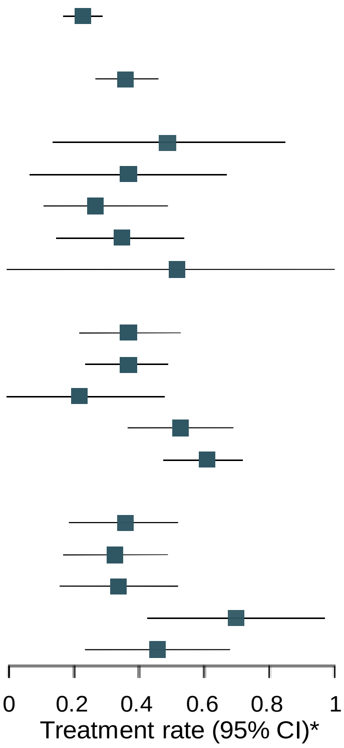

*95% CI adjusted by Bonferroni correction

eFigure 19. Subgroup by timeframe

© 2023 Wang S et al. *JAMA Network Open.*

Study	Events, No	Total, No.	Treatment rate (95% CI)*	Weight, % (Random)
Timeframe: Last 3 months				
Angold et al, 2002	70	194	0.36 (0.27−0.46)	5.5
Timeframe: Last 6 months				
Buckner et al, 1997	17	48	0.35 (0.18−0.56)	4.8
Georgiades et al, 2019	694	1136	0.61 (0.57−0.65)	5.8
Total			0.49 (0.14−0.85)	10.5
Heterogeneity: $\chi^2_1 = 13.25$ ($P < .001$), $I^2 = 92\%$				
Timeframe: Last year				
Borges et al, 2008	165	1204	0.14 (0.11−0.17)	5.8
Zwaanswijket al, 2005	38	180	0.21 (0.13−0.31)	5.6
Canino et al, 2004	108	304	0.36 (0.28−0.44)	5.6
Vicente et al, 2012	133	329	0.40 (0.33−0.48)	5.6
Leaf et al, 1996	192	415	0.46 (0.39−0.53)	5.7
Merikangas et al, 2010	366	723	0.51 (0.45−0.56)	5.7
Sawyer et al, 2018	448	846	0.53 (0.48−0.58)	5.7
Total			0.37 (0.22−0.53)	39.7
Heterogeneity: $\chi^2_6 = 643.37$ ($P < .001$), $I^2 = 99\%$				
Timeframe: Lifetime				
Maalouf et al, 2016	8	133	0.06 (0.02−0.14)	5.7
Fatori et al, 2019	124	651	0.19 (0.15−0.24)	5.8
Merikangas et al, 2011	1049	2899	0.36 (0.34−0.39)	5.8
Cuffe et al, 2001	78	212	0.37 (0.28−0.46)	5.5
Daeem et al, 2019	108	272	0.40 (0.32−0.48)	5.6
Gómez−Beneyto et al, 1994	49	114	0.43 (0.30−0.56)	5.3
Wagner et al, 2017	591	1245	0.47 (0.44−0.51)	5.8
Buckner et al, 1997	29	48	0.60 (0.40−0.79)	4.7
Total			0.36 (0.19−0.52)	44.2
Heterogeneity: $\chi^2_7 = 396.46$ ($P < .001$), $I^2 = 98\%$				
Total			0.38 (0.28−0.48)	100.0

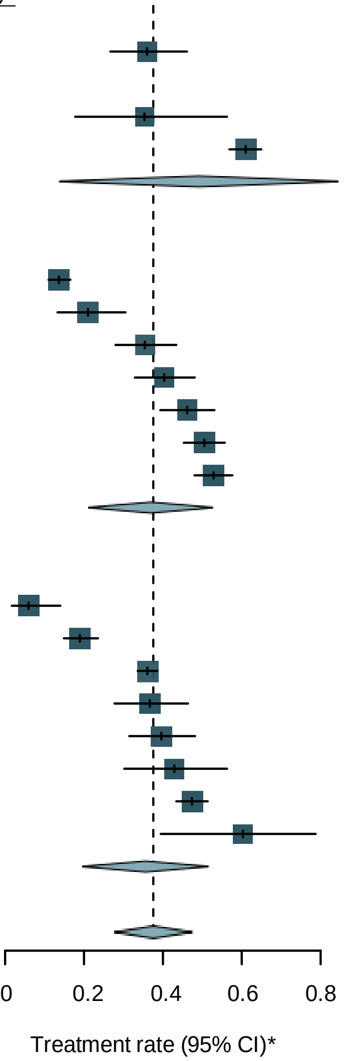

Heterogeneity: $\chi^2_{17} = 1416.10$ ($P < .001$), $I^2 = 99\%$

Test for subgroup differences: $\chi^2_3 = 1.02$ ($P = .80$)

*95% CI adjusted by Bonferroni correction

eFigure 20. Treatment rates for any mental disorder by timeframes

© 2023 Wang S et al. *JAMA Network Open.*

Study	Events, No.	Total, No.	Treatment rate (95% CI)*	Weight, % (Random)
Timeframe: Last 6 months				
Buckner et al, 1997	2	10	0.20 (0.01−0.68)	2.9
Tanielian et al, 2009	53	183	0.29 (0.20−0.39)	4.9
Meredith et al, 2009	103	184	0.56 (0.46−0.66)	4.8
Total			0.37 (0.07−0.67)	12.6
Heterogeneity: $\chi^2_2 = 32.36$ ($P < .001$), $I^2 = 94\%$				
Timeframe: Last year				
Magklara et al, 2015	42	246	0.17 (0.11−0.25)	5.0
Borges et al, 2008	43	223	0.19 (0.13−0.28)	4.9
Gould et al, 2009	73	251	0.29 (0.21−0.38)	4.9
Wu et al, 1999	28	80	0.35 (0.21−0.51)	4.5
Zhang et al, 2021	4221	11553	0.37 (0.35−0.38)	5.1
Chavira et al, 2004	3	8	0.38 (0.04−0.85)	2.2
Cummings et al, 2011	2904	7618	0.38 (0.37−0.40)	5.1
Merikangas et al, 2010	128	292	0.44 (0.36−0.52)	4.9
Stiffman et al, 1988	82	170	0.48 (0.38−0.59)	4.8
Wu et al, 2001	131	206	0.64 (0.54−0.73)	4.9
Total			0.37 (0.24−0.49)	46.3
Heterogeneity: $\chi^2_9 = 203.56$ ($P < .001$), $I^2 = 96\%$				
Timeframe: Lifetime				
Zhong et al, 2013	5	86	0.06 (0.01−0.16)	5.0
Coker et al, 2009	58	366	0.16 (0.11−0.22)	5.0
Essau et al, 2005	42	185	0.23 (0.15−0.32)	4.9
Flament et al, 2001	22	70	0.31 (0.17−0.49)	4.5
Merikangas et al, 2011	292	741	0.39 (0.34−0.45)	5.0
Buckner et al, 1997	4	10	0.40 (0.07−0.83)	2.4
Wagner et al, 2017	90	215	0.42 (0.33−0.52)	4.9
Wu et al, 1999	37	80	0.46 (0.31−0.62)	4.5
Lewinsohn et al, 1998	316	534	0.59 (0.53−0.65)	5.0
Total			0.33 (0.17−0.49)	41.1
Heterogeneity: $\chi^2_8 = 384.07$ ($P < .001$), $I^2 = 98\%$				
Total			0.35 (0.26−0.45)	100.0

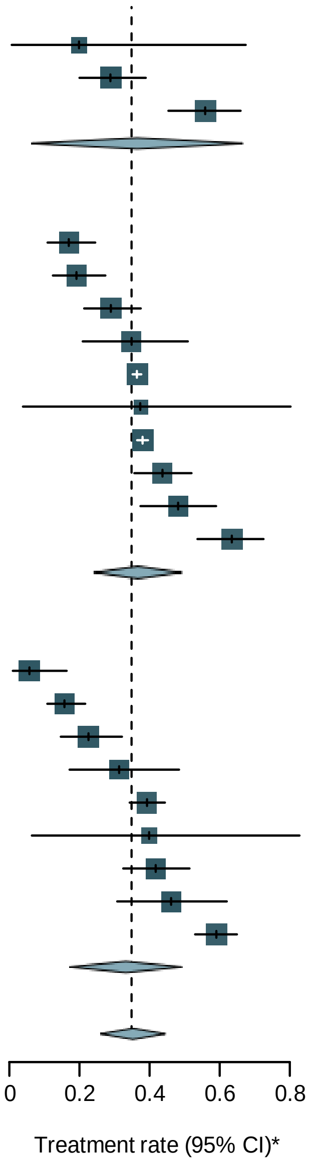

Heterogeneity: $\chi^2_{21} = 646.85$ ($P < .001$), $I^2 = 97\%$
Test for subgroup differences: $\chi^2_2 = 0.24$ ($P = .89$)

*95% CI adjusted by Bonferroni correction

eFigure 21. Treatment rates for depressive disorders by timeframes

© 2023 Wang S et al. *JAMA Network Open.*

Study	Events, No.	Total, No.	Treatment rate (95% CI)*	Weight, % (Random)
Timeframe: Last 6 months				
Buckner et al, 1997	11	41	0.27 (0.11–0.49)	9.0
Timeframe: Last year				
Borges et al, 2008	122	917	0.13 (0.10–0.17)	10.9
Merikangas et al, 2010	21	65	0.32 (0.18–0.50)	9.5
Total			0.22 (0.00–0.48)	20.4
Heterogeneity: χ^2_1 = 10.35 (*P* = .001), I^2 = 90%				
Timeframe: Lifetime				
Merikangas et al, 2011	268	1506	0.18 (0.15–0.21)	10.9
Essau et al, 2005	35	192	0.18 (0.11–0.27)	10.6
Wagner et al, 2017	148	542	0.27 (0.22–0.33)	10.8
Chavira et al, 2004	21	67	0.31 (0.17–0.49)	9.6
Niermann et al, 2021	41	125	0.33 (0.22–0.45)	10.1
Buckner et al, 1997	20	41	0.49 (0.27–0.71)	8.6
Reardon et al, 2020	89	138	0.64 (0.52–0.75)	10.2
Total			0.34 (0.16–0.52)	70.6
Heterogeneity: χ^2_6 = 157.45 (*P* < .001), I^2 = 96%				
Total			0.31 (0.17–0.44)	100.0

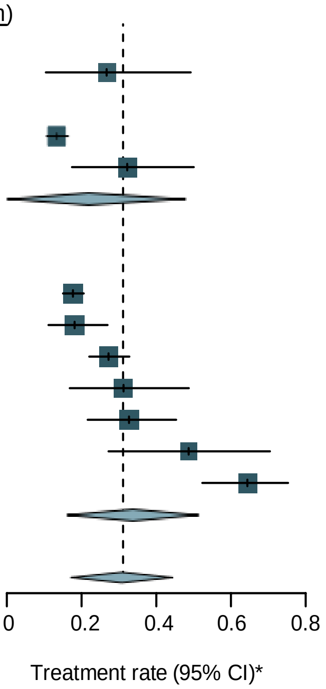

Heterogeneity: χ^2_9 = 206.81 (*P* < .001), I^2 = 96%
Test for subgroup differences: χ^2_2 = 1.23 (*P* = .54)

*95% CI adjusted by Bonferroni correction

eFigure 22. Treatment rates for anxiety disorders by timeframes

Study	Events, No.	Total, No.	Treatment rate (95% CI)*	Weight, % (Random)
Timeframe: Current				
Bussing et al, 2003	89	389	0.23 (0.17–0.29)	9.2
Timeframe: Last 6 months				
Sawyer et al, 2004	112	398	0.28 (0.22–0.35)	9.2
Bussing et al, 2011	71	168	0.42 (0.32–0.53)	9.1
Total			0.35 (0.15–0.54)	18.3
Heterogeneity: χ^2_1 = 10.17 (*P* = .001), I^2 = 90%				
Timeframe: Last year				
Merikangas et al, 2010	218	457	0.48 (0.41–0.54)	9.2
Bird et al, 2008	65	109	0.60 (0.46–0.72)	9.0
Tota			0.53 (0.37–0.69)	18.2
Heterogeneity: χ^2_1 = 5.17 (*P* = .02), I^2 = 81%				
Timeframe: Lifetime				
Coker et al, 2009	149	513	0.29 (0.24–0.35)	9.2
Merikangas et al, 2011	369	617	0.60 (0.54–0.65)	9.2
Heiervang et al, 2007	61	82	0.74 (0.59–0.86)	8.9
Bussing et al, 2011	133	168	0.79 (0.69–0.87)	9.1
Chavira et al, 2004	23	29	0.79 (0.53–0.95)	8.5
Locke et al, 2017	9192	9412	0.98 (0.97–0.98)	9.3
Total			0.70 (0.43–0.97)	54.3
Heterogeneity: χ^2_5 = 1582.49 (*P* < .001), I^2 = 100%				
Total			0.56 (0.36–0.77)	100.0

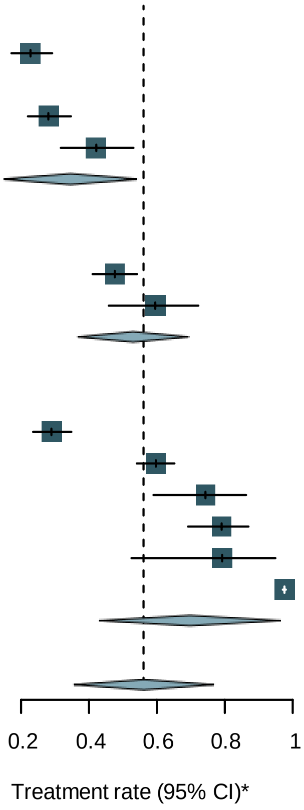

Heterogeneity: χ^2_{10} = 4390.73 (*P* < .001), I^2 = 100%
Test for subgroup differences: χ^2_3 = 42.78 (*P* < .001)

*95% CI adjusted by Bonferroni correction

© 2023 Wang S et al. *JAMA Network Open.*

eFigure 23. Treatment rates for ADHD by timeframes

Study	Events, No.	Total, No.	Treatment rate (95% CI)*	Weight, % (Random)
Timeframe: Last 6 months				
Buckner et al,1997	5	16	0.31 (0.07−0.68)	8.7
Georgiades et al, 2019	499	714	0.70 (0.65−0.75)	12.1
Total			0.52 (0.00−1.00)	20.8
Heterogeneity: $\chi^2_1 = 10.88$ ($P < .001$), $I^2 = 91\%$				
Timeframe: Last year				
Wu et al, 1999	80	132	0.61 (0.48−0.72)	11.6
Timeframe: Lifetime				
Coker et al, 200	231	838	0.28 (0.23−0.32)	12.1
Buckner et al,1997	6	16	0.38 (0.10−0.73)	8.4
Wagner et al, 2017	51	130	0.39 (0.28−0.52)	11.6
Heievang et al, 2007	65	157	0.41 (0.31−0.53)	11.7
Merikangas et al, 2011	442	974	0.45 (0.41−0.50)	12.1
Wu et al, 1999	108	132	0.82 (0.71−0.90)	11.8
Total			0.46 (0.24−0.68)	67.7
Heterogeneity: $\chi^2_5 = 231.24$ ($P < .001$), $I^2 = 98\%$				
Total			0.49 (0.32−0.67)	100.0

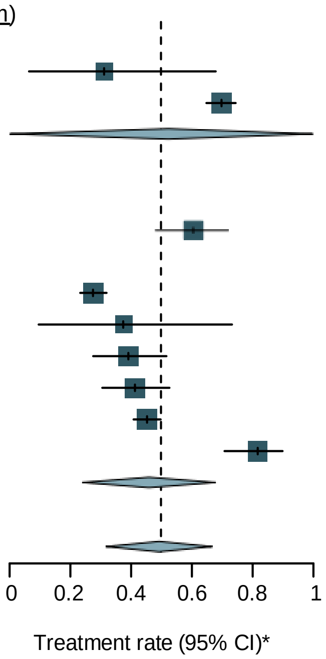

Heterogeneity: $\chi^2_8 = 460.49$ ($P < .001$), $I^2 = 98\%$
Test for subgroup differences: $\chi^2_2 = 2.75$ ($P = .25$)

*95% CI adjusted by Bonferroni correction

eFigure 24. Treatment rates for behavior disorders by timeframes

Outcomes	Study, No.	Events, No.	Patients, No.	Treatment rate (95% CI)*
Child				
Any mental disorder	5	1379	3762	0.40 (0.21−0.58)
Depressive disorders	12	8066	21541	0.37 (0.26−0.48)
Anxiety disorders	5	367	1841	0.24 (0.13−0.35)
ADHD	3	9475	9978	0.68 (0.26−1.00)
Behavior disorders	2	147	324	0.45 (0.30−0.59)
Parent				
Any mental disorder	7	1449	3266	0.37 (0.14−0.60)
Depressive disorders	3	65	384	0.25 (0.00−0.50)
Anxiety disorders	3	130	246	0.48 (0.21−0.76)
ADHD	6	567	1579	0.52 (0.20−0.83)
Behavior disorders	4	801	1725	0.45 (0.18−0.72)
Parent and Child				
Any mental disorder	6	1630	4353	0.39 (0.34−0.43)
Depressive disorders	5	518	1296	0.37 (0.10−0.63)
Anxiety disorders	1	268	1506	0.18 (0.15−0.21)
ADHD	1	369	617	0.60 (0.54−0.65)
Behavior disorders	2	550	1106	0.63 (0.13−1.00)

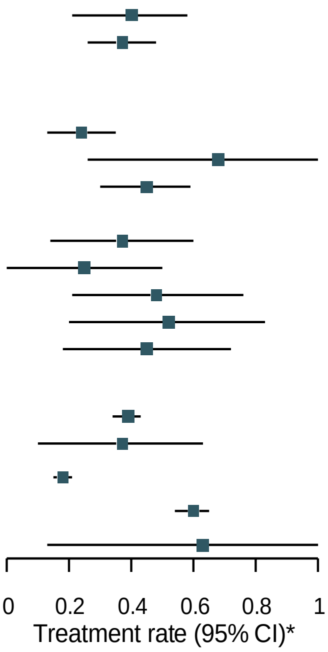

*95% CI adjusted by Bonferroni correction

eFigure 25. Subgroup by informant source

© 2023 Wang S et al. *JAMA Network Open.*

Study	Events, No.	Total, No.	Treatment rate (95% CI)*	Weight, % (Random)
Informant source: Child				
Borges et al, 2008	165	1204	0.14 (0.11−0.17)	5.7
Gómez−Beneyto et al, 1994	49	114	0.43 (0.30−0.56)	5.3
Georgiades et al, 2019	208	476	0.44 (0.37−0.50)	5.6
Wagner et al, 2017	591	1245	0.47 (0.44−0.51)	5.7
Merikangas et al, 2010	366	723	0.51 (0.45−0.56)	5.7
Total			0.40 (0.21−0.58)	28.0
Heterogeneity: χ^2_4 = 588.99 (P < .001), I^2 = 99%				
Informant source: Parent				
Maalouf et al, 2016	8	133	0.06 (0.02−0.14)	5.7
Fatoriet al, 2019	124	651	0.19 (0.15−0.24)	5.7
Zwaanswijket al, 2005	38	180	0.21 (0.13−0.31)	5.5
Daeem et al, 2019	108	272	0.40 (0.32−0.48)	5.5
Sawyer et al, 2018	448	846	0.53 (0.48−0.58)	5.7
Buckner et al, 1997	29	48	0.60 (0.40−0.79)	4.7
Georgiades et al, 2019	694	1136	0.61 (0.57−0.65)	5.7
Total			0.37 (0.14−0.60)	38.5
Heterogeneity: χ^2_6 = 764.16 (P < .001), I^2 = 99%				
Informant source: Parent and child				
Canino et al, 2004	108	304	0.36 (0.28−0.44)	5.6
Angoldet al, 2002	70	194	0.36 (0.27−0.46)	5.5
Merikangas et al, 2011	1049	2899	0.36 (0.34−0.39)	5.7
Cuffe et al, 2001	78	212	0.37 (0.28−0.46)	5.5
Vicente et al, 2012	133	329	0.40 (0.33−0.48)	5.6
Leaf et al, 1996	192	415	0.46 (0.39−0.53)	5.6
Total			0.39 (0.34−0.43)	33.5
Heterogeneity: χ^2_5 = 16.87 (P = .005), I^2 = 70%				
Total			0.38 (0.28−0.48)	100.0

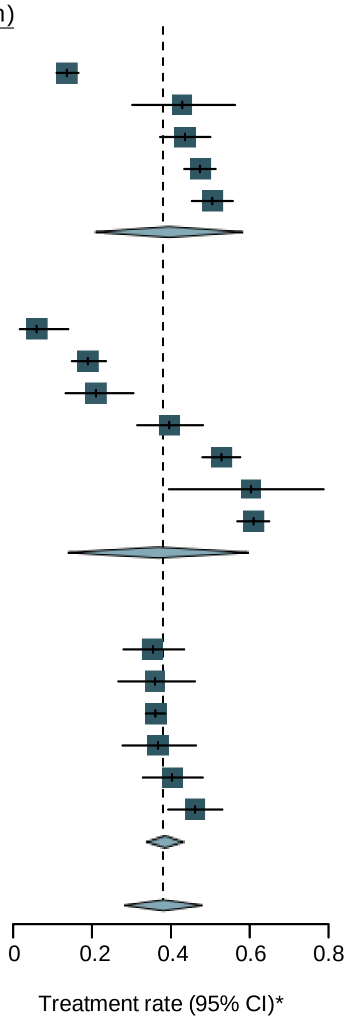

Heterogeneity: χ^2_{17} = 1431.18 (P < .001), I^2 = 99%
Test for subgroup differences: χ^2_2 = 0.06 (P = .97)

*95% CI adjusted by Bonferroni correction

eFigure 26. Treatment rates for any mental disorder by informant sources

© 2023 Wang S et al. *JAMA Network Open.*

Study	Events, No.	Total, No.	Treatment rate (95% CI)*	Weight, % (Random)
Informant source: Child				
Magklara et al, 2015	42	246	0.17 (0.11−0.25)	5.4
Borges et al, 2008	43	223	0.19 (0.13−0.28)	5.3
Essau et al, 2005	42	185	0.23 (0.15−0.32)	5.3
Gould et al, 2009	73	251	0.29 (0.21−0.38)	5.3
Flament et al, 2001	22	70	0.31 (0.17−0.49)	4.8
Zhang et al, 2021	4221	11553	0.37 (0.35−0.38)	5.5
Cummings et al, 2011	2904	7618	0.38 (0.37−0.40)	5.5
Wagner et al, 2017	90	215	0.42 (0.33−0.52)	5.2
Merikangas et al, 2010	128	292	0.44 (0.36−0.52)	5.3
Stiffman et al, 1988	82	170	0.48 (0.38−0.59)	5.2
Meredith et al, 2009	103	184	0.56 (0.46−0.66)	5.2
Lewinsohn et al, 1998	316	534	0.59 (0.53−0.65)	5.4
Total			0.37 (0.26−0.48)	63.4
Heterogeneity: $\chi^2_{11} = 301$ ($P < .001$), $I^2 = 96\%$				
Informant source: Parent				
Coker et al, 2009	58	366	0.16 (0.11−0.22)	5.4
Chavira et al, 2004	3	8	0.38 (0.04−0.85)	2.4
Buckner et al, 1997	4	10	0.40 (0.07−0.83)	2.7
Total			0.25 (0.00−0.50)	10.5
Heterogeneity: $\chi^2_{2} = 3.92$ ($P = .14$), $I^2 = 49\%$				
Informant source: Parent and child				
Zhong et al, 2013	5	86	0.06 (0.01−0.16)	5.3
Tanielian et al, 2009	53	183	0.29 (0.20−0.39)	5.2
Merikangas et al, 2011	292	741	0.39 (0.34−0.45)	5.4
Wu et al, 1999	37	80	0.46 (0.31−0.62)	4.8
Wu et al, 2001	131	206	0.64 (0.54−0.73)	5.2
Total			0.37 (0. 10−0.63)	26.1
Heterogeneity: $\chi^2_{4} = 218.8$ ($P < .001$), $I^2 = 98\%$				
Total			0.36 (0.26−0.46)	100.0

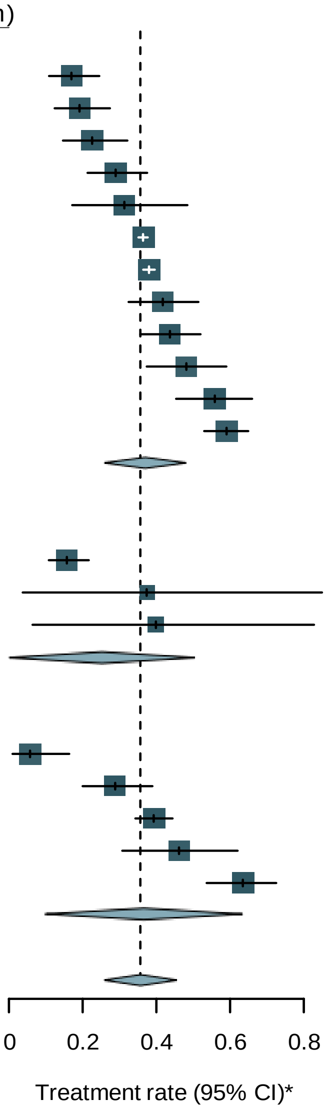

Heterogeneity: $\chi^2_{19} = 645.13$ ($P < .001$), $I^2 = 97\%$
Test for subgroup differences: $\chi^2_{2} = 1.40$ ($P = .50$)

*95% CI adjusted by Bonferroni correction

eFigure 27. Treatment rates for depressive disorders by informant sources

© 2023 Wang S et al. *JAMA Network Open.*

Study	Events, No.	Total, No.	Treatment rate (95% CI)*	Weight, % (Random)
Informant source: Child				
Borges et al, 2008	122	917	0.13 (0.10−0.17)	11.9
Essau et al, 2005	35	192	0.18 (0.11−0.27)	11.6
Wagner et al, 2017	148	542	0.27 (0.22−0.33)	11.8
Merikangas et al, 2010	21	65	0.32 (0.18−0.50)	10.5
Niermann et al, 2021	41	125	0.33 (0.22−0.45)	11.1
Total			0.24 (0. 13−0.35)	56.8
Heterogeneity: $\chi^2_4 = 59.68$ ($P < .001$), $I^2 = 93\%$				
Informant source: Parent			0.31 (0.17−0.49)	10.6
Chavira et al, 2004	21	67	0.49 (0.27−0.71)	9.6
Buckner et al, 1997	20	41	0.64 (0.52−0.75)	11.2
Reardon et al, 2020	89	138	0.48 (0.21−0.76)	31.3
Total				
Heterogeneity: $\chi^2_2 = 22.83$ ($P < .001$), $I^2 = 91\%$				
Informant source: Parent and child				
Merikangas et al, 2011	268	1506	0.18 (0.15−0.21)	11.9
Total			0.31(0. 16−0.46)	100.0

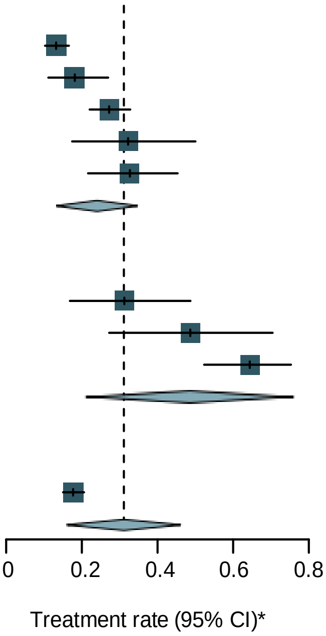

Heterogeneity: $\chi^2_8 = 205.69$ ($P < .001$), $I^2 = 96\%$
Test for subgroup differences: $\chi^2_2 = 11.54$ ($P = .003$)

*95% CI adjusted by Bonferroni correction

eFigure 28. Treatment rates for anxiety disorders by informant sources

Study	Events, No.	Total, No.	Treatment rate (95% CI)*	Weight, % (Random)
Informant source: Parent				
Bussing et al, 2003	89	389	0.23 (0.17−0.29)	10.1
Sawyer et al, 2004	112	398	0.28 (0.22−0.35)	10.1
Coker et al, 2009	149	513	0.29 (0.24−0.35)	10.1
Heiervang et al, 2007	61	82	0.74 (0.59−0.86)	9.8
Bussing et al, 2011	133	168	0.79 (0.69−0.87)	10.1
Chavira et al, 2004	23	29	0.79 (0.53−0.95)	9.4
Total			0.52 (0.20−0.83)	59.7
Heterogeneity: $\chi^2_5 = 348.04$ ($P < .001$), $I^2 = 99\%$				
Informant source: Child			0.48 (0.41−0.54)	10.1
Merikangas et al, 2010	218	457	0.60 (0.46−0.72)	9.9
Bird et al, 2008	65	109	0.98 (0.97−0.98)	10.2
Locke et al, 2017	9192	9412	0.68 (0.26−1.00)	30.2
Total				
Heterogeneity: $\chi^2_2 = 519.87$ ($P < .001$), $I^2 = 100\%$				
Informant source: Parent and child				
Merikangas et al, 2011	369	617	0.60 (0.54−0.65)	10.1
Total			0.58 (0.35−0.80)	100.0

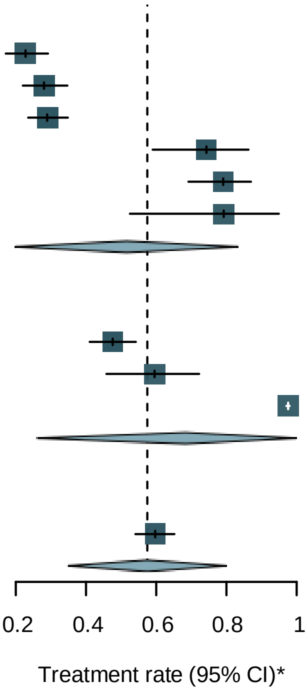

Heterogeneity: $\chi^2_9 = 4192.28$ ($P < .001$), $I^2 = 100\%$
Test for subgroup differences: $\chi^2_2 = 0.82$ ($P = .66$)

*95% CI adjusted by Bonferroni correction

eFigure 29. Treatment rates for ADHD by informant sources

© 2023 Wang S et al. *JAMA Network Open.*

Study	Events, No.	Total, No.	Treatment rate (95% CI)*	Weight, % (Random)
Informant source: Child				
Wagner et al, 2017	51	130	0.39 (0.28−0.52)	12.6
Georgiades et al, 2019	96	194	0.49 (0.39−0.60)	12.8
Total			0.45 (0.30−0.59)	25.5
Heterogeneity: χ^2_1 = 3.37 (*P* = .07), I^2 = 70%				
Informant source: Parent				
Coker et al, 2009	231	838	0.28 (0.23−0.32)	13.3
Buckner et al,1997	6	16	0.38 (0.10−0.73)	9.2
Heievang et al, 2007	65	157	0.41 (0.31−0.53)	12.7
Georgiades et al, 2019	499	714	0.70 (0.65−0.75)	13.2
Total			0.45 (0. 18−0.72)	48.4
Heterogeneity: χ^2_3 = 338.09 (*P* < .001), I^2 = 99%				
Informant source: Parent and child				
Merikangas et al, 2011	442	974	0.45 (0.41−0.50)	13.2
Wu et al, 1999	108	132	0.82 (0.71−0.90)	12.9
Total			0.63 (0.13−1.00)	26.1
Heterogeneity: χ^2_1 = 96.11 (*P* < .001), I^2 = 99%				
Total			0.49 (0.31−0.68)	100.0

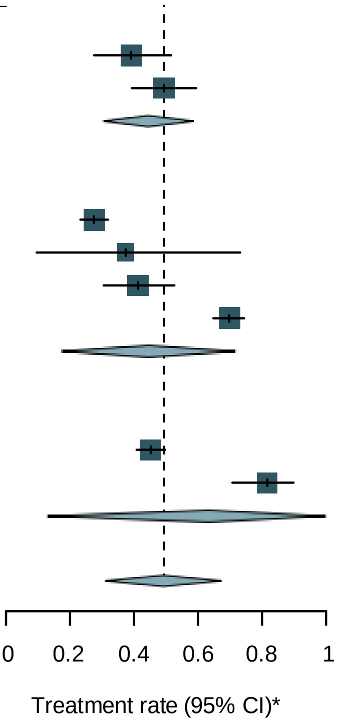

Heterogeneity: χ^2_7 = 449.90 (*P* < .001), I^2 = 98%
Test for subgroup differences: χ^2_2 = 1.01 (*P* = .60)

*95% CI adjusted by Bonferroni correction

eFigure 30. Treatment rates for behavior disorders by informant sources

© 2023 Wang S et al. *JAMA Network Open.*

Outcomes	Study, No.	Events, No.	Total, No.	Treatment rate (95% CI)
School-based counsel				
Any mental disorder	6	1220	6287	0.23 (0.06−0.39)
Depressive disorders	3	2022	8335	0.23 (0.14−0.33)
Anxiety disorders	1	77	1506	0.05 (0.04−0.07)
ADHD	2	301	1015	0.27 (0.00−0.56)
Behavior disorders	2	651	1688	0.41 (0.00−0.88)
Psychiatric treatment				
Any mental disorder	8	1334	7784	0.18 (0.11−0.25)
Depressive disorders	3	2560	8335	0.36 (0.24−0.48)
Anxiety disorders	3	173	1823	0.16 (0.02−0.31)
ADHD	5	1213	10677	0.36 (0.03−0.69)
Behavior disorders	3	572	1845	0.33 (0.24−0.42)
General medical care				
Any mental disorder	6	894	4234	0.17 (0.00−0.34)
Depressive disorders	5	4949	19708	0.24 (0.14−0.37)
Anxiety disorders	1	23	125	0.18 (0.10−0.30)
ADHD	1	66	168	0.39 (0.29−0.50)
Behavior disorders	2	585	1688	0.36 (0.18−0.53)
Medication				
Any mental disorder	2	235	1896	0.12 (0.06−0.18)
Depressive disorders	7	3507	20312	0.17 (0.12−0.23)
Anxiety disorders	3	13	330	0.03 (0.00−0.06)
ADHD	3	124	536	0.41 (0.00−0.93)

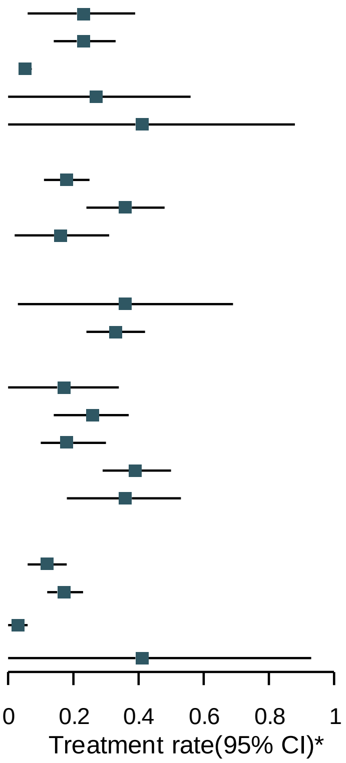

*95% CI adjusted by Bonferroni correction

eFigure 31. Subgroup by health service type

© 2023 Wang S et al. *JAMA Network Open.*

Study	Events, No.	Total, No.	Treatment rate (95% CI)*	Weight, % (Random)
Service: School-based counsel				
Borges et al, 2008	55	1204	0.05 (0.03−0.06)	4.6
Merikangas et al, 2011	371	2899	0.13 (0.11−0.15)	4.6
Vicente et al, 2012	72	329	0.22 (0.16−0.29)	4.4
Canino et al, 2004	73	304	0.24 (0.18−0.31)	4.4
Leaf et al, 1996	104	415	0.25 (0.19−0.31)	4.5
Georgiadeset al, 2019	545	1136	0.48 (0.44−0.52)	4.5
Total			0.23 (0.06−0.39)	27.1
Heterogeneity: χ^2_5 = 820.33 (*P* < .001), I^2 = 99%				
Service: Psychiatric treatment				
Sawyer et al, 2018	71	846	0.08 (0.06−0.11)	4.6
Borges et al, 2008	110	1204	0.09 (0.07−0.12)	4.6
Canino et al, 2004	50	304	0.16 (0.11−0.23)	4.5
Merikangas et al, 2011	488	2899	0.17 (0.15−0.19)	4.6
Vicente et al, 2012	58	329	0.18 (0.12−0.24)	4.5
Fatoriet al, 2019	124	651	0.19 (0.15−0.24)	4.5
Leaf et al, 1996	104	415	0.25 (0.19−0.31)	4.5
Georgiades et al, 2019	329	1136	0.29 (0.25−0.33)	4.6
Total			0.18 (0.11−0.25)	36.3
Heterogeneity: χ^2_7 = 243.76 (*P* < .001), I^2 = 97%				
Service: General medical care				
Canino et al, 2004	9	304	0.03 (0.01−0.07)	4.6
Vicente et al, 2012	21	329	0.06 (0.03−0.11)	4.6
Leaf et al, 1996	37	415	0.09 (0.05−0.13)	4.6
Borges et al, 2008	116	1204	0.10 (0.07−0.12)	4.6
Georgiades et al, 2019	402	1136	0.35 (0.31−0.39)	4.6
Sawyer et al, 2018	309	846	0.37 (0.32−0.41)	4.5
Total			0.17 (0.00−0.34)	27.4
Heterogeneity: χ^2_5 = 605.52 (*P* < .001), I^2 = 99%				
Service: Medication				
Fatori et al, 2019	62	651	0.10 (0.07−0.13)	4.6
Wagner et al, 2017	173	1245	0.14 (0.11−0.17)	4.6
Total			0.12 (0.06−0.18)	9.2
Heterogeneity: χ^2_1 = 8.37 (*P* = .004), I^2 = 88%				
Total			0.18 (0.11−0.25)	100.0

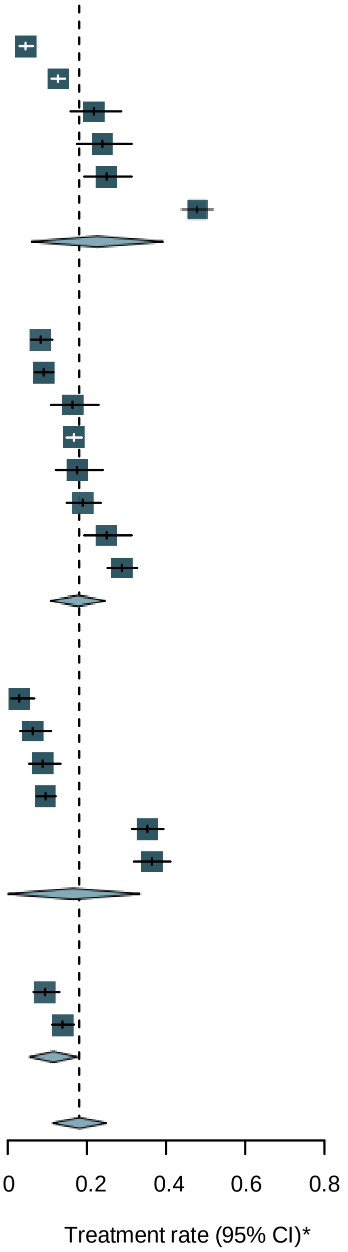

Heterogeneity: χ^2_{21} = 1704.05 (*P* < .001), I^2 = 99%

Test for subgroup differences: χ^2_3 = 4.96 (*P* = .17)

*95% CI adjusted by Bonferroni correction

eFigure 32. Treatment rates for any mental disorder by health service types

© 2023 Wang S et al. *JAMA Network Open.*

Study	Events, No.	Total, No.	Treatment rate (95% CI)*	Weight, % (Random)
Service: School-based counsel				
Lewinsohn et al, 1998	94	534	0.18 (0.13–0.23)	5.8
Cummings et al, 2011	1874	7618	0.25 (0.23–0.26)	6.0
Tanielian et al, 2009	54	183	0.30 (0.21–0.40)	5.2
Total			0.23 (0.14–0.33)	17.0
Heterogeneity: χ^2_2 = 19.14 (P < .001), I^2 = 90%				
Service: Psychiatric treatment				
Cummings et al, 2011	2263	7618	0.30 (0.28–0.31)	6.0
Tanielian et al, 2009	64	183	0.35 (0.26–0.45)	5.2
Lewinsohn et al, 1998	233	534	0.44 (0.38–0.50)	5.7
Total			0.36 (0.24–0.48)	16.8
Heterogeneity: χ^2_2 = 41.33 (P < .001), I^2 = 95%				
Service: General medical care				
Cummings et al, 2011	868	7618	0.11 (0.10–0.12)	6.0
Tanielian et al, 2009	44	183	0.24 (0.16–0.34)	5.3
Meredith et al, 2009	53	184	0.29 (0.20–0.39)	5.3
Stiffman et al, 1988	53	170	0.31 (0.22–0.42)	5.2
Zhang et al, 2021	3931	11553	0.34 (0.33–0.35)	6.0
Total			0.26 (0. 14–0.37)	27.7
Heterogeneity: χ^2_4 = 1583.06 (P < .001), I^2 = 100%				
Service: Medication				
Lewinsohn et al, 1999	46	534	0.09 (0.06–0.13)	5.9
Cummings et al, 2011	1265	7618	0.17 (0.15–0.18)	6.0
Meredith et al, 2009	31	184	0.17 (0.10–0.26)	5.5
Zhang et al, 2021	2075	11553	0.18 (0.17–0.19)	6.0
Stiffman et al, 1988	31	170	0.18 (0.11–0.28)	5.4
Tanielian et al, 2009	37	183	0.20 (0.13–0.30)	5.4
Flament et al, 2001	22	70	0.31 (0.17–0.49)	4.3
Total			0.17 (0.12–0.23)	38.4
Heterogeneity: χ^2_6 = 63.88 (P < .001), I^2 = 91%				
Total			0.24 (0.18–0.30)	100.0

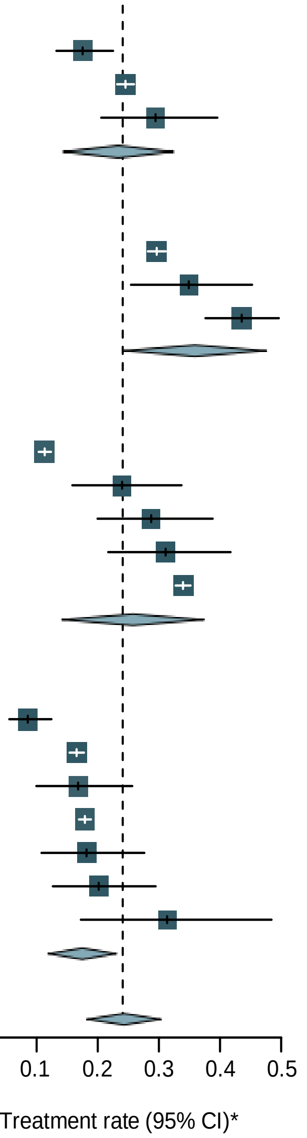

Heterogeneity: χ^2_{17} = 2338.78 (P < .001), I^2 = 99%
Test for subgroup differences: χ^2_3 = 16.83 (P < .001)

*95% CI adjusted by Bonferroni correction

eFigure 33. Treatment rates for depressive disorders by health service types

© 2023 Wang S et al. *JAMA Network Open.*

Study	Events, No.	Total, No.	Treatment rate (95% CI)*	Weight, % (Random)
Service: School-based counsel				
Merikangas et al, 2011	77	1506	0.05 (0.04−0.07)	13.7
Service: Psychiatric treatment				
Merikangas et al, 2011	107	1506	0.07 (0.05−0.09)	13.7
Essau et al, 2005	35	192	0.18 (0.11−0.27)	12.2
Niermann et al, 2021	31	125	0.25 (0.15−0.37)	11.0
Total			0.16 (0.02−0.31)	36.8
Heterogeneity: χ^2_2 = 34.16 (P < .001), I^2 = 94%				
Service: General medical care				
Niermann et al, 2021	23	125	0.18 (0.10−0.30)	11.4
Service: Medication				
Niermann et al, 2021	3	125	0.02 (0.00−0.09)	13.3
Reardon et al, 2020	4	138	0.03 (0.00−0.09)	13.3
Chavira et al, 2004	6	67	0.09 (0.02−0.23)	11.4
Total			0.03 (0.00−0.06)	38.1
Heterogeneity: χ^2_2 = 3.1 (P = .21), I^2 = 35%				
Total			0.10 (0.03−0.18)	100.0

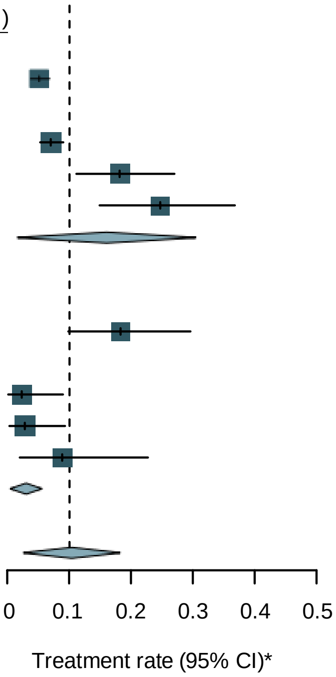

Heterogeneity: χ^2_7 = 73.30 (P < .001), I^2 = 90%

Test for subgroup differences: χ^2_3 = 23.47 (P < .001)

*95% CI adjusted by Bonferroni correction

eFigure 34. Treatment rates for anxiety disorders by health service types

© 2023 Wang S et al. *JAMA Network Open.*

Study	Events, No.	Total, No.	Treatment rate (95% CI)*	Weight, % (Random)
**Service: School-based counsel**				
Sawyer et al, 2004	68	398	0.17 (0.12−0.23)	9.2
Merikangas et al, 2011	233	617	0.38 (0.32−0.43)	9.2
Total			0.27 (0.00−0.56)	18.5
Heterogeneity: χ^2_1 = 58.03 (P < .001), I^2 = 98%				
**Service: Psychiatric treatment**				
Locke et al, 2017	774	9412	0.08 (0.07−0.09)	9.3
Sawyer et al, 2004	50	398	0.13 (0.08−0.18)	9.3
Merikangas et al, 2011	251	617	0.41 (0.35−0.46)	9.2
Bussing et al, 2011	77	168	0.46 (0.35−0.57)	9.1
Heiervang et al, 2007	61	82	0.74 (0.59−0.86)	8.9
Total			0.36 (0.03−0.69)	45.8
Heterogeneity: χ^2_4 = 542.68 (P < .001), I^2 = 99%				
**Service: General medical care**				
Bussing et al, 2011	66	168	0.39 (0.29−0.50)	9.1
**Service: Medication**				
Sawyer et al, 2004	73	398	0.18 (0.13−0.24)	9.2
Bird et al, 2008	28	109	0.26 (0.15−0.39)	9.0
Chavira et al, 2004	23	29	0.79 (0.53−0.95)	8.4
Total			0.41 (0.00−0.93)	26.7
Heterogeneity: χ^2_2 = 62.19 (P < .001), I^2 = 97%				
Total			0.36 (0.16−0.55)	100.0

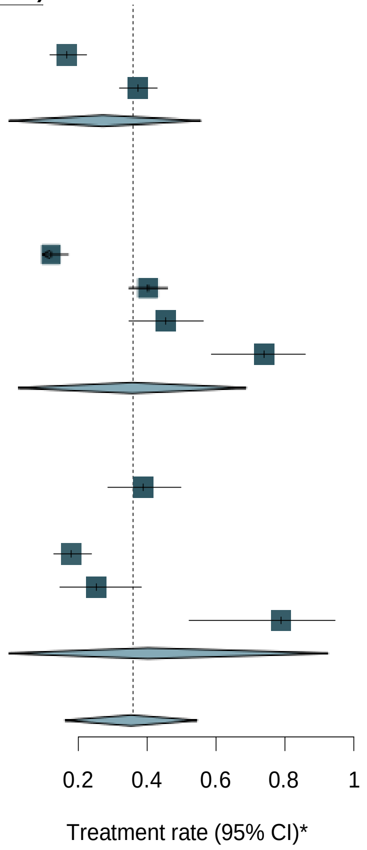

Heterogeneity: χ^2_{10} = 938.10 (P < .001), I^2 = 99%

Test for subgroup differences: χ^2_3 = 1.21 (P = .75)

*95% CI adjusted by Bonferroni correction

eFigure 35. Treatment rates for ADHD by health service types

© 2023 Wang S et al. *JAMA Network Open.*

Study	Events, No.	Total, No.	Treatment rate (95% CI)*	Weight, % (Random)
**Service: School-based counsel**				
Merikangas et al, 2011	236	974	0.24 (0.21−0.28)	14.5
Georgiades et al, 2019	415	714	0.58 (0.53−0.63)	14.4
Total			0.41 (0.00−0.88)	28.9
Heterogeneity: χ^2_1 = 217 (*P* < .001), I^2 = 100%				
**Service: Psychiatric treatment**				
Merikangas et al, 2011	286	974	0.29 (0.25−0.34)	14.5
Georgiades et al, 2019	221	714	0.31 (0.26−0.36)	14.4
Heievang et al, 2007	65	157	0.41 (0.31−0.53)	13.2
Total			0.33 (0.24−0.42)	42.2
Heterogeneity: χ^2_2 = 8.25 (*P* = .02), I^2 = 76%				
**Service: General medical care**				
Merikangas et al, 2011	286	974	0.29 (0.25−0.34)	14.5
Georgiades et al, 2019	299	714	0.42 (0.37−0.47)	14.4
Total			0.36 (0.18−0.53)	28.9
Heterogeneity: χ^2_1 = 28.27 (*P* < .001), I^2 = 96%				
Total			0.36 (0.24−0.49)	100.0

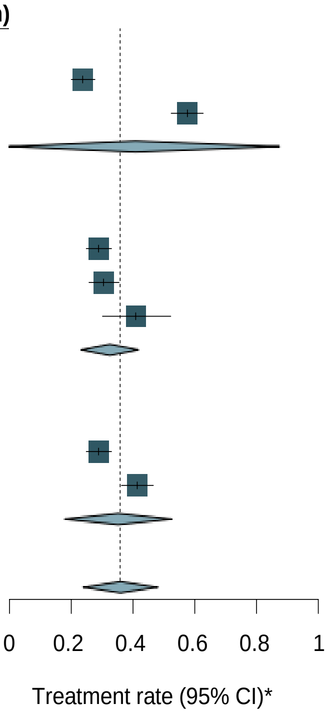

Heterogeneity: χ^2_6 = 266.19 (*P* < .001), I^2 = 98%

Test for subgroup differences: χ^2_2 = 0.32 (*P* = .85)

*95% CI adjusted by Bonferroni correction

eFigure 36. Treatment rates for behavior disorders by health service types

Outcomes	Study, No.	Events, No.	Patients, No.	Treatment rate (95% CI)*
School				
Any mental disorder	4	847	1923	0.41 (0.33−0.49)
Depressive disorders	7	643	1867	0.31 (0.15−0.47)
Anxiety disorders	3	272	872	0.37 (0.00−0.75)
ADHD	4	432	1152	0.51 (0.10−0.92)
Behavior disorders	3	347	1125	0.35 (0.23−0.48)
Community				
Any mental disorder	11	3336	8754	0.37 (0.22−0.51)
Depressive disorders	8	7761	20799	0.36 (0.19−0.54)
Anxiety disorders	4	452	2613	0.23 (0.09−0.37)
ADHD	4	764	1581	0.49 (0.28−0.69)
Behavior disorders	3	1049	1820	0.66 (0.36−0.95)
Health care institution				
Any mental disorder	2	67	228	0.40 (0.00−0.95)
Depressive disorders	5	245	555	0.43 (0.26−0.60)
Anxiety disorders	2	41	108	0.39 (0.15−0.63)
ADHD	2	9215	9441	0.90 (0.65−1.00)
Behavior disorders	1	6	16	0.38 (0.10−0.73)

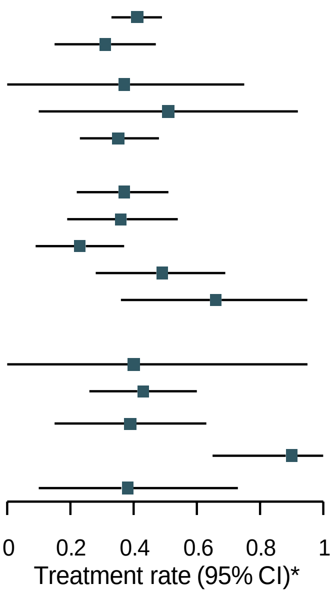

*95% CI adjusted by Bonferroni correction

eFigure 37. Subgroup by sample origin population

© 2023 Wang S et al. *JAMA Network Open.*

Study	Events, No.	Total, No.	Treatment rate (95% CI)*	Weight, % (Random)
Sample origin: School				
Angold et al, 2002	70	194	0.36 (0.27−0.46)	5.8
Cuffe et al, 2001	78	212	0.37 (0.28−0.46)	5.8
Daeem et al, 2019	108	272	0.40 (0.32−0.48)	5.9
Wagner et al, 2017	591	1245	0.47 (0.44−0.51)	6.0
Total			0.41 (0.33−0.49)	23.5
Heterogeneity: $\chi^2_3 = 18.11$ ($P < .001$), $I^2 = 83\%$				
Sample origin: Community				
Maalouf et al, 2016	8	133	0.06 (0.02−0.14)	6.0
Borges et al, 2008	165	1204	0.14 (0.11−0.17)	6.1
Fatori et al, 2019	124	651	0.19 (0.15−0.24)	6.0
Canino et al, 2004	108	304	0.36 (0.28−0.44)	5.9
Merikangas et al, 2011	1049	2899	0.36 (0.34−0.39)	6.1
Vicente et al, 2012	133	329	0.40 (0.33−0.48)	5.9
Gómez−Beneyto et al, 1994	49	114	0.43 (0.30−0.56)	5.6
Leaf et al, 1996	192	415	0.46 (0.39−0.53)	5.9
Merikangas et al, 2010	366	723	0.51 (0.45−0.56)	6.0
Sawyer et al, 2018	448	846	0.53 (0.48−0.58)	6.0
Georgiades et al, 2019	694	1136	0.61 (0.57−0.65)	6.0
Total			0.37 (0.22−0.51)	65.6
Heterogeneity: $\chi^2_{10} = 1289.41$ ($P < .001$), $I^2 = 99\%$				
Sample origin: Health care institution				
Zwaanswijk et al, 2005	38	180	0.21 (0.13−0.31)	5.9
Buckner et al, 1997	29	48	0.60 (0.40−0.79)	5.0
Total			0.40 (0.00−0.95)	10.9
Heterogeneity: $\chi^2_1 = 26.15$ ($P < .001$), $I^2 = 96\%$				
Total			0.38 (0.27−0.48)	100.0

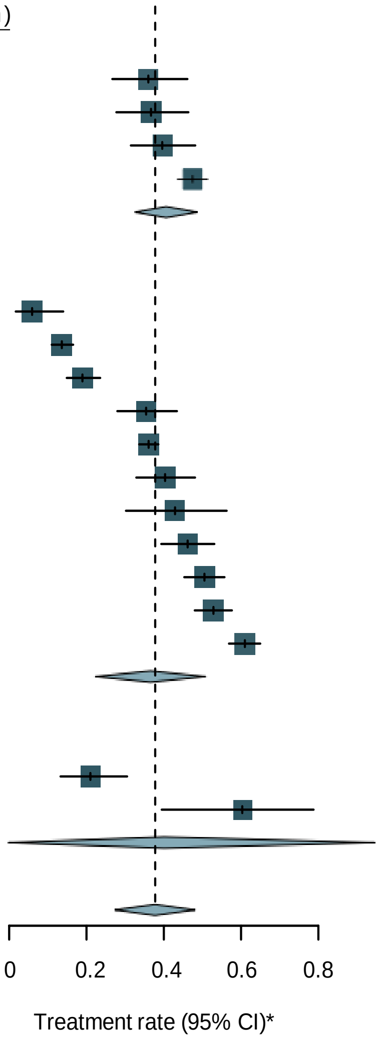

Heterogeneity: $\chi^2_{16} = 1416.09$ ($P < .001$), $I^2 = 99\%$

Test for subgroup differences: $\chi^2_2 = 0.41$ ($P = .81$)

*95% CI adjusted by Bonferroni correction

eFigure 38. Treatment rates for any mental disorder by the sample origin population

© 2023 Wang S et al. *JAMA Network Open.*

Study	Events, No.	Total, No.	Treatment rate (95% CI)*	Weight, % (Random)
**Sample origin: School**				
Coker et al, 2009	58	366	0.16 (0.11−0.22)	5.4
Magklara et al, 2015	42	246	0.17 (0.11−0.25)	5.4
Essau et al, 2005	42	185	0.23 (0.15−0.32)	5.3
Gould et al, 2009	73	251	0.29 (0.21−0.38)	5.3
Flament et al, 2001	22	70	0.31 (0.17−0.49)	4.8
Wagner et al, 2017	90	215	0.42 (0.33−0.52)	5.2
Lewinsohn et al, 1998	316	534	0.59 (0.53−0.65)	5.4
Total			0.31 (0.15−0.47)	36.8
Heterogeneity: χ^2_6 = 290.1 (P < .001), I^2 = 98%				
**Sample origin: Community**				
Zhong et al, 2013	5	86	0.06 (0.01−0.16)	5.3
Borges et al, 2008	43	223	0.19 (0.13−0.28)	5.3
Zhang et al, 2021	4221	11553	0.37 (0.35−0.38)	5.5
Cummings et al, 2011	2904	7618	0.38 (0.37−0.40)	5.5
Merikangas et al, 2011	292	741	0.39 (0.34−0.45)	5.4
Merikangas et al, 2010	128	292	0.44 (0.36−0.52)	5.3
Wu et al, 1999	37	80	0.46 (0.31−0.62)	4.8
Wu et al, 2001	131	206	0.64 (0.54−0.73)	5.2
Total			0.36 (0.19−0.54)	42.4
Heterogeneity: χ^2_7 = 275.45 (P < .001), I^2 = 97%				
**Sample origin: Health care institution**				
Tanielian et al, 2009	53	183	0.29 (0.20−0.39)	5.2
Chavira et al, 2004	3	8	0.38 (0.04−0.85)	2.4
Buckner et al, 1997	4	10	0.40 (0.07−0.83)	2.7
Stiffman et al, 1988	82	170	0.48 (0.38−0.59)	5.2
Meredith et al, 2009	103	184	0.56 (0.46−0.66)	5.2
Total			0.43 (0.26−0.60)	20.7
Heterogeneity: χ^2_4 = 32.1 (P < .001), I^2 = 88%				
Total			0.36 (0.26−0.46)	100.0

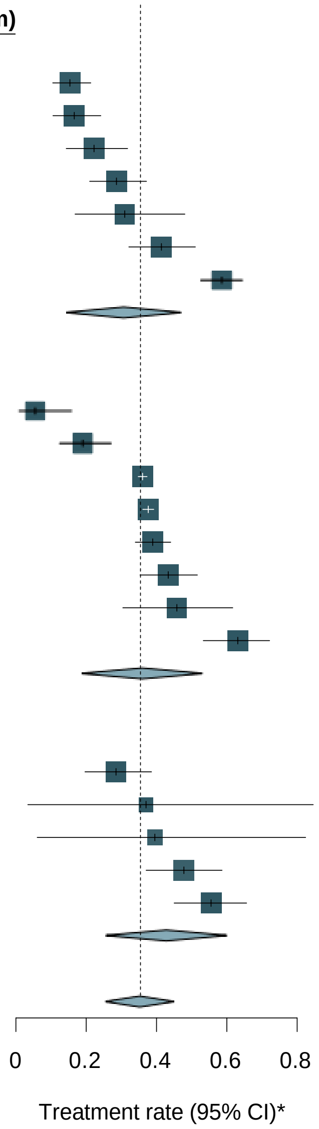

Heterogeneity: χ^2_{19} = 645.13 (P < .001), I^2 = 97%

Test for subgroup differences: χ^2_2 = 2.06 (P = .36)

*95% CI adjusted by Bonferroni correction

eFigure 39. Treatment rates for depressive disorders by the sample origin population

© 2023 Wang S et al. *JAMA Network Open.*

Study	Events, No.	Total, No.	Treatment rate (95% CI)*	Weight, % (Random)
**Sample origin: School**				
Essau et al, 2005	35	192	0.18 (0.11−0.27)	11.6
Wagner et al, 2017	148	542	0.27 (0.22−0.33)	11.8
Reardon et al, 2020	89	138	0.64 (0.52−0.75)	11.2
Total			0.37 (0.00−0.75)	34.5
Heterogeneity: $\chi^2_2 = 91.46$ ($P < .001$), $I^2 = 98\%$				
**Sample origin: Community**				
Borges et al, 2008	122	917	0.13 (0.10−0.17)	11.9
Merikangas et al, 2011	268	1506	0.18 (0.15−0.21)	11.9
Merikangas et al, 2010	21	65	0.32 (0.18−0.50)	10.5
Niermann et al, 2021	41	125	0.33 (0.22−0.45)	11.1
Total			0.23 (0.09−0.37)	45.4
Heterogeneity: $\chi^2_3 = 32.32$ ($P < .001$), $I^2 = 91\%$				
**Sample origin：Health care institution**				
Chavira et al, 2004	21	67	0.31 (0.17−0.49)	10.6
Buckner et al, 1997	20	41	0.49 (0.27−0.71)	9.6
Total			0.39 (0.15−0.63)	20.1
Heterogeneity: $\chi^2_1 = 3.27$ ($P = .07$), $I^2 = 69\%$				
Total			0.31 (0.16−0.46)	100.0

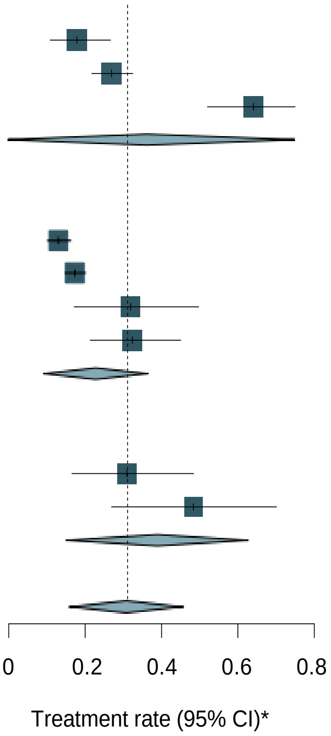

Heterogeneity: $\chi^2_8 = 205.69$ ($P < .001$), $I^2 = 96\%$

Test for subgroup differences: $\chi^2_2 = 3.03$ ($P = .22$)

*95% CI adjusted by Bonferroni correction

eFigure 40. Treatment rates for anxiety disorders by the sample origin population

© 2023 Wang S et al. *JAMA Network Open.*

Study	Events, No.	Total, No.	Treatment rate (95% CI)*	Weight, % (Random)
Sample origin: School				
Bussing et al, 2003	89	389	0.23 (0.17−0.29)	10.1
Coker et al, 2009	149	513	0.29 (0.24−0.35)	10.1
Heiervang et al, 2007	61	82	0.74 (0.59−0.86)	9.8
Bussing et al, 2011	133	168	0.79 (0.69−0.87)	10.1
Total			0.51 (0.10−0.92)	40.2
Heterogeneity: χ^2_3 = 299.93 (P < .001), I^2 = 99%				
Sample origin: Community				
Sawyer et al, 2004	112	398	0.28 (0.22−0.35)	10.1
Merikangas et al, 2010	218	457	0.48 (0.41−0.54)	10.1
Bird et al, 2008	65	109	0.60 (0.46−0.72)	9.9
Merikangas et al, 2011	369	617	0.60 (0.54−0.65)	10.1
Total			0.49 (0.28−0.69)	40.2
Heterogeneity: χ^2_3 = 119.3 (P < .001), I^2 = 97%				
Sample origin: Health care institution				
Chavira et al, 2004	23	29	0.79 (0.53−0.95)	9.4
Locke et al, 2017	9192	9412	0.98 (0.97−0.98)	10.2
Total			0.90 (0.65−1.00)	19.6
Heterogeneity: χ^2_1 = 5.95 (P = .01), I^2 = 83%				
Total			0.58 (0.35−0.80)	100.0

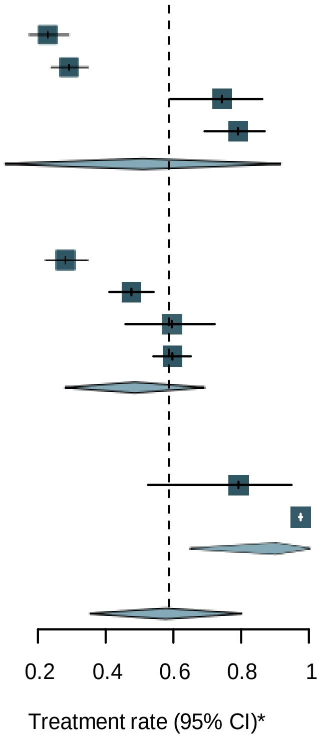

Heterogeneity: χ^2_9 = 4192.28 (P < .001), I^2 = 100%

Test for subgroup differences: χ^2_2 = 13.22 (P = .001)

*95% CI adjusted by Bonferroni correction

eFigure 41. Treatment rates for ADHD by the sample origin population

Study	Events, No.	Total, No.	Treatment rate (95% CI)*	Weight, % (Random)
Sample origin: School				
Coker et al, 2009	231	838	0.28 (0.23−0.32)	15.1
Wagner et al, 2017	51	130	0.39 (0.28−0.52)	14.5
Heievang et al, 2007	65	157	0.41 (0.31−0.53)	14.6
Total			0.35 (0.23−0.48)	44.2
Heterogeneity: χ^2_2 = 15.46 (P < .001), I^2 = 87%				
Sample origin: Community			0.45 (0.41−0.50)	15.1
Merikangas et al, 2011	442	974	0.70 (0.65−0.75)	15.1
Georgiades et al, 2019	499	714	0.82 (0.71−0.90)	14.7
Wu et al, 1999	108	132	0.66 (0.36−0.95)	44.9
Total				
Heterogeneity: χ^2_2 = 159.15 (P < .001), I^2 = 99%				
Sample origin: Health care institution				
Buckner et al,1997	6	16	0.38 (0.10−0.73)	10.9
Total			0.49 (0.28−0.70)	100.0

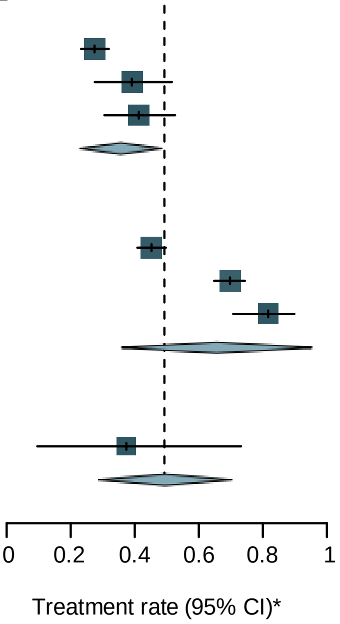

Heterogeneity: χ^2_6 = 449.72 (P < .001), I^2 = 99%

Test for subgroup differences: χ^2_2 = 6.73 (P = .03)

*95% CI adjusted by Bonferroni correction

eFigure 42. Treatment rates for behavior disorders by the sample origin population

© 2023 Wang S et al. *JAMA Network Open.*

Study	Events, No.	Total, No.	Treatment rate (95% CI)*	Weight, % (Random)
Category: Internalising disorders				
Zhong et al, 2013	5	86	0.06 (0.01−0.16)	2.3
Borges et al, 2008	122	917	0.13 (0.10−0.17)	2.3
Coker et al, 2009	58	366	0.16 (0.11−0.22)	2.3
Magklara et al, 2015	42	246	0.17 (0.11−0.25)	2.3
Merikangas et al, 2011	268	1506	0.18 (0.15−0.21)	2.3
Essau et al, 2005	35	192	0.18 (0.11−0.27)	2.2
Borges et al, 2008	43	223	0.19 (0.13−0.28)	2.2
Essau et al, 2005	42	185	0.23 (0.15−0.32)	2.2
Wagner et al, 2017	148	542	0.27 (0.22−0.33)	2.3
Tanielian et al, 2009	53	183	0.29 (0.20−0.39)	2.2
Gould et al, 2009	73	251	0.29 (0.21−0.38)	2.2
Chavira et al, 2004	21	67	0.31 (0.17−0.49)	2.1
Flament et al, 2001	22	70	0.31 (0.17−0.49)	2.1
Merikangas et al, 2010	21	65	0.32 (0.18−0.50)	2.1
Niermann et al, 2021	41	125	0.33 (0.22−0.45)	2.2
Zhang et al, 2021	4221	11553	0.37 (0.35−0.38)	2.3
Chavira et al, 2004	3	8	0.38 (0.04−0.85)	1.3
Cummings et al, 2011	2904	7618	0.38 (0.37−0.40)	2.3
Merikangas et al, 2011	292	741	0.39 (0.34−0.45)	2.3
Buckner et al, 1997	4	10	0.40 (0.07−0.83)	1.5
Wagner et al, 2017	90	215	0.42 (0.33−0.52)	2.2
Merikangas et al, 2010	128	292	0.44 (0.36−0.52)	2.2
Wu et al, 1999	37	80	0.46 (0.31−0.62)	2.1
Stiffman et al, 1988	82	170	0.48 (0.38−0.59)	2.2
Buckner et al, 1997	20	41	0.49 (0.27−0.71)	2.0
Meredith et al, 2009	103	184	0.56 (0.46−0.66)	2.2
Lewinsohn et al, 1998	316	534	0.59 (0.53−0.65)	2.3
Wu et al, 2001	131	206	0.64 (0.54−0.73)	2.2
Reardon et al, 2020	89	138	0.64 (0.52−0.75)	2.2
Total			0.34 (0.26−0.43)	62.7
Heterogeneity: χ^2_{28} = 1405.99 (*P* < .001), I^2 = 98%				
Category: Externalising disorders				
Bussing et al, 2003	89	389	0.23 (0.17−0.29)	2.3
Coker et al, 2009	231	838	0.28 (0.23−0.32)	2.3
Sawyer et al, 2004	112	398	0.28 (0.22−0.35)	2.3
Coker et al, 2009	149	513	0.29 (0.24−0.35)	2.3
Buckner et al,1997	6	16	0.38 (0.10−0.73)	1.7
Wagner et al, 2017	51	130	0.39 (0.28−0.52)	2.2
Heievang et al, 2007	65	157	0.41 (0.31−0.53)	2.2
Merikangas et al, 2011	442	974	0.45 (0.41−0.50)	2.3
Merikangas et al, 2010	218	457	0.48 (0.41−0.54)	2.3
Bird et al, 2008	65	109	0.60 (0.46−0.72)	2.2
Merikangas et al, 2011	369	617	0.60 (0.54−0.65)	2.3
Georgiades et al, 2019	499	714	0.70 (0.65−0.75)	2.3
Heiervang et al, 2007	61	82	0.74 (0.59−0.86)	2.2
Bussing et al, 2011	133	168	0.79 (0.69−0.87)	2.2
Chavira et al, 2004	23	29	0.79 (0.53−0.95)	2.0
Wu et al, 1999	108	132	0.82 (0.71−0.90)	2.2
Locke et al, 2017	9192	9412	0.98 (0.97−0.98)	2.3
Total			0.54 (0.39−0.70)	37.3
Heterogeneity: χ^2_{16} = 7706.48 (*P* < .001), I^2 = 100%				
Total			0.42 (0.33−0.50)	100.0

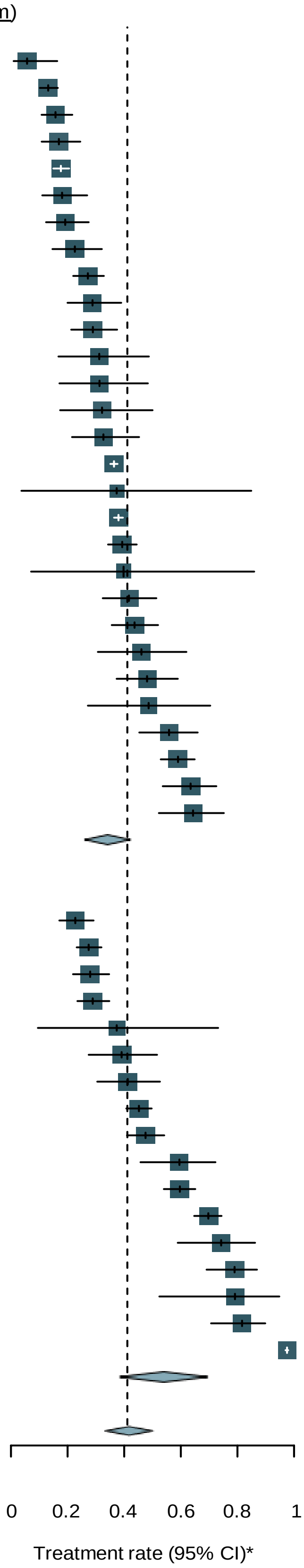

Heterogeneity: χ^2_{45} = 46305.95 (*P* < .001), I^2 = 100%
Test for subgroup differences: χ^2_1 = 9.77 (*P* = .002)

*95% CI adjusted by Bonferroni correction

eFigure 43. Treatment rates for internalizing and externalizing disorders

© 2023 Wang S et al. *JAMA Network Open.*

Study	Treatment rate (95% CI)	Tau^2	Tau	I^2
Omitting Buckner et al, 1997	0.37 (0.29−0.44)	0.0222	0.1492	99%
Omitting Wagner et al, 2017	0.37 (0.29−0.45)	0.0244	0.1561	99%
Omitting Canino et al, 2004	0.38 (0.30−0.46)	0.0250	0.1582	99%
Omitting Angoldet al, 2002	0.38 (0.30−0.46)	0.0250	0.1582	99%
Omitting Borgeset al, 2008	0.39 (0.32−0.47)	0.0208	0.1442	98%
Omitting Zwaanswijket al, 2005	0.39 (0.31−0.46)	0.0232	0.1522	99%
Omitting Cuffeet al, 2001	0.38 (0.30−0.46)	0.0250	0.1582	99%
Omitting Fatoriet al, 2019	0.39 (0.32−0.47)	0.0225	0.1501	99%
Omitting Gómez−Beneytoet al, 1994	0.38 (0.30−0.45)	0.0248	0.1575	99%
Omitting Leaf et al, 1996	0.37 (0.29−0.45)	0.0245	0.1566	99%
Omitting Maalouf et al, 2016	0.40 (0.33−0.46)	0.0178	0.1334	99%
Omitting Merikangas et al, 2010	0.37 (0.29−0.45)	0.0238	0.1544	99%
Omitting Merikangas et al, 2011	0.38 (0.30−0.46)	0.0251	0.1584	99%
Omitting Sawyer et al, 2018	0.37 (0.29−0.44)	0.0234	0.1528	99%
Omitting Vicente et al, 2012	0.38 (0.30−0.46)	0.0250	0.1581	99%
Omitting Daeem et al, 2019	0.38 (0.30−0.46)	0.0250	0.1582	99%
Omitting Georgiades et al, 2019	0.36 (0.29−0.44)	0.0210	0.1448	99%
Total	0.38 (0.30−0.45)	0.0235	0.1531	99%

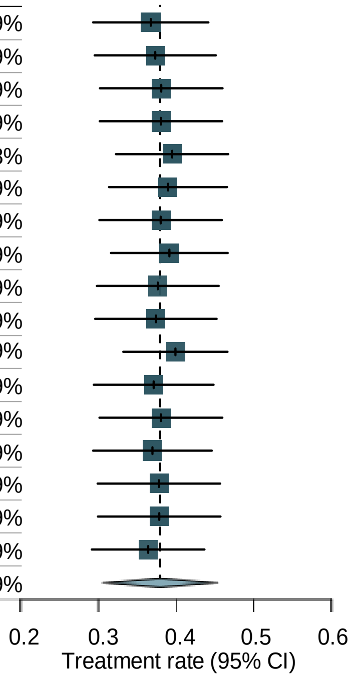

eFigure 44. Sensitivity analysis forest plots for any mental disorder

Study	Treatment rate (95% CI)	Tau^2	Tau	I^2
Omitting Meredith et al, 2009	0.35 (0.28−0.42)	0.0222	0.1490	97%
Omitting Borges et al, 2008	0.37 (0.30−0.44)	0.0230	0.1517	97%
Omitting Lewinsohn et al, 1998	0.34 (0.28−0.41)	0.0212	0.1455	97%
Omitting Gould et al, 2009	0.36 (0.29−0.44)	0.0244	0.1561	97%
Omitting Buckner et al, 1997	0.36 (0.28−0.43)	0.0239	0.1547	97%
Omitting Merikangas et al, 2011	0.36 (0.28−0.43)	0.0246	0.1568	97%
Omitting Zhang et al, 2021	0.36 (0.28−0.43)	0.0247	0.1571	97%
Omitting Cummings et al, 2011	0.36 (0.28−0.43)	0.0246	0.1570	97%
Omitting Stiffman et al, 1988	0.35 (0.28−0.42)	0.0237	0.1539	97%
Omitting Flament et al, 2001	0.36 (0.29−0.43)	0.0244	0.1563	97%
Omitting Tanielian et al, 2009	0.36 (0.29−0.44)	0.0244	0.1561	97%
Omitting Zhong et al, 2013	0.37 (0.31−0.44)	0.0192	0.1384	96%
Omitting Wu et al, 1999	0.35 (0.28−0.43)	0.0240	0.1548	97%
Omitting Magklara et al, 2015	0.37 (0.30−0.44)	0.0225	0.1501	97%
Omitting Wagner et al, 2017	0.35 (0.28−0.43)	0.0244	0.1562	97%
Omitting Coker et al, 2009	0.37 (0.30−0.44)	0.0222	0.1490	97%
Omitting Wu et al, 2001	0.34 (0.28−0.41)	0.0200	0.1414	97%
Omitting Merikangas et al, 2010	0.35 (0.28−0.43)	0.0242	0.1557	97%
Omitting Essau et al, 2005	0.37 (0.29−0.44)	0.0236	0.1537	97%
Omitting Chavira et al, 2004	0.36 (0.29−0.43)	0.0239	0.1546	97%
Total	0.36 (0.29−0.43)	0.0233	0.1525	97%

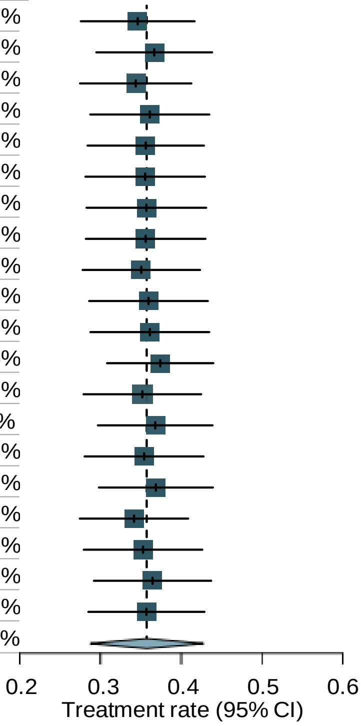

eFigure 45. Sensitivity analysis forest plots for depressive disorders

© 2023 Wang S et al. *JAMA Network Open.*

Study	Treatment rate (95% CI)	Tau^2	Tau	I^2
Omitting Merikangas et al, 2010	0.31 (0.19−0.43)	0.0283	0.1682	97%
Omitting Chavira et al, 2004	0.31 (0.19−0.43)	0.0283	0.1683	97%
Omitting Merikangas et al, 2011	0.33 (0.22−0.45)	0.0257	0.1602	97%
Omitting Niermann et al, 2021	0.31 (0.19−0.43)	0.0285	0.1687	96%
Omitting Reardon et al, 2020	0.26 (0.19−0.33)	0.0090	0.0951	91%
Omitting Wagner et al, 2017	0.32 (0.20−0.44)	0.0285	0.1687	96%
Omitting Borges et al, 2008	0.34 (0.23−0.45)	0.0232	0.1522	96%
Omitting Essau et al, 2005	0.33 (0.21−0.45)	0.0259	0.1610	97%
Omitting Buckner et al, 1997	0.29 (0.18−0.40)	0.0243	0.1559	96%
Total	0.31 (0.21−0.42)	0.0248	0.1573	96%

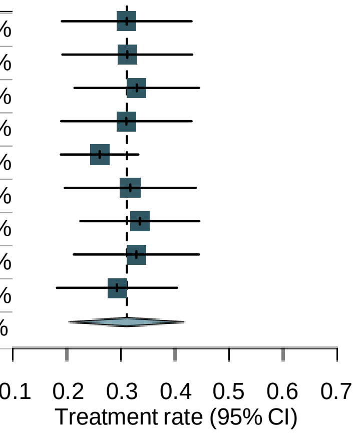

eFigure 46. Sensitivity analysis forest plots for anxiety disorders

Study	Treatment rate (95% CI)	Tau^2	Tau	I^2
Omitting Bird et al, 2008	0.56 (0.39−0.73)	0.0673	0.2594	100%
Omitting Bussing et al, 2003	0.60 (0.45−0.75)	0.0520	0.2281	100%
Omitting Bussing et al, 2011	0.55 (0.38−0.72)	0.0658	0.2566	100%
Omitting Coker et al, 2009	0.59 (0.44−0.75)	0.0572	0.2393	100%
Omitting Heiervang et al, 2007	0.54 (0.38−0.71)	0.0630	0.2510	100%
Omitting Merikangas et al, 2011	0.56 (0.39−0.73)	0.0674	0.2597	100%
Omitting Merikangas et al, 2010	0.57 (0.40−0.74)	0.0666	0.2581	100%
Omitting Locke et al, 2017	0.52 (0.38−0.65)	0.0424	0.2059	98%
Omitting Chavira et al, 2004	0.54 (0.38−0.70)	0.0607	0.2465	100%
Total	0.56 (0.41−0.72)	0.0600	0.2449	100%

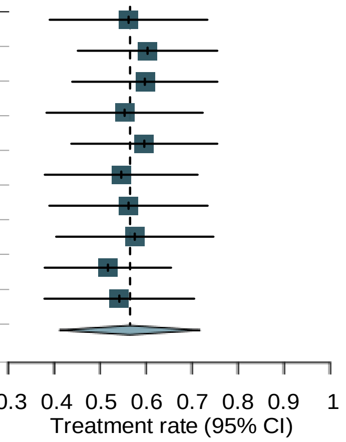

eFigure 47. Sensitivity analysis forest plots for ADHD

Study	Treatment rate (95% CI)	Tau^2	Tau	I^2
Omitting Buckner et al,1997	0.51 (0.34−0.67)	0.0416	0.2040	99%
Omitting Wagner et al, 2017	0.51 (0.34−0.68)	0.0427	0.2067	99%
Omitting Merikangas et al, 2011	0.50 (0.33−0.68)	0.0451	0.2123	99%
Omitting Wu et al, 1999	0.44 (0.32−0.56)	0.0214	0.1461	99%
Omitting Coker et al, 2009	0.53 (0.38−0.69)	0.0329	0.1815	97%
Omitting Heievang et al, 2007	0.51 (0.34−0.68)	0.0437	0.2090	99%
Omitting Georgiades et al, 2019	0.46 (0.30−0.61)	0.0354	0.1880	98%
Total	0.49 (0.35−0.64)	0.0376	0.1938	99%

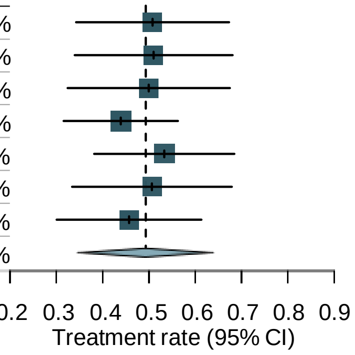

eFigure 48. Sensitivity analysis forest plots for behavior disorders

© 2023 Wang S et al. *JAMA Network Open.*

A. Any mental disorder

Study	Events, No.	Total, No.	Treatment rate (95% CI)	Weight, % (Random)
Canino et al, 2004	108	304	0.36 (0.30−0.41)	10.6
Angold et al, 2002	70	194	0.36 (0.29−0.43)	9.3
Merikangas et al, 2011	1049	2899	0.36 (0.34−0.38)	13.5
Cuffe et al, 2001	78	212	0.37 (0.30−0.44)	9.6
Daeem et al, 2019	108	272	0.40 (0.34−0.46)	10.2
Vicente et al, 2012	133	329	0.40 (0.35−0.46)	10.7
Gómez−Beneyto et al, 1994	49	114	0.43 (0.34−0.53)	7.4
Leaf et al, 1996	192	415	0.46 (0.41−0.51)	11.2
Wagner et al, 2017	591	1245	0.47 (0.45−0.50)	12.9
Buckner et al, 1997	29	48	0.60 (0.45−0.74)	4.6
Total			0.41 (0.38−0.45)	100.0

Heterogeneity: χ^2_9 = 66.22 (*P* < .001), I^2 = 86%

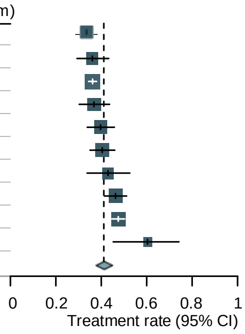

B. Depressive disorders

Study	Events, No.	Total, No.	Treatment rate (95% CI)	Weight, % (Random)
Essau et al, 2005	42	185	0.23 (0.17−0.29)	8.9
Tanielian et al, 2009	53	183	0.29 (0.23−0.36)	8.6
Gould et al, 2009	73	251	0.29 (0.24−0.35)	9.1
Flament et al, 2001	22	70	0.31 (0.21−0.44)	6.5
Zhang et al, 2021	4221	11553	0.37 (0.36−0.37)	10.7
Chavira et al, 2004	3	8	0.38 (0.09−0.76)	1.5
Cummings et al, 2011	2904	7618	0.38 (0.37−0.39)	10.6
Merikangas et al, 2011	292	741	0.39 (0.36−0.43)	10.0
Buckner et al, 1997	4	10	0.40 (0.12−0.74)	1.8
Wagner et al, 2017	90	215	0.42 (0.35−0.49)	8.6
Merikangas et al, 2010	128	292	0.44 (0.38−0.50)	9.1
Wu et al, 1999	37	80	0.46 (0.35−0.58)	6.4
Stiffman et al, 1988	82	170	0.48 (0.41−0.56)	8.2
Total			0.37 (0.32−0.41)	100.0

Heterogeneity: χ^2_{12} = 61.76 (*P* < .001), I^2 = 81%

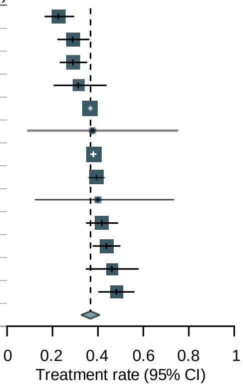

C. Anxiety disorders

Study	Events, No.	Total, No.	Treatment rate (95% CI)	Weight, % (Random)
Essau et al, 2005	35	192	0.18 (0.13−0.24)	20.3
Wagner et al, 2017	148	542	0.27 (0.24−0.31)	21.7
Chavira et al, 2004	21	67	0.31 (0.21−0.44)	14.8
Merikangas et al, 2010	21	65	0.32 (0.21−0.45)	14.5
Niermann et al, 2021	41	125	0.33 (0.25−0.42)	17.6
Buckner et al, 1997	20	41	0.49 (0.33−0.65)	11.2
Total			0.30 (0.23−0.37)	100.0

Heterogeneity: χ^2_5 = 21.04 (*P* < .001), I^2 = 76%

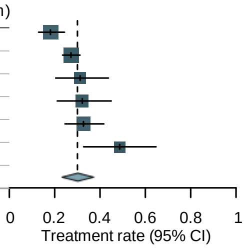

D. Attention deficit hyperactivity disorder (ADHD)

Study	Events, No.	Total, No.	Treatment rate (95% CI)	Weight, % (Random)
Merikangas et al, 2010	218	457	0.48 (0.43−0.52)	18.0
Bird et al, 2008	65	109	0.60 (0.50−0.69)	16.3
Merikangas et al, 2011	369	617	0.60 (0.56−0.64)	18.2
Heiervang et al, 2007	61	82	0.74 (0.64−0.83)	16.2
Bussing et al, 2011	133	168	0.79 (0.72−0.85)	17.6
Chavira et al, 2004	23	29	0.79 (0.60−0.92)	13.6
Total			0.66 (0.56−0.76)	100.0

Heterogeneity: χ^2_5 = 80.10 (*P* < .001), I^2 = 94%

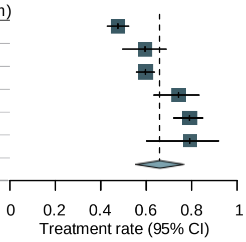

E. Behavior disorders

Study	Events, No.	Total, No.	Treatment rate (95% CI)	Weight, % (Random)
Buckner et al,1997	6	16	0.38 (0.15−0.65)	2.6
Wagner et al, 2017	51	130	0.39 (0.31−0.48)	17.4
Heievang et al, 2007	65	157	0.41 (0.34−0.50)	20.0
Merikangas et al, 2011	442	974	0.45 (0.42−0.49)	60.0
Total			0.43 (0.39−0.47)	100.0

Heterogeneity: χ^2_3 = 2.70 (*P* = .44), I^2 = 0%

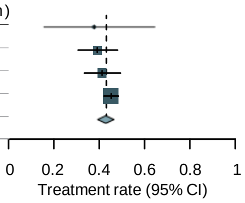

eFigure 49. Sensitivity analysis by excluding studies in which the 95% CI was outside the aggregated 95% CI of all studies

© 2023 Wang S et al. *JAMA Network Open.*

A. Any mental disorder

Study	Events, No.	Total, No.	Treatment rate (95% CI)	Weight, % (Random)
Maalouf et al, 2016	8	133	0.06 (0.03−0.12)	6.8
Borges et al, 2008	165	1204	0.14 (0.12−0.16)	6.8
Fatori et al, 2019	124	651	0.19 (0.16−0.22)	6.8
Zwaanswijk et al, 2005	38	180	0.21 (0.15−0.28)	6.6
Canino et al, 2004	108	304	0.36 (0.30−0.41)	6.7
Angold et al, 2002	70	194	0.36 (0.29−0.43)	6.6
Merikangas et al, 2011	1049	2899	0.36 (0.34−0.38)	6.9
Vicente et al, 2012	133	329	0.40 (0.35−0.46)	6.7
Gómez−Beneyto et al, 1994	49	114	0.43 (0.34−0.53)	6.4
Leaf et al, 1996	192	415	0.46 (0.41−0.51)	6.7
Wagner et al, 2017	591	1245	0.47 (0.45−0.50)	6.8
Merikangas et al, 2010	366	723	0.51 (0.47−0.54)	6.8
Sawyer et al, 2018	448	846	0.53 (0.50−0.56)	6.8
Buckner et al, 1997	29	48	0.60 (0.45−0.74)	5.8
Georgiades et al, 2019	694	1136	0.61 (0.58−0.64)	6.8
Total			0.38 (0.29−0.46)	100.0

Heterogeneity: χ^2_{14} = 1412.75 (*P* < .001), I^2 = 99%

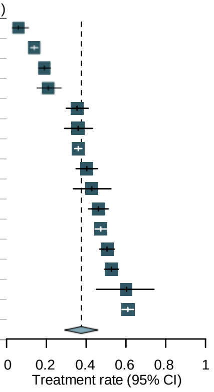

B. Depressive disorders

Study	Events, No.	Total, No.	Treatment rate (95% CI)	Weight, % (Random)
Zhong et al, 2013	5	86	0.06 (0.02−0.13)	6.8
Magklara et al, 2015	42	246	0.17 (0.13−0.22)	6.8
Borges et al, 2008	43	223	0.19 (0.14−0.25)	6.8
Essau et al, 2005	42	185	0.23 (0.17−0.29)	6.7
Tanielian et al, 2009	53	183	0.29 (0.23−0.36)	6.6
Zhang et al, 2021	4221	11553	0.37 (0.36−0.37)	7.0
Chavira et al, 2004	3	8	0.38 (0.09−0.76)	3.0
Cummings et al, 2011	2904	7618	0.38 (0.37−0.39)	7.0
Merikangas et al, 2011	292	741	0.39 (0.36−0.43)	6.9
Buckner et al, 1997	4	10	0.40 (0.12−0.74)	3.4
Wagner et al, 2017	90	215	0.42 (0.35−0.49)	6.6
Merikangas et al, 2010	128	292	0.44 (0.38−0.50)	6.7
Wu et al, 1999	37	80	0.46 (0.35−0.58)	6.1
Stiffman et al, 1988	82	170	0.48 (0.41−0.56)	6.5
Meredith et al, 2009	103	184	0.56 (0.48−0.63)	6.6
Wu et al, 2001	131	206	0.64 (0.57−0.70)	6.6
Total			0.36 (0.28−0.44)	100.0

Heterogeneity: χ^2_{15} = 407.20 (*P* < .001), I^2 = 96%

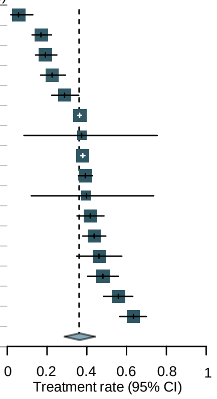

C. Anxiety disorders

Study	Events, No.	Total, No.	Treatment rate (95% CI)	Weight, % (Random)
Borges et al, 2008	122	917	0.13 (0.11−0.16)	11.9
Merikangas et al, 2011	268	1506	0.18 (0.16−0.20)	11.9
Essau et al, 2005	35	192	0.18 (0.13−0.24)	11.6
Wagner et al, 2017	148	542	0.27 (0.24−0.31)	11.8
Chavira et al, 2004	21	67	0.31 (0.21−0.44)	10.6
Merikangas et al, 2010	21	65	0.32 (0.21−0.45)	10.5
Niermann et al, 2021	41	125	0.33 (0.25−0.42)	11.1
Buckner et al, 1997	20	41	0.49 (0.33−0.65)	9.6
Reardon et al, 2020	89	138	0.64 (0.56−0.72)	11.2
Total			0.31 (0.21−0.42)	100.0

Heterogeneity: χ^2_8 = 205.69 (*P* < .001), I^2 = 96%

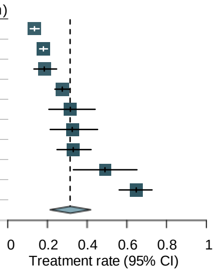

D. Attention deficit hyperactivity disorder(ADHD)

Study	Events, No.	Total, No.	Treatment rate (95% CI)	Weight, % (Random)
Sawyer et al, 2004	112	398	0.28 (0.24−0.33)	14.6
Merikangas et al, 2010	218	457	0.48 (0.43−0.52)	14.6
Bird et al, 2008	65	109	0.60 (0.50−0.69)	14.1
Merikangas et al, 2011	369	617	0.60 (0.56−0.64)	14.6
Heiervang et al, 2007	61	82	0.74 (0.64−0.83)	14.1
Chavira et al, 2004	23	29	0.79 (0.60−0.92)	13.3
Locke et al, 2017	9192	9412	0.98 (0.97−0.98)	14.7
Total			0.64 (0.47−0.81)	100.0

Heterogeneity: χ^2_6 = 1840.92 (*P* < .001), I^2 = 100%

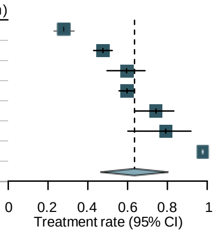

E. Behavior disorders

Study	Events, No.	Total, No.	Treatment rate (95% CI)	Weight, % (Random)
Buckner et al,1997	6	16	0.38 (0.15−0.65)	12.5
Wagner et al, 2017	51	130	0.39 (0.31−0.48)	17.1
Heievang et al, 2007	65	157	0.41 (0.34−0.50)	17.2
Merikangas et al, 2011	442	974	0.45 (0.42−0.49)	17.9
Georgiades et al, 2019	499	714	0.70 (0.66−0.73)	17.9
Wu et al, 1999	108	132	0.82 (0.74−0.88)	17.4
Total			0.53 (0.38−0.69)	100.0

Heterogeneity: χ^2_5 = 199.62 (*P* < .001), I^2 = 97%

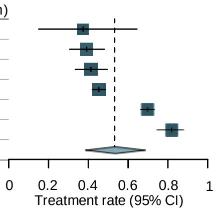

eFigure 50. Sensitivity analysis by excluding studies not reported age ranges

© 2023 Wang S et al. *JAMA Network Open.*

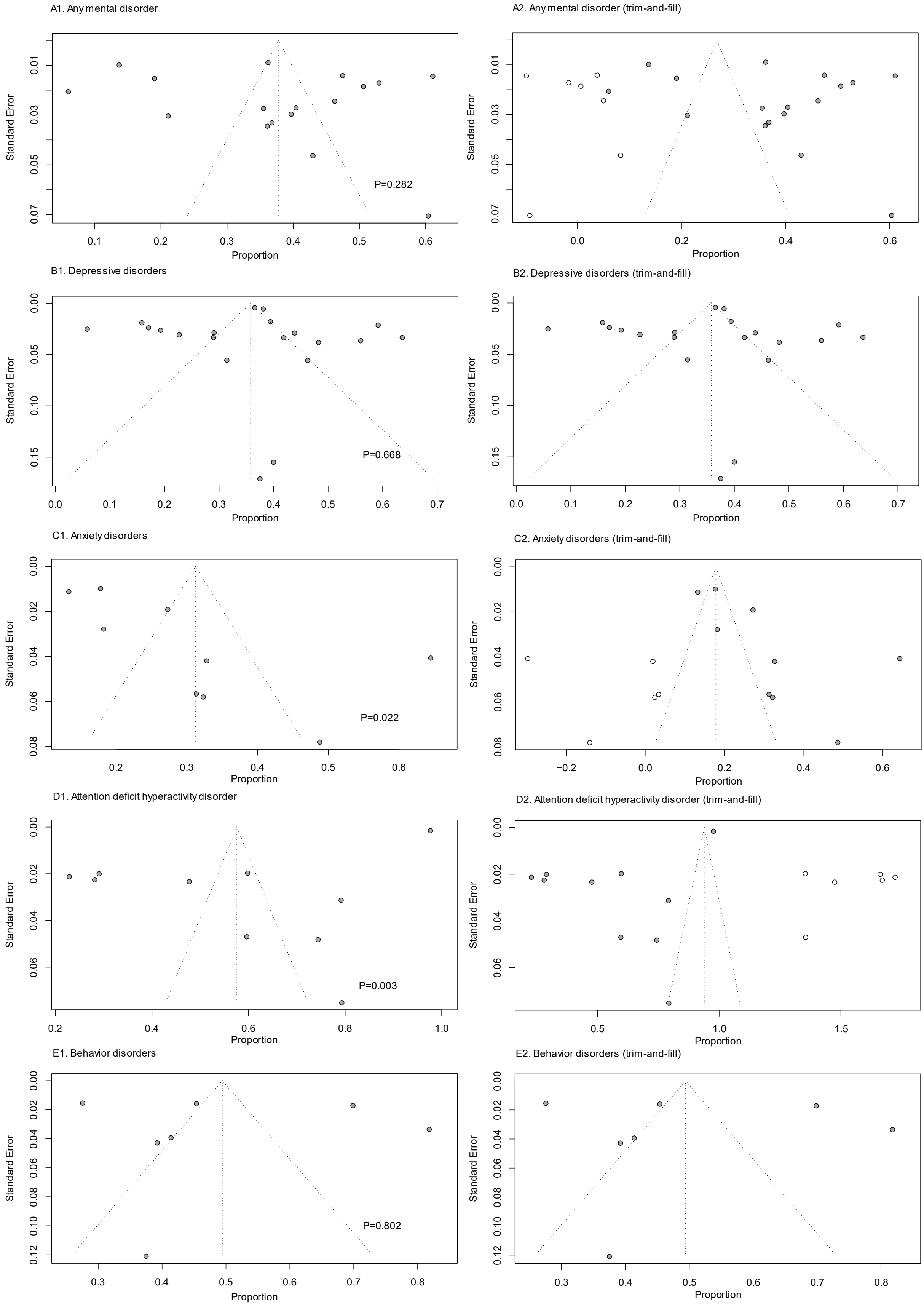

eFigure 51. Funnel plot (left) and trim-and-fill funnel plot (right)

© 2023 Wang S et al. *JAMA Network Open.*

Study	Events, No.	Total, No.	Treatment rate (95% CI)	Weight, % (Random)
Bussing et al,[27] 2003	89	389	0.23 (0.19−0.27)	10.1
Sawyer et al,[50] 2004	112	398	0.28 (0.24−0.33)	10.1
Coker et al,[31] 2009	149	513	0.29 (0.25−0.33)	10.1
Merikangas et al,[14] 2010	218	457	0.48 (0.43−0.52)	10.1
Bird et al,[25] 2008	65	109	0.60 (0.50−0.69)	9.9
Merikangas et al,[47] 2011	369	617	0.60 (0.56−0.64)	10.1
Heiervang et al,[40] 2007	61	82	0.74 (0.64−0.83)	9.8
Bussing et al,[28] 2011	133	168	0.79 (0.72−0.85)	10.1
Chavira et al,[30] 2004	23	29	0.79 (0.60−0.92)	9.4
Locke et al,[43] 2017	9192	9412	0.98 (0.97−0.98)	10.2
Total			0.58 (0.42−0.73)	100.0

Heterogeneity: χ^2_9 = 4192.28 (P < .001), I^2 = 100%

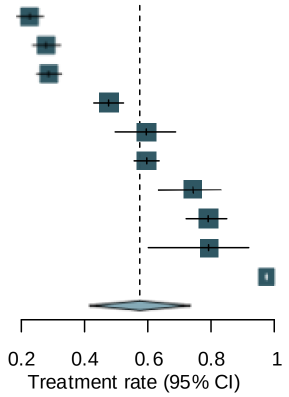

eFigure 52. Treatment Rates for ADHD

**Study**	**Events, No.**	**Total, No.**	**Treatment rate (95% CI)**	**Weight, % (Random)**
Coker et al,[31] 2009	231	838	0.28 (0.25−0.31)	15.1
Buckner et al,[26] 1997	6	16	0.38 (0.15−0.65)	10.9
Wagner et al,[57] 2017	51	130	0.39 (0.31−0.48)	14.5
Heievang et al,[40] 2007	65	157	0.41 (0.34−0.50)	14.6
Merikangas et al,[47] 2011	442	974	0.45 (0.42−0.49)	15.1
Georgiades et al,[37] 2019	499	714	0.70 (0.66−0.73)	15.1
Wu et al,[56] 1999	108	132	0.82 (0.74−0.88)	14.7
Total			0.49 (0.35−0.64)	100.0

Heterogeneity: χ^2_6 = 449.72 (P < .001), I^2 = 99%

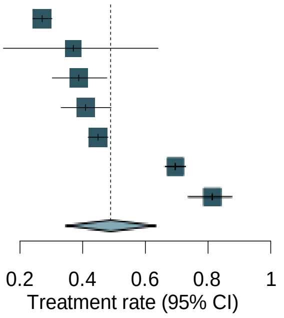

eFigure 53. Treatment Rates for Behavior Disorders

© 2023 Wang S et al. *JAMA Network Open.*

Outcomes	Study, No.	Events, No.	Total, No.	Treatment rate (95% CI)
Children (≤12 years)				
Any mental disorder	5	848	2144	0.37 (0.15−0.59)
Depressive disorders	2	63	452	0.11 (0.00−0.25)
Anxiety disorders	1	89	138	0.64 (0.52−0.75)
ADHD	5	497	1261	0.53 (0.21−0.85)
Behavior disorders	3	612	1443	0.47 (0.11−0.82)
Adolescents (13-18 years)				
Any mental disorder	5	1756	5117	0.35 (0.04−0.67)
Depressive disorders	12	8193	21958	0.36 (0.25−0.46)
Anxiety disorders	4	466	2740	0.20 (0.09−0.30)
ADHD	1	369	617	0.60 (0.54−0.65)
Behavior disorders	2	625	1240	0.57 (0.25−0.89)

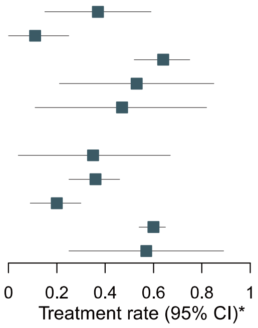

eFigure 54. Subgroup Analysis by Age Group

Outcomes	Study, No.	Events, No.	Total, No.	Treatment rate (95% CI)*
Lower middle income				
Any mental disorder	1	8	133	0.06 (0.02−0.14)
Upper middle income				
Any mental disorder	4	422	2184	0.24 (0.02−0.47)
Depressive disorders	2	48	309	0.13 (0.00−0.31)
Anxiety disorders	1	122	917	0.13 (0.10−0.17)
High income				
Any mental disorder	13	3820	8588	0.43 (0.35−0.52)
Depressive disorders	18	8601	22912	0.39 (0.29−0.48)
Anxiety disorders	8	643	2676	0.34 (0.18−0.49)

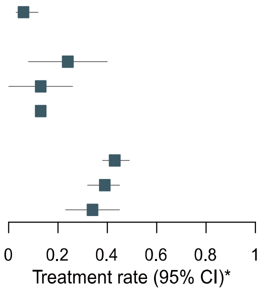

eFigure 55. Subgroup Analysis by Income Level

© 2023 Wang S et al. *JAMA Network Open.*

eMethods 1. Retrieval Strategy

According to the topic of this study, the keywords to be searched were "mental disorders", "depressive disorders", "anxiety disorders", "attention deficit hyperactivity disorder", "behavior disorders", "treatment rate", "children", "adolescent", and "seeking help". In order to find as many articles as we need, we looked for terms with the same meaning but different expressions as these keywords in other similar systematic reviews or Medical Subject Headings. The search terms were consistently used in different databases. Literature search was limited to English journals. (Take Pubmed for example, search entry available online: see eAppendix). Each retrieval step was performed by two researchers (SW and QL) independently. If there is a disagreement between the two researchers, a third party (Senior researcher, YX) will participate in discussion and resolve the inconsistencies.

eMethods 2. Exposure

Any mental disorder mainly included anxiety disorders (panic disorder, generalized anxiety disorders, agoraphobia without panic disorder, specific phobia, social phobia, posttraumatic stress disorder, obsessive-compulsive disorder, separation anxiety disorders), mood disorders (major depressive disorders, dysthymic disorder, bipolar disorder I or II), impulse control disorders (oppositional defiant disorder, conduct disorder, attention deficit hyperactivity disorder, intermittent explosive disorder), and substance use disorders (alcohol and drug abuse and dependence). This fact should arouse our attention, the partial literature collected in this study did not include all of the conditions mentioned above.

The diagnostic tools used include the Child and Adolescent Psychiatric Assessment (CAPA),[1] the Diagnostic Interview Schedule for Children (DISC-Ⅳ/ DISC 2.3),[2-3] the anxiety disorders Interview Schedule for Children-Parent Version (ADIS-C/P),[4] the World Mental Health Composite International Diagnostic Interview (CIDI; ICD-9/10),[5] the Present Episode Version of the Schedule for Affective Disorders and Schizophrenia for School-Aged Children (K-SADS),[6] the Development and Well-Being Assessment (DAWBA),[7] the Mini International Neuropsychiatric Interview for Children and Adolescents (MINI-KID),[8] and the Diagnostic Interview for Children and Adolescents (DICA).[9]

eResults. Study Selection

A total of 39251 articles were initially included. When duplicates were removed, 26350 studies were left. Two authors conducted preliminary screening according to the title and abstract of the article respectively. 338 articles were screened for full-text reading, 68 literatures were obtained. By searching the references of the 68 literatures, a total of 9 records meeting the criteria were found. If multiple studies used overlapping samples but provided unique treatment rates for diseases, all studies were retained for analysis. If multiple studies with overlapping samples assess treatment rates for the same disease, determining which study to include in the meta-analysis is based on the largest sample size and the most detailed description. After excluding 34 studies with duplicate samples and three studies with a quality score of less than 5, 40 studies were eventually included analysis.

eAppendix. Explicit Search Entry

Search term for the systematic literature in Pubmed:

#1 ((((((((((((((((((((("Help-Seeking Behavior"[Title/Abstract]) OR ("Healthcare Disparities"[Title/Abstract])) OR ("Health Services Accessibility"[Title/Abstract])) OR ("Mental Health Services"[Title/Abstract])) OR ("Community Mental Health Services"[Title/Abstract])) OR ("treatment

© 2023 Wang S et al. *JAMA Network Open.*

gap"[Title/Abstract])) OR ("treatment gaps"[Title/Abstract])) OR ("treatment rates")) OR ("treatment rate*")) OR ("under treatment"[Title/Abstract])) OR ("treatment need"[Title/Abstract])) OR ("treatment seeking"[Title/Abstract])) OR ("seeking treatment"[Title/Abstract])) OR ("health care seeking"[Title/Abstract])) OR ("seeking care"[Title/Abstract])) OR ("access to care"[Title/Abstract])) OR ("professional help"[Title/Abstract])) OR ("barriers to treatment"[Title/Abstract])) OR ("treatment barrier*"[Title/Abstract])) OR ("Patient Compliance"[Title/Abstract])) OR ("Patient Acceptance of Health Care"[Title/Abstract])) OR ("Medication Adherence"[Title/Abstract])) OR ("Treatment Adherence and Compliance"[Title/Abstract])) OR ("Treatment Adherence"[Title/Abstract])) OR ("Treatment Compliance"[Title/Abstract])

AND

#2 ((((((((((((((("Mental Disorder*"[Title/Abstract]) OR ("Psychiatric Disorder*"[Title/Abstract])) OR (Depression[Title/Abstract])) OR ("depressive disorders*"[Title/Abstract])) OR ("depressive disorders, Major"[Title/Abstract])) OR ("major depressive episode"[Title/Abstract])) OR (anxiety[Title/Abstract])) OR ("anxiety disorders"[Title/Abstract])) OR (Hypervigilance[Title/Abstract])) OR ("Generalized anxiety disorders"[Title/Abstract])) OR ("Behavior Disorder*"[Title/Abstract])) OR ("Emotional Disorder*"[Title/Abstract]))

OR ("conduct disorders"[Title/Abstract])) OR (ODD[Title/Abstract])) OR ("attention deficit hyperactivity disorder*"[Title/Abstract])) OR ("ADHD" [Title/Abstract])) OR ("Disruptive behavior disorder*"[Title/Abstract])) OR ("Mental problem"[Title/Abstract])

AND

#3 (((((((((adolescent*[Title/Abstract]) OR (teenager*[Title/Abstract])) OR (child*[Title/Abstract])) OR(children[Title/Abstract])) OR (student*[Title/Abstract])) OR (juvenile*[Title/Abstract])) OR (school*[Title/Abstract])) OR (teen*[Title/Abstract])) OR (young[Title/Abstract])) OR (youth*[Title/Abstract])) OR (minors[Title/Abstract])

eReferences

1. Angold A, Costello EJ. The Child and Adolescent Psychiatric Assessment (CAPA). *J Am Acad Child Adolesc Psychiatry*. 2000;39(1):39-48. doi: 10.1097/00004583-200001000-00015

2. Schwab-Stone ME, Shaffer D, Dulcan MK, et al. Criterion validity of the NIMH Diagnostic Interview Schedule for Children Version 2.3 (DISC-2.3). *J Am Acad Child Adolesc Psychiatry*. 1996;35(7):878-88. doi: 10.1097/00004583-199607000-00013

3. Shaffer D, Fisher P, Lucas CP, Dulcan MK, Schwab-Stone ME. NIMH Diagnostic Interview Schedule for Children Version IV (NIMH DISC-IV): description, differences from previous versions, and reliability of some common diagnoses. *J Am Acad Child Adolesc Psychiatry*. 2000;39(1):28-38. doi: 10.1097/00004583-200001000-00014

4. Wood JJ, Piacentini JC, Bergman RL, McCracken J, Barrios V. Concurrent validity of the anxiety disorders section of the Anxiety Disorders Interview Schedule for DSM-IV: child and parent versions. *J Clin Child Adolesc Psychol*. 2002;31(3):335-42. doi: 10.1207/S15374424JCCP3103_05

5. Kessler RC, Ustün TB. The World Mental Health (WMH) Survey Initiative Version of the World Health Organization (WHO) Composite International Diagnostic Interview (CIDI). *Int J Methods Psychiatr Res*. 2004;13(2):93-121. doi: 10.1002/mpr.168

6. Chambers WJ, Puig-Antich J, Hirsch M, et al. The assessment of affective disorders in children and adolescents by semistructured interview. Test-retest reliability of the schedule for affective disorders and

© 2023 Wang S et al. *JAMA Network Open.*

schizophrenia for school-age children, present episode version. *Arch Gen Psychiatry*. 1985;42(7):696-702. doi: 10.1001/archpsyc.1985.01790300064008
7. Goodman R, Ford T, Richards H, Gatward R, Meltzer H. The Development and Well-Being Assessment: description and initial validation of an integrated assessment of child and adolescent psychopathology. *J Child Psychol Psychiatry.* 2000;41(5):645-55.
8. Sheehan DV, Sheehan KH, Shytle RD, et al. Reliability and validity of the Mini International Neuropsychiatric Interview for Children and Adolescents (MINI-KID). *J Clin Psychiatry*. 2010;71(3):313-26. doi: 10.4088/JCP.09m05305whi
9. Reich W, Herjanic B, Welner Z, Gandhy PR. Development of a structured psychiatric interview for children: agreement on diagnosis comparing child and parent interviews. *J Abnorm Child Psychol*. 1982;10(3):325-36. doi: 10.1007/BF00912325

© 2023 Wang S et al. *JAMA Network Open.*
