# Supplementary material for: Treatment Rates for Mental Disorders Among Children and Adolescents: A Systematic Review and Meta-Analysis
Source: JAMA Netw Open. 2023 Oct 18;6(10):e2338174. doi: 10.1001/jamanetworkopen.2023.38174 (PMC10585417; doi:10.1001/jamanetworkopen.2023.38174)
Supplement: Supplement 2. — Data Sharing Statement [file jamanetwopen-e2338174-s002.pdf]

Data Sharing Statement

Wang. Treatment Rates for Mental Disorders Among Children and Adolescents. *JAMA Netw Open*. Published October 18, 2023. doi:10.1001/jamanetworkopen.2023.38174

Data
Data available: Yes
Data types: Data (not involving human participants)
How to access data: Will be available from the corresponding author under request.
When available: With publication

Supporting Documents
Document types: None

Additional Information
Who can access the data: researchers whose proposed use of the data has been approved
Types of analyses: for any purpose
Mechanisms of data availability: after approval of a proposal
